# Target-enriched long-read sequencing (TELSeq) contextualizes antimicrobial resistance genes in metagenomes

**DOI:** 10.1186/s40168-022-01368-y

**Published:** 2022-11-02

**Authors:** Ilya B. Slizovskiy, Marco Oliva, Jonathen K. Settle, Lidiya V. Zyskina, Mattia Prosperi, Christina Boucher, Noelle R. Noyes

**Affiliations:** 1 Food-Centric Corridor, Infectious Disease Laboratory, Department of Veterinary Population Medicine, College of Veterinary Medicine, University of Minnesota, St. Paul, MN USA; 2 Department of Computer and Information Science and Engineering, Herbert Wertheim College of Engineering, University of Florida, Gainesville, FL USA; 3 Program in Human-Computer Interaction, College of Information Studies, University of Maryland, College Park, MD USA; 4 Data Intelligence Systems Lab, Department of Epidemiology, College of Public Health and Health Professions and College of Medicine, University of Florida, Gainesville, FL USA

**Keywords:** Metagenomics, Microbiome, Long-read sequencing, Antimicrobial resistance, Resistome, Public health, Mobile genetic elements

## Abstract

**Background:**

Metagenomic data can be used to profile high-importance genes within microbiomes. However, current metagenomic workflows produce data that suffer from low sensitivity and an inability to accurately reconstruct partial or full genomes, particularly those in low abundance. These limitations preclude colocalization analysis, i.e., characterizing the genomic context of genes and functions within a metagenomic sample. Genomic context is especially crucial for functions associated with horizontal gene transfer (HGT) via mobile genetic elements (MGEs), for example antimicrobial resistance (AMR). To overcome this current limitation of metagenomics, we present a method for comprehensive and accurate reconstruction of antimicrobial resistance genes (ARGs) and MGEs from metagenomic DNA, termed *t*arget-*e*nriched *l*ong-read *seq*uencing (TELSeq).

**Results:**

Using technical replicates of diverse sample types, we compared TELSeq performance to that of non-enriched PacBio and short-read Illumina sequencing. TELSeq achieved much higher ARG recovery (>1,000-fold) and sensitivity than the other methods across diverse metagenomes, revealing an extensive resistome profile comprising many low-abundance ARGs, including some with public health importance. Using the long reads generated by TELSeq, we identified numerous MGEs and cargo genes flanking the low-abundance ARGs, indicating that these ARGs could be transferred across bacterial taxa via HGT.

**Conclusions:**

TELSeq can provide a nuanced view of the genomic context of microbial resistomes and thus has wide-ranging applications in public, animal, and human health, as well as environmental surveillance and monitoring of AMR. Thus, this technique represents a fundamental advancement for microbiome research and application.

Video abstract

**Supplementary Information:**

The online version contains supplementary material available at 10.1186/s40168-022-01368-y.

## Introduction

Metagenomic sequencing offers comprehensive access to the genetic material of microbial ecosystems, providing critical insight into the functioning of diverse microbe-dependent environments such as the human gut [1], plant rhizospheres [2], and the ocean [3]. However, metagenomic sequencing suffers from relatively low sensitivity, especially for genomes and genes that have low abundance within a microbial community [4–6]. This low sensitivity manifests in several ways, including inability to reconstruct low-abundance genomes [7] and failure to detect low-abundance genes and genomes that are known to be present within a sample [8]. This latter limitation is especially relevant for genes such as antibiotic resistance genes (ARGs), mobile genetic elements (MGEs), and pathogen cargo genes which often comprise <1% of metagenomic DNA [4–6]. Furthermore, ARGs with the highest public health and clinical importance tend to comprise the low-abundance ARGs within resistomes [8, 9], which limits the utility of metagenomics for health applications related to antimicrobial resistance.

Several techniques can be used to correct for the low sensitivity of the metagenomic approach. Many of them have focused on detecting viruses, which tend to comprise a low proportion of metagenomic data [10]. In this context, tiling multiplex PCR has been successful in enriching for specific viruses in samples with low titers [11] but this method is only capable of enriching a small number of genomes in parallel. Probe-based capture techniques can overcome this limitation by supporting non-PCR-based parallel enrichment of hundreds of targets, and this approach has been used successfully for highly sensitive virome analysis [12, 13] as well as for highly sensitive detection of ARGs from diverse samples [8, 9]. More specifically, probe-based enrichment increased the proportion of sequenced reads originating from ARGs by more than three orders of magnitude, which doubled the detectable resistome richness and revealed numerous critical yet low-abundance ARGs that had gone undetected in non-enriched metagenomic sequence data [8, 9].

However, all previous capture enrichment studies used short-read sequencing platforms such as the Illumina HiSeq or NovaSeq [14–16]. Short-read metagenomic data are known to produce highly fragmented assemblies with extreme coverage variability [17–19]. This limitation is especially relevant for ARGs, which often contain phenotypically meaningful variants, and which are commonly located near MGEs. Colocalization with MGEs facilitates ARG lability within and across genomes, enabling emergence and rapid dissemination of pathogens with highly resistant ARG variants that threaten the efficacy of last-resort antibiotics [20–22]. The limitations of short-read data to identify ARG variants and their immediate genomic context greatly diminish the applied relevance of metagenomics [23, 24].

Several methods exist to provide improved variant detection and genomic contextualization of metagenomic data but they all have critical shortcomings. For example, guided ligation-based sequencing improves variant profiling [25], but has not been used to enrich microbial DNA or resolve numerous flanking targets within microbiomes. Proximity ligation (Hi-C) [26] and microfluidic sequencing [27] can be used to reconstruct the genomic context of targets such as ARGs, but these methods do not include target-specific enrichment steps, and therefore can only reconstruct the more abundant components of a metagenomic sample. Linked-read sequencing has been used to improve short-read metagenomic assemblies but the assembly quality (i.e., genome completeness and presence of contamination in the assembly) does not rival that of long-read sequences [19]. Functional metagenomics has been used to discover novel ARGs and characterize their immediate genomic context [28], but this method relies on the creation of massive culture-based functional libraries, which is very laborious and only semi-quantitative.

While long-read sequencing could fill the current void in producing highly informative raw sequence data, the relatively low throughput of long-read sequencers makes for even lower sensitivity to detect rare metagenomic targets [29]. Theoretically, Nanopore-based real-time adaptive sequencing could be used to enrich on-target DNA [30, 31]. This method rejects off-target DNA, thus only allowing on-target DNA to be sequenced. However, the current approach does not extend well to ARGs within metagenomic data for two main reasons: first, the off-target DNA is difficult to characterize (i.e., all non-ARG bacterial, fungal, viral, protozoal and host(s) DNA), and second, the genomic plasticity of ARGs makes it difficult to predict whether the sequence in the pore will eventually contain an ARG [30, 32]. Thus, despite intensive activity in this area, a critical need remains for highly sensitive sequencing of metagenomic targets that can support robust characterization of variants and their immediate flanking regions.

**Fig. 1:**
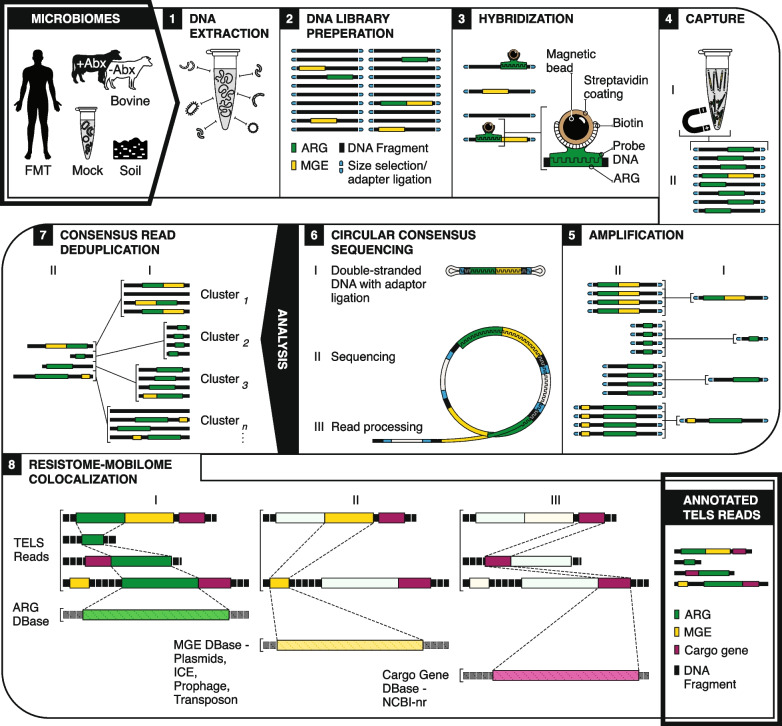
TELSeq workflow overview. Utilizing replicates of diverse sample types, gDNA was extracted (1), and sheared, fragmented, size selected, A-tailed and adapter ligated (2). Then, custom-designed biotinylated 120-mer probes (3) were used to capture ARGs with streptavidin-coated magnetic beads (4). Captured fragments were amplified and purified (5) and submitted for PacBio CCS (6). Resulting TELSeq reads were deduplicated to correct for amplification bias (7). Finally, reads were aligned to numerous reference databases to identify and annotate ARGs, MGEs, and cargo genes (8)

In this work, we demonstrate that cRNA biotinylated probes can be used to capture relatively long fragments of DNA, generating enriched long-read sequence data with high information value. We term this approach TELSeq (target-enriched long-read sequencing) and evaluate its performance using replicates from diverse and challenging sample matrices, as well as a control mock community (Fig. 1). We illustrate that TELSeq achieves highly sensitive detection of very low-abundance ARGs from complex matrices, while also providing robust colocalization of ARGs with nearby MGEs. Using this high-value information, we observe numerous novel and previously confirmed ARG-MGE colocalizations, indicating the utility of TELSeq to inform risk assessment using metagenomic data and to support further understanding of the extent and diversity of ARG-MGE associations within and across different bacteria. In consequence, we show that TELSeq supports novel discovery and improved understanding of microbiome-wide processes across a wide range of biological applications.

## Results

### Combining target enrichment with long-read sequencing improved ARG detection in samples with low ARG abundance

We benchmarked TELSeq performance using technical replicates of three sample types presumed to have a relatively low abundance of target (i.e., ARG) DNA: human feces donated for fecal microbiome transplant material (“FMT”); fecal material from a cow that did not receive antibiotics (“−Abx”); and soil from an unused prairie easement surrounded by farmland (“SOIL”). Three technical replicates of each low-ARG sample type were each subjected to three sequencing approaches: ARG target-enriched long-read CCS sequencing using PacBio (TELSeq), non-enriched long-read CCS sequencing on the PacBio (“PB”), and Illumina short-read sequencing on the NovaSeq 6000 (“SR”). Resulting sequence data were analyzed for ARG abundance and diversity to compare resistome profiles within and across replicates, sample types, and sequencing platforms.

**Fig. 2:**
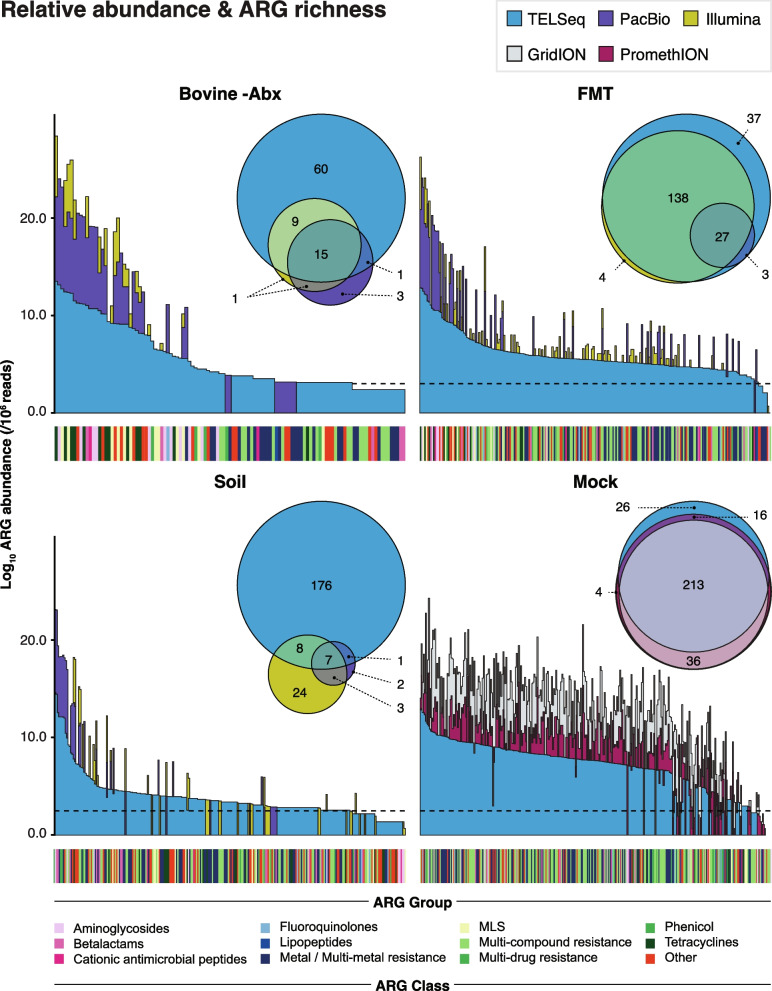
ARG abundance and richness. Stacked bar plots depict the relative abundance (*y*-axis) of unique ARG groups across technical replicates of each sample type (*x*-axis), with each ARG group count normalized for sequencing depth and expressed on a 10^6^ read basis generated by each sequencing platform (TELSeq= light blue, PacBio= purple; Illumina= yellow; GridION= gray; PromethION= magenta). Final relative abundances are scaled using a log_10_ transformation. Rug colors on the *x*-axis of each plot indicate the MEGARes class to which each ARG belongs. The “Other” classification refers to drug classes present in <15 % of ARG hits by alignment, including aminocoumarins, bacitracin, biocides, elfamycin, fosfomycin, glycopeptides, nucleosides, oxazolidinone, pleuromutilin, quaternary ammonium compounds, trimethoprim / sulfas, and *Mycobacterium tuberculosis* drugs. Inset Venn diagrams indicate ARG group-level richness and composition, compared between sequencing platforms

Use of TELSeq increased the proportion of reads containing targeted ARGs, i.e., “on-target” reads, from ~1% in the PB replicates to 14–49% in the TELSeq replicates (Supp Table 1). The increase in on-target proportion differed by sample type, with SOIL technical replicates exhibiting the largest increase from 0.2% in PB data to >25% in TELSeq data. Increases in the −Abx and FMT technical replicates were more modest, from ~1% in PB data to >14% in TELSeq data; however, even with this modest increase in on-target rate, TELSeq still identified many ARGs that were not detected via PB and SR sequencing (i.e., 60 and 37 unique ARGs in the −Abx and FMT replicates, respectively, Fig. 2). There was variability in TELSeq on-target proportion between technical replicates within the same sample, ranging from 14 to 19% for FMT, 17 to 33% for −Abx, and 26 to 49% for SOIL (Supp Table 1). These on-target proportions were significantly correlated with overall sequencing depth, with more deeply sequenced technical replicates attaining higher on-target proportions (Type III ANOVA *P* < 0.005).

In addition to higher on-target sequencing efficiency, the use of TELSeq enabled more complete detection of the full ARG profile (i.e., the resistome richness) within each sample (Fig. 2), despite significantly lower read depth than the SR datasets and comparable read depth to the PB datasets, based on ANOVA results. At the ARG group level, the detected richness in TELSeq data was at least double that in PB and SR data, and in some cases nearly 10 times greater (Fig. 2, Supp Table 2). Even at the class level, which has low resolution based on ARG classification hierarchy, TELSeq data consistently contained higher richness than PB and SR data, across all samples and replicates (Supp Table 2). For the FMT and SOIL replicates, the PB platform yielded the lowest resistome richness, while the SR platform yielded the lowest richness for the −Abx replicates. As with on-target rate, there was variability in the number of unique ARG classes detected between technical replicates within each sample type; replicates with a higher number of reads had significantly higher class-level ARG richness, although this association was not statistically significant at the predefined cutoff (Type III ANOVA *P* = 0.055).

The expanded resistome richness uncovered by TELSeq occurred largely due to detection of additional ARG groups not detected using PB and SR platforms. For FMT and −Abx replicates, use of TELSeq resulted in successful recovery of nearly all of the ARG groups detected using PB and SR platforms, plus an additional 37 and 60, respectively, not detected with the other platforms (Fig. 2). For SOIL, TELSeq identified 176 ARG groups not detected by PB and SR, although 24 ARG groups contained in the SR datasets were not detected in TELSeq data.

### TELSeq enabled recovery of very rare ARGs

**Fig. 3:**
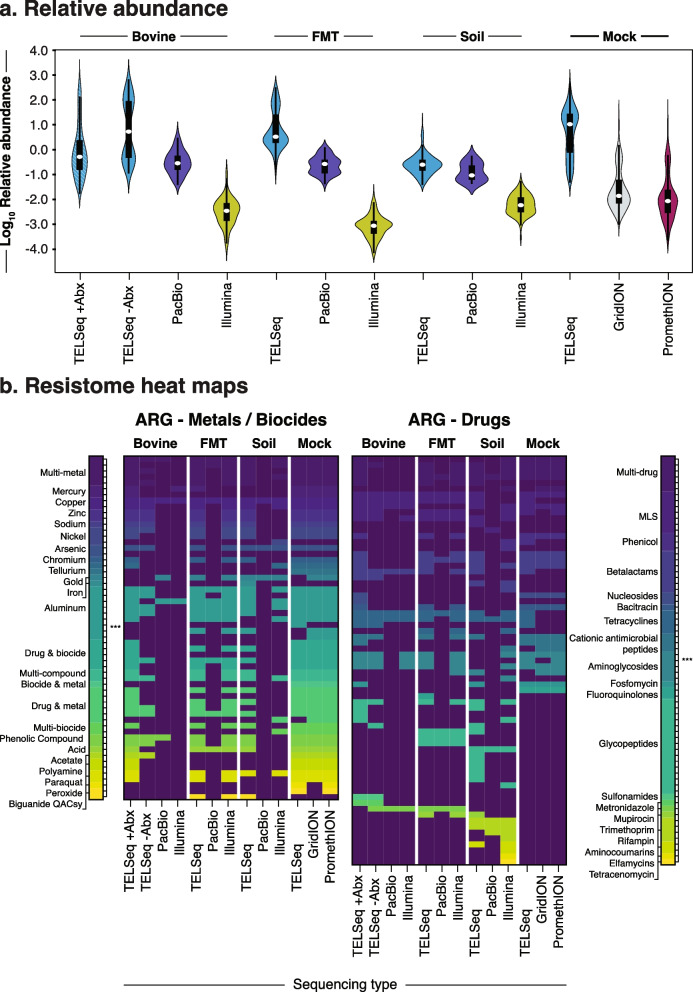
Resistome distribution and composition. **a** Violin plots showing resistome distribution as the log_10_ relative abundance of ARG groups (*y*-axis), normalized for gene length and sequencing depth, by sample type and sequencing platform. **b** Binary heatmap of resistome composition at the ARG mechanism level, for metals and biocides (left) and antibiotic drugs (right), by sample type and sequencing platform

The additional ARGs detected by TELSeq comprised the low-abundance ARGs within each sample (Fig. 2). The inclusion of these low-abundance ARGs significantly changed the resistome profile (ANOSIM *P*< 0.001; PERMANOVA *R*^*2*^ = 0.08, *P*= 0.01), demonstrating that the ARG profile detected by TELSeq differed significantly from that of PB and SR (Supp Figure 1). The variability by sequencing platform, however, was much smaller than the variability between sample type (PERMANOVA *R*^*2*^ = 0.08 and 0.55, *P*= 0.001 and 0.01, respectively), again indicating that the additional ARGs detected by TELSeq comprised the “tail” of the resistome distribution for each sample (Fig. 2). In contrast, high-abundance ARGs were consistently detected by all three sequencing platforms (Fig. 2). These high-abundance ARGs differed between the samples. FMT and −Abx replicates were dominated by ARGs that confer resistance to tetracycline and macrolide-lincosamide-streptogramin B (MLS) antibiotics, while the SOIL replicates were dominated by a more diverse ARG repertoire, including ARGs that confer resistance to antibiotics less commonly used in animals and humans, including cationic antimicrobial peptides, oxazolidinones, and rifampin (Figs. 2 and 3). TELSeq’s detection of the low-abundance portion of the resistome also created a detectable shift in the observed ARG distribution, with TELSeq libraries exhibiting a much wider distribution of ARG relative abundances compared to PB and SR libraries (Fig. 3).

### TELSeq revealed ARG context, including colocalization with MGEs

**Fig. 4:**
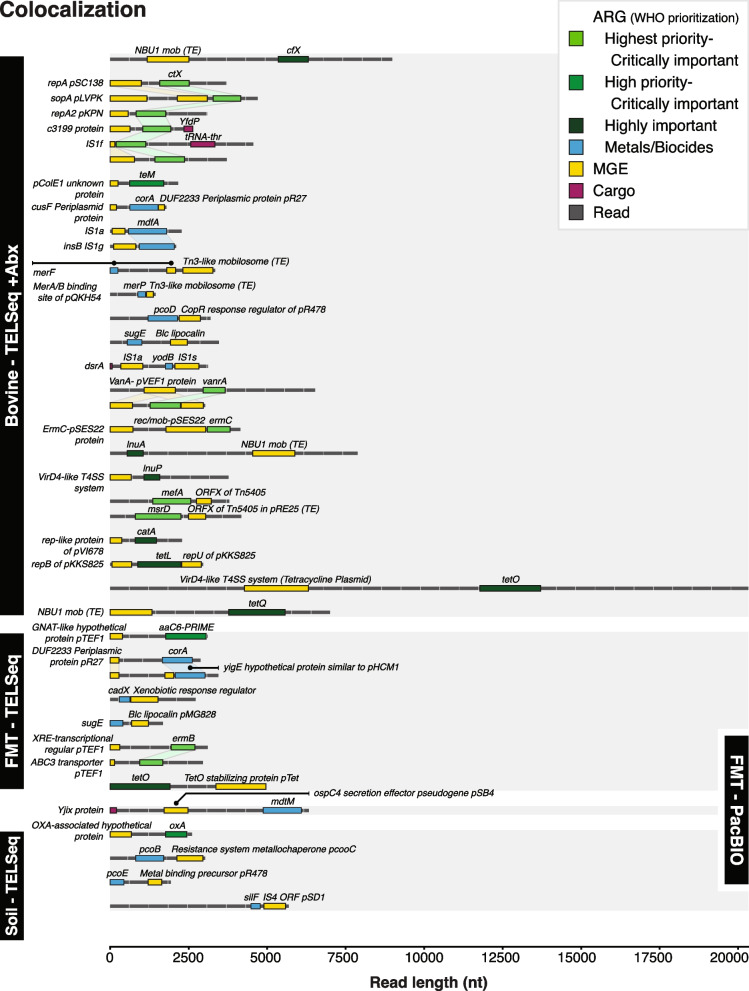
ARG-MGE colocalizations in TELSeq reads. Individual TELSeq reads (black horizontal dashed lines, length on *x*-axis) containing both ARGs (green and blue) and MGEs (yellow), as well as cargo genes (red), separated by sample type (*y*-axis). Color of ARG groups indicates the World Health Organization’s (WHO) classification status. Light green: highest priority, critically important. Medium green: high priority, critically important. Dark green: highly important. The −Abx sample did not contain any ARG-MGE colocalizations

In addition to evaluating TELSeq’s ability to capture and enrich ARG targets, we also evaluated whether biologically relevant information could be obtained from the ARG-flanking regions of the generated long-read data. To do this, we analyzed both TELSeq and PB data by annotating the flanking regions of ARG-containing reads for the presence of MGEs and cargo genes. In the SOIL and FMT replicates, TELSeq data contained numerous such colocalizations, whereas the PB data contained only one colocalization in the FMT sample, and none in the SOIL (Table 1 and Fig. 4). No colocalizations were identified in either the TELSeq or the PB replicates from the −Abx sample.

**Table 1:**
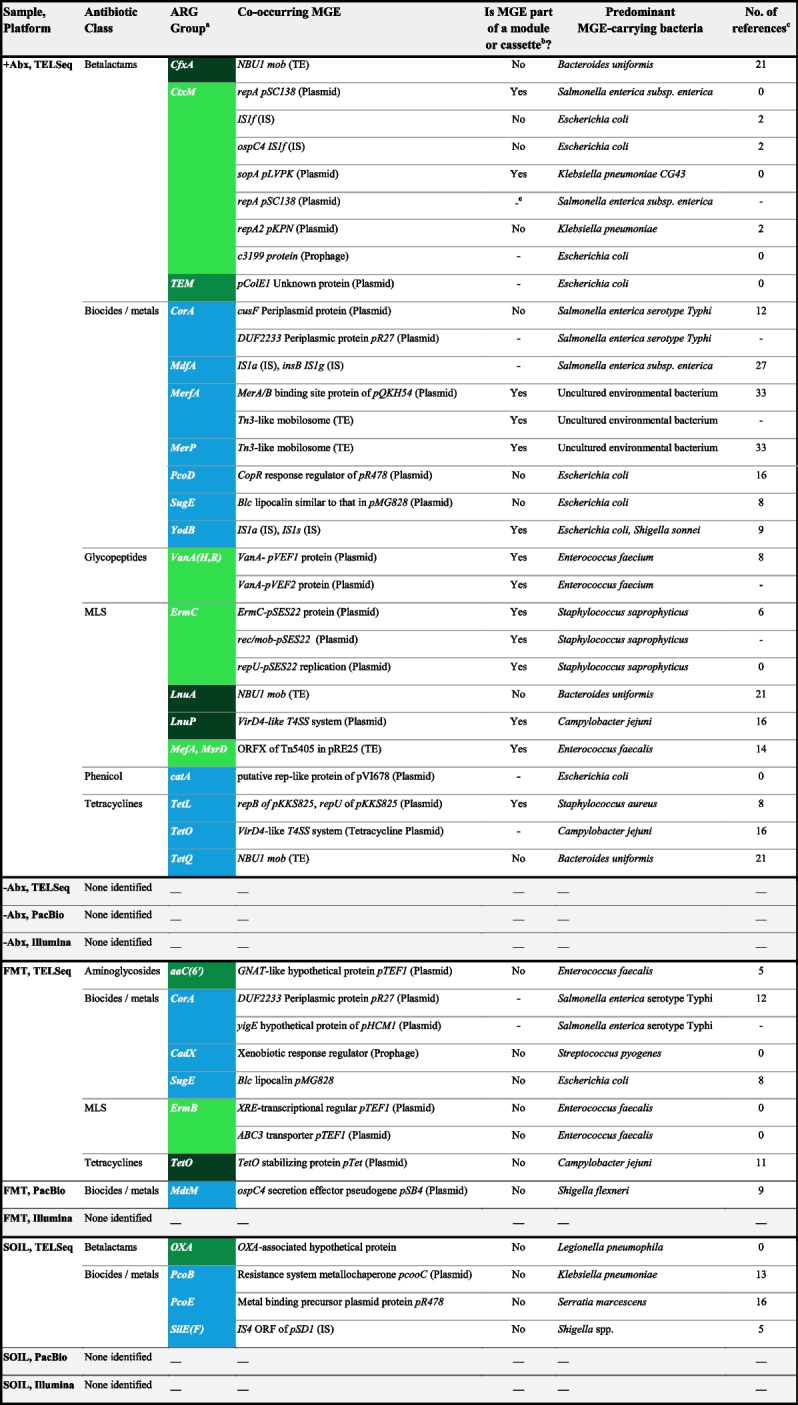
ARG-MGE colocalization details. Each row indicates a unique ARG-MGE colocalization, grouped by antibiotic class, sequencing platform, and sample type

^a^Color of ARG groups indicate the World Health Organization’s (WHO) classification status. Light green: Highest priority, critically important; Medium green: High priority, critically important; Dark green: Highly important; Blue: Metal or biocide resistance, not classified by WHO

^b^Indicates whether the colocalized MGE has been identified in a reference MGE that also contains at least one architectural MGE gene responsible for functional integration, replication, or housekeeping

^c^Number of references identified using PubMed, with the following specifications: primary literature as the subject of experimental or clinical research results, or used as support of research findings. Only publications that directly referenced the accession, locus name, or GenBankID for the relevant MGE were considered

^d^“-” indicates that the information is unknown

In the TELSeq FMT replicates, ARGs conferring resistance to aminoglycoside, betalactam, macrolide-lincosamide-streptogramin B (MLS), and tetracycline antibiotics were colocalized with plasmids, while the TELSeq SOIL data contained a betalactam ARG colocalized with a poorly characterized hypothetical plasmid protein associated with *bla*_*OXA*_ (Table 1). These antibiotics have received the World Health Organization’s highest rating for their importance to human medicine (i.e., “critically important” classification), with the exception of tetracycline which is classified in the second-highest tier, i.e., “highly important” (Table 1) [33]. Most of the ARG-associated MGEs within the TELSeq data have been documented, although many are poorly characterized as evidenced by a very small number of relevant references (Table 1).

The three sample types used to analyze the performance of TELSeq (i.e., SOIL, FMT, and −Abx) were all purposively selected based on a presumed very low ARG abundance, in order to evaluate the ability of TELSeq to capture rare targets. Therefore, to investigate TELSeq’s ability to identify mobilized ARGs within a more clinically relevant sample, we performed TELSeq and colocalization analysis on technical replicates of a fecal sample obtained from a cow that had recently received intensive systemic antibiotic treatments (“+Abx” sample). As expected, this sample contained more ARGs (Fig. 3) and colocalizations (Table 1 and Fig. 4) than the −Abx, FMT, and SOIL samples. Specifically, 30 unique colocalizations were identified in the +Abx sample compared to none in the −Abx sample, 8 in the FMT sample, and 4 in the SOIL sample (Table 1). In addition to mobilized ARGs that confer resistance to aminoglycosides, betalactams, MLS, and tetracyclines, we also detected colocalized ARGs that confer resistance to glycopeptides and phenicols, which are classified as “critically important” and “highly important,” respectively. Unlike the low-abundance ARG samples, the +Abx sample colocalizations included diverse MGEs such as plasmids, transposable elements (TE), insertional sequences (IS), and prophages (Table 1). Nearly half of the colocalizations in the +Abx sample included sequences that form the foundation of conjugative transfer, including plasmid replication sites, mating pair formation genes (e.g., VirD4 for type IV secretion systems), and integrase systems. Some ARGs were colocalized with multiple different MGEs within the +Abx sample, which was not observed in the other sample types (Table 1). While most of the colocalization-containing TELSeq reads were <5000bp, we also recovered several reads >10,000bp (Fig. 4).

The ability of TELSeq to identify MGEs was further evaluated by comparing the mobilome of TELSeq data to that of PB and SR. MGEs were considered positively identified in the dataset if they obtained at least 50% gene fraction within one replicate. Based on this analysis, the SR data contained higher richness of MGEs as compared to the TELSeq and PB methods (Supp Figure 2). However, the relative abundance of MGE-containing reads was higher for the PB and TELSeq methods as compared to SR (Supp Figure 2). In addition, TELSeq data for the −Abx and SOIL replicates contained a higher richness of MGE subtypes compared to the PB datasets, even though the TELSeq probes were not designed to capture and enrich MGEs. In the −Abx replicates, both TELSeq and PB identified a comparable set of plasmid and prophage accessions; however, only TELSeq detected a large number of IS accessions. In the SOIL replicates, both TELSeq and PB recovered a similar set of plasmids, prophages, IS, and virulence accessions, but only TELSeq data contained an additional fraction of ICE, which were missed by the PB approach (Supp Figure 2).

### TELSeq’s performance was robust for targets with relative abundance greater than 10^−4^

To robustly and systematically evaluate TELSeq’s sensitivity, specificity, and coverage depth, we performed TELSeq on three replicates of the ZymoBIOMICS log-abundance mock community (“MOCK”), fragmented to median lengths of 2, 5, and 8 kb. To estimate TELSeq’s limit of detection, we identified ARGs in the reference genomes for each organism in the commercial mock community, and used these as a ground truth ARG set against which to compare the ARGs recovered by TELSeq. No ARGs were identified in the genomes of *L. fermentum, Cryptococcus neoformans*, and *Saccharomyces cerevisiae*, and thus these organisms were not included in limit of detection analysis. TELSeq recovered >90% of the ground truth ARGs for *L. monocytogenes*, *B. subtilis*, *E. coli*, and *S. enterica* across all 3 replicates (Supp Table 3). The results for *P. aeruginosa* were lower and more variable, ranging from 43 to 67%, even though this organism was present at 8.9% relative abundance in the commercial mock community. TELSeq’s performance for the two lowest-abundance organisms (i.e., *E. faecalis* and *S. aureus*) was poorest, at 33% and 1–20% of ARGs recovered, respectively. Fragment length was not systematically associated with differences in ARG recoverability (Supp Table 3).

To evaluate the probe-level sensitivity and specificity of TELSeq, we compared MOCK results to previously published high-depth (GridION) and ultra high-depth (PromethION) long-read sequencing experiments of the same mock community [34]. Each dataset (TELSeq replicates, GridION, and PromethION) and the 120-mers of the ARG probe set were aligned to the reference genome of each of the 10 organisms comprising the mock community, and coverage profiles were generated and analyzed.

The majority of probes aligned to homologous regions of *Escherichia coli*, *Salmonella enterica*, and *Pseudomonas aeruginosa*, which in total accounted for 84.5% of the probe set (Supp Table 3). The remaining fraction of bases covered by probes included all of the Gram positive species: *Staphylococcus aureus* (6.40 %), *Enterococcus faecalis* (4.90 %), *Bacillus subtilis* (2.98%), and *Listeria monocytogenes* (1.21%). No probes aligned to the genomes of *Lactobacillus fermentum* or the two eukaryotes *Cryptococcus neoformans* and *Saccharomyces cerevisiae*, as expected given the lack of ARGs in these organisms (Supp Figure 3).

**Fig. 5:**
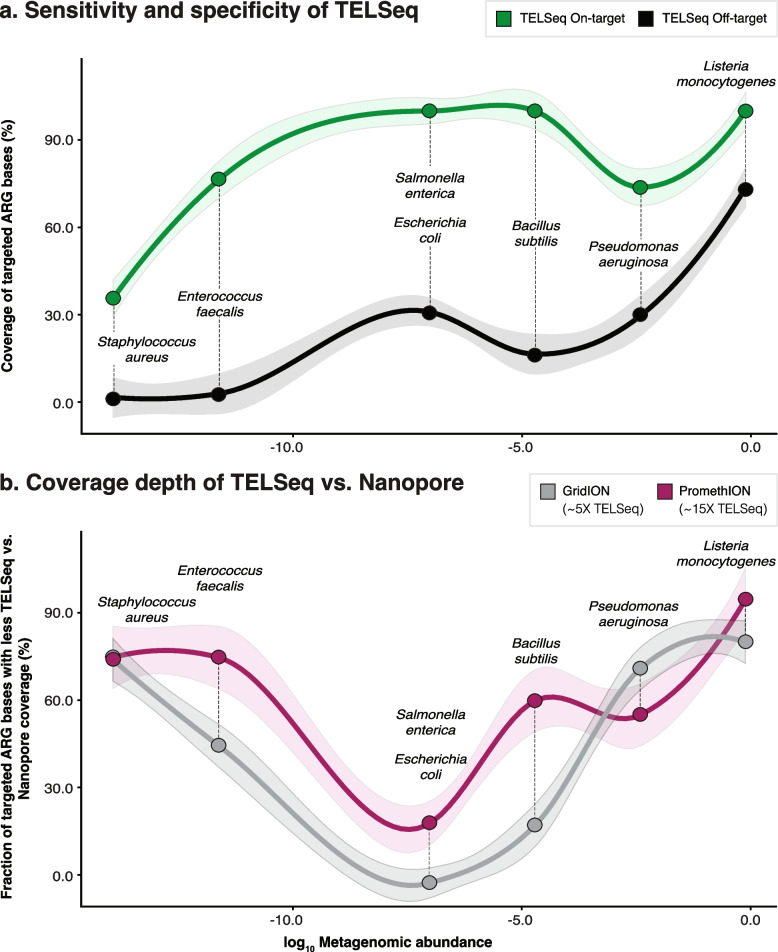
**a** Proportion of probe-covered bases that received TELSeq read coverage of at least 1× (on-target, green line), versus proportion of non-probe-covered bases that received TELSeq read coverage of at least 1× (off-target, black line). **b** Proportion of probe-covered bases for which high-depth GridION (gray line) and ultra high-depth PromethION (purple line) read coverage exceeded that of TELSeq coverage. Parentheses in figure legend indicate the relative sequencing throughput difference of GridION and PromethION platforms relative to TELSeq, i.e., 5× and 15×. Organisms in MOCK are listed in order of ascending relative abundance, from left to right on the *x*-axis. *L. fermentum*, *C. neoformans*, and *S. cerevisiae* did not have any known ARGs and did not receive any probe-specific coverage; therefore, the on-target rates by TELSeq (**a**) and Nanopore are not calculable. *L. fermentum*, *C. neoformans*, and *S. cerevisiae* received 17,922, 1242, and 571 alignments by TELSeq respectively, which can be considered off-target alignments. However, none of these off-target reads aligned to any MEGARes accessions (i.e., false positive ARGs) across any of the MOCK replicates

To evaluate TELSeq’s probe-level sensitivity, we calculated the proportion of targeted bases covered by at least one TELSeq read. TELSeq achieved 100% sensitivity for *E. coli*, *S. enterica*, *B. subtilis*, and *L. monocytogenes* across all three fragment lengths; 65–86% for *P. aeruginosa*; 73–79% for *E. faecalis*; and 30–45% for *S. aureus* (Supp Table 3). *L. fermentum* and the two eukaryote genomes did not have any probe-targeted loci in their genomes, and therefore we could not evaluate probe-level sensitivity for these organisms. There was no consistent relationship between probe sensitivity and the relative abundance of each organism in the sample, although *S. aureus* was the least abundant organism and also achieved the lowest sensitivity (Supp Figure 3). To evaluate the specificity of TELSeq probes, we calculated the proportion of non-probe-targeted bases in each genome that did not receive any TELSeq reads (Supp Table 3). Based on this analysis, specificity was highest for *S. aureus* (98%), and lowest for *L. monocytogenes* (26–27%), which reflects a positive relationship between genome relative abundance and the likelihood that probes bound to off-target DNA (Fig. 5a), independent of shearing size (Type III ANOVA *P*= 0.002). The specificity results for *P. aeruginosa* did not follow this trend, and the sensitivity results were similarly aberrant. TELseq generated 17,922, 1242, and 571 reads which mapped to the reference genomes of *Lactobacillus fermentum*, *Cryptococcus neoformans*, and *Saccharomyces cerevisiae,* respectively, despite the fact that no probes aligned to these three genomes. Thus, these sparse TELSeq read alignments are likely the result of off-target probe binding, which is further supported by the fact that none of the reads aligned to ARGs in the MEGARes reference database. Interestingly, all of the alignments to *Cryptococcus neoformans* were clustered at a specific locus within the genome, which was also the only locus within this genome that received sequencing coverage in the GridION and PromethION data (Supp Figure 3).

**Fig. 6:**
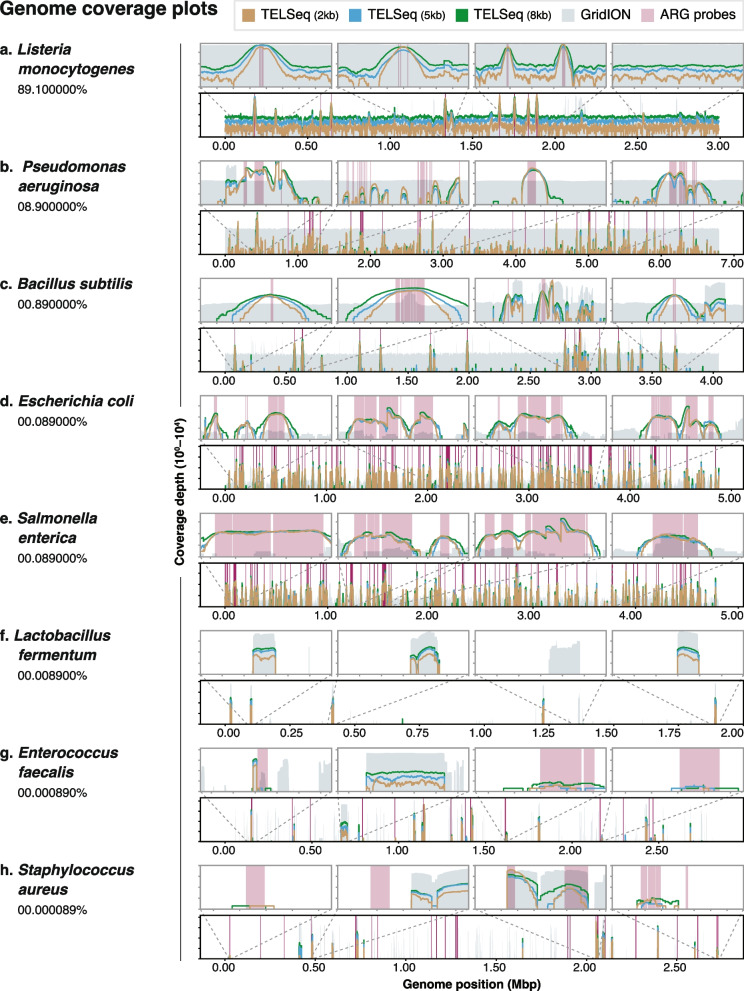
Sequencing coverage of genomes in MOCK. Genomes are arranged on the *y*-axis in descending order of relative abundance (**a**–**h**), with the log_10_ relative abundance of each genome in MOCK displayed beneath the genome name. Coverage depth ranging from 10^0^ to 10^4^ is displayed on the *y*-axis and genome position (Mbp) on the *x*-axis for each genome. Areas of probe coverage are colored pink. Sequencing coverage achieved by deep GridION sequencing is indicated in gray, while TELSeq coverage is indicated in tan (2 kb library), blue (5 kb library), and green (8 kb library). Zoomed-in subsets from select loci of each genome are included for visualization purposes. Plots for the two eukaryotic genomes in MOCK can be found in Supp Table 3

Single-locus ARG variants can cause clinically important outcomes in AMR infections, and therefore, it is important to call ARG SNPs with high confidence. TELSeq represents an advantage in this regard, because unlike PCR and other targeted approaches, TELSeq probes can tolerate a high probe-to-target sequence mismatch rate, and still bind to their targets. This gives TELSeq the ability to capture novel ARG variants which might have biological significance, provided that TELSeq can generate sufficient coverage for robust variant calling. Therefore, we evaluated the ability of TELSeq to generate *deep* coverage of ARG targets by comparing TELSeq read depth to that of GridION and PromethION from the same mock community [34]. Specifically, we calculated the proportion of total targeted bases for which TELSeq coverage exceeded that of deep long-read sequencing. Despite much lower data output (Supp Table 1), the TELSeq libraries exceeded the coverage of GridION deep sequencing in 99 and 96% of targeted bases in the *E. coli* and *S. enterica* genomes, respectively, regardless of fragment size (Fig. 5b, Supp Table 3). TELSeq coverage surpassed that of GridION sequencing in an average of 78%, 50%, 24%, 21%, and 15% of targeted bases in *B. subtilis*, *E. faecalis, P. aeruginosa*, *S. aureus*, and *L. monocytogenes* genomes, respectively. The percent of bases with excess TELSeq coverage was expectedly lower when comparing to the PromethION ultra-deep data (Fig. 5b), with 81%, 77%, 46%, 45%, and 27% of targeted bases in *S. enterica, E. coli, E. faecalis*, *B. subtilis*, and *S. aureus* receiving more TELSeq reads than PromethION reads (Supp Table 3). The genome of *P. aeruginosa* had 32% of targeted bases covered by an excess of TELSeq versus PromethION reads, which was actually higher than the 24% as compared to GridION reads. Finally, none of the targeted bases in the *L. monocytogenes* genome had an excess of TELSeq versus PromethION coverage (Supp Table 3). In regions where TELSeq read depth did not exceed that of GridION and/or PromethION, the depth tended to be equivalent (Fig. 6), demonstrating that TELSeq generally produced equivalent or superior coverage as compared to deep and ultra-deep long-read sequencing, despite much lower sequencing output (Supp Table 1). Variability in sensitivity, specificity, and coverage depth by fragment length (i.e., 2kb, 5kb, 8kb) was not consistent across microbial genomes.

## Discussion

### TELSeq enables improved assessment of ARG risk in metagenomic samples

In this work, we demonstrated that low-abundance ARGs within complex metagenomic samples can be detected with high sensitivity using bait enrichment combined with long-read sequencing. While the benefits of biotinylated probes for metagenomic ARG enrichment have been previously reported [8, 9, 16], the use of short-read sequencing prevented robust assessment of the ARGs’ genomic context [17]. By combining target enrichment with long-read sequencing, we discovered that numerous ARGs were colocalized with MGEs known to transfer between pathogens (Table 1, Fig. 4). This additional information is critical for risk assessment of metagenomic samples, particularly for clinical uses such as FMT [35, 36], which has previously resulted in fatal AMR infections in compromised recipients [37]. We identified several unique MGE-associated ARGs in fecal material from an FMT donor, despite the use of highly regulated screening standards [38] to prevent the introduction of multidrug-resistant organisms (MDROs) and other priority pathogens. The presence of ARGs and MGEs in FMT (Figs. 2 and 3, Supp Figure 2) is consistent with a recent analysis of ARG risks in FMT metagenomes [39] and suggests that TELSeq could be implemented as a microbiome-wide screen for the mobilizable ARG profile of FMT material. Indeed, the historical inability to confidently link ARGs with MGEs and host bacteria has been identified as a critical barrier to applied use of metagenomic data [39]. From this vantage point, TELSeq could represent an important advancement in the field of metagenomics, although further validation and standardization of the workflow is warranted for specific use cases such as this.

In performing colocalization analysis, we observed differences in the MGE-ARG profile of replicates obtained from SOIL as compared to replicates obtained from fecal material (Fig. 3, Supp Figure 1), even though the fecal material was obtained from hosts that had not been recently exposed to antibiotics (i.e., FMT and −Abx). For the latter sample types, most of the colocalized ARGs confer resistance to antibiotic drugs, and the colocalized MGEs have been predominantly characterized in ESKAPEE pathogens (Table 1), which are known for their capacity to mobilize multidrug resistance in clinical contexts. For this reason, the ESKAPEE pathogens have been listed as critical-priority bacteria by the World Health Organization [40]. Conversely, the SOIL replicates were predominated by metal and biocide ARGs colocalized with MGEs reported primarily in bacteria that are ubiquitous within environmental samples (e.g., soil, sediment, and water), including *Legionella*, *Klebsiella*, and *Serattia* (Table 1). Linkages between environmental and human-associated resistomes have been previously observed [41], although the limitations of short-read data were later found to be significantly confounding these results [42].

It is very difficult to robustly reconstruct MGEs and their colocalized cargo from metagenomic data, and this difficulty has been identified as a critical barrier to improved understanding of bacterial evolution [43, 44]. Bioinformatic reconstruction of MGEs via short-read metagenomic assembly often results in misassembly and unresolvable sequence loci, largely due to the modular and mobile nature of MGEs, which result in frequent rearrangements, deletions, insertions, and repeats [45–48]. Many metagenomic bioinformatics tools were developed for the purposes of genome classification and taxonomic resolution and have not been validated for MGE reconstruction [49, 50]. Though not a primary aim of this study, our results demonstrate that TELSeq could be used to selectively capture, enrich, and characterize MGEs and related genomic detail. Coupled with recent advances in long-read assemblers [51], TELSeq may help to improve inference of bacterial phylogenies and potential HGT events within and across host and environmental metagenomes.

### Recent antibiotic exposures may increase MGE-colocalized ARGs

Our primary experimental approach focused on samples with presumed low ARG density as a means to stress test the ability of TELSeq to recover targets with very low abundance, which it was able to do. We then applied TELSeq to a sample obtained from a context of recent therapeutic antibiotic exposure, under the hypothesis that this sample may have more ARG-MGE colocalizations [52, 53]. Indeed, within this ARG-dense sample, we not only observed a higher proportion of on-target reads and ARG richness than the low-density ARG samples (Fig. 3), but also many more ARGs flanked by diverse MGEs (Fig. 4 and Table 1). These results suggest that recent antibiotic exposures may create detectable ARG-MGE signals in metagenomic data. For example, the highest-priority, critically important extended-spectrum β-lactamase (ESBL) *bla*_CTX-M_ gene was flanked by *YfdP* phage integration maintenance proteins, plasmid-associated Tn3 resolvases, and enterobacterial IS1 family ISs (Table 1 and Fig. 4). The rapid and worldwide dissemination of *bla*_CTX-M_ has been termed a pandemic driven by the transfer of this ARG from commensal to pathogenic bacteria via MGEs [21, 54]. While plasmids have fueled the extensive dissemination of *bla*_CTX-M_ ARGs across distant taxa, TEs, ISs, and phage elements are most critical in the maintenance of mobility and expression of resistance [55]. Our ability to detect and robustly contextualize the mobility profile of the low-abundance *bla*_CTX-M_ from a complex metagenomic sample demonstrates the fundamental advancement offered by TELSeq over existing metagenomic approaches. This advancement may also allow improved detection of recent ARG transfer events from commensals to pathogens, which has been a major methodological gap in the field of microbial ecology.

### The efficiency of TELSeq depends on the diversity and abundance of target DNA

Without enrichment, we observed that <1% of the metagenomic data comprised ARG sequences (Supp Table 1), which corresponds well with previous estimates of 0.1% and 0.001% for soil and fecal metagenomes, respectively [56, 57]. Despite the fact that the probes were designed for use with short-read library preparation protocols, we found that TELSeq achieved on-target rates similar to those achieved by enriched short-read approaches [8, 9, 16], suggesting that the probes can successfully enrich longer fragments of DNA. The upper limit of capturable fragment length is unknown, although our TELSeq data included very long ARG-containing reads (>40,000bp), indicating that the probes can capture very long pieces of DNA. This was observed, despite the fact that the SureSelect system is commercialized with the intent to produce paired-end libraries for short-read (~150 bp) Illumina sequencing. However, most TELSeq reads were within the range of 1–5kbp, perhaps indicating that successful probe binding events tend to occur with DNA fragments of this length. Alternatively, the read length distribution in our dataset could be a function of the in situ DNA fragment lengths in the samples themselves, as previous non-enriched long-read metagenomic datasets obtained from feces reported similar read length distributions [58–60]. Further work should attempt to more closely quantify the probe binding and amplification capacity for different fragment lengths, with the goal of optimizing capture efficiency and read length, i.e., information value.

As with previous reports, we found a large difference in the proportion of on-target sequence data across sample types, and less so between replicates within each sample (Supp Table 1). While the between-replicate variability was significantly associated with differences in sequencing depth (Type III ANOVA *P* = 0.005), the much larger between-sample variability suggests that the ARG composition of the original sample likely exerts a large influence over the final efficiency of TELSeq. Previous studies employing short-read sequencing demonstrate a similar positive relationship between sequencing depth and resistome richness [5, 61]. However, the relationship between depth of sequencing and additive information gain for resistome profiles obtained by TELSeq requires systematic evaluation. Our early findings for samples with lower sequencing depth motivates further refinement of the requisite depth parameters to saturate the full range of ARG representation in an unbiased manner using target-enriched sequencing methods. Based on comparisons of SOIL with FMT and −Abx, we expect that samples possessing a diverse resistome with many low-abundance ARGs are likely to experience the biggest performance gains, in terms of both on-target rate (Fig. 3, Supp Table 1) and increases in detectable resistome richness and diversity (Fig. 2, Supp Table 2). This may be due to the ability of TELSeq to capture and amplify very low-abundance targets. Other factors could also be driving the performance differences between the sample types, including intrinsic microbiome-level differences such as biomass and underlying taxonomic composition, as well as extrinsic variables such as the biophysical characteristics of the sample matrix including presence of inhibitors [62]. These same physicochemical properties may also have caused the wide range of sequencing depth that we observed in this study, despite our attempts to achieve relatively even sequencing coverage for each replicate. Future work should investigate the relationship between sample properties and technical (i.e., workflow) factors, and how these influence both sequencing depth and on-target performance.

### The relative proportion of on- and off-target DNA influences TELSeq performance

Analysis of mock community sequence data revealed that TELSeq performance varied non-monotonically with the relative abundance of each organism in the sample (Fig. 6 and Supp Table 3). For example, *L. monocytogenes* was the most abundant genome in the mock community at 98 % of total gDNA, but because this genome did not contain many ARGs, it received a relatively small proportion of probes (Fig. 6). Given this, we would expect very low coverage of the *L. monocytogenes* genome by TELSeq reads overall, but relatively high coverage in the targeted regions. While we did observe 100% sensitivity for *L. monocytogenes*, we also observed a high proportion of off-target coverage of the *L. monocytogenes* genome by TELSeq reads, resulting in relatively low specificity (Fig. 5 and Supp Table 3). This discrepancy was likely caused by the very high abundance of *L. monocytogenes* DNA in the mock community and subsequent stoichiometric probe binding to non-target regions of the genome. This explanation was also supported by the fact that *L. monocytogenes* was the only genome for which TELSeq produced contiguous sequence coverage across the entire genome length (Fig. 6). Conversely, genomes with very low abundance in the mock community exhibited relatively high specificity but low sensitivity, as was the case with *S. aureus*, the lowest-abundance genome in the mock community at 89 × 10^−6^ % of total gDNA. The results for *S. aureus* reflect an estimate of the limit of detection of TELSeq given the sequencing depth used in this study. It is noteworthy that ultra-deep non-enriched long-read sequencing also failed to achieve robust coverage of *S. aureus*, as reported in [34] and demonstrated in Fig. 6. Taken together, TELSeq’s performance on a well-defined mock community suggests the existence of a limit to both sequencing sensitivity (i.e., as seen for low-abundance organisms such as *S. aureus*), as well as sequencing specificity (i.e., as seen for high-abundance organisms such as *L. monocytogenes*).

Overall, TELSeq achieved the most optimal balance of sensitivity and specificity for organisms with moderate abundance and a high proportion of probe coverage, as seen for *S. enterica* and *E. coli* (Figs. 5a, 6 and Supp Table 3). Future work should include thorough evaluation of the interaction between target relative abundance and on- and off-target binding rates for TELSeq probes.

TELSeq’s ability to recover ARGs was generally robust down to the relative abundance represented by *E. faecalis* and *S. aureus*, i.e., 8.9 × 10^−4^ and 8.9 × 10^−5^, respectively (Supp Table 3). The exception to this was the performance results for *P. aeruginosa*, which deviated substantially from the overall trend of high sensitivity and deep coverage for moderate- and high-abundance organisms (Supp Table 3 and Fig. 5a). *P. aeruginosa* is known for its recalcitrance to DNA extraction protocols [63] and very high G+C content, with a genome-wide mean of 67% [64]. These two factors likely led to the aberrant TELSeq performance results for *P. aeruginosa*, and future studies that utilize TELSeq should consider some of the more recent developments in extraction protocols for metagenomic samples, such as the three-peak protocol [34] or modified commercial protocols [65].

## Conclusion

TELSeq fills a critical gap in the field of microbial ecology by enabling highly sensitive detection of low-abundance genes from metagenomic samples, including detailed information about their immediate genomic context. This capability is particularly germane to advancing scientific understanding of HGT and AMR, which to-date has relied on in vitro donor-recipient conjugation experiments or in silico bioinformatic and statistical inference of historical HGT events. TELSeq offers a new approach by allowing us to observe MGE-ARG pairings across the entire metagenome, without the need for statistical inference. While we demonstrated TELSeq’s benefits using ARGs as probe targets, we note that the TELSeq workflow could easily be adapted for use with other targets, including MGEs, virulence factors, viruses, and pathogens. The potential applications of TELSeq are thus extensive, with likely uses in clinical decision-making, diagnostics, public health and food safety surveillance, and mining of metagenomes for novel functions. Further work is needed to support reproducible, consistent, and highly validated TELSeq-based workflows, including appropriate quantitative approaches to draw microbiome-level inferences from enriched sequence data, and more fine-tuned control of enrichment efficiency and sequencing depth variability across technical replicates and diverse sample types.

## Materials and methods

### Probe design

The probe set used in this work has been previously described [8]. Briefly, the MEGARes v1.0, BacMet, and Virulence Factor databases were combined to generate a non-redundant list of 5557 ARGs and associated nucleotide sequences known to confer resistance to antibiotics, metals, and biocides. Each sequence in this list was tiled at 1× coverage with a 120-nt window to generate 100% gene fraction coverage for every accession. The final probe set contained 31,250 unique probes (Agilent Technologies, ELID number 0792071), which was the set used in this work. Probes were manufactured by Agilent (Santa Clara, CA, USA).

### Sample collection and storage

A fresh fecal sample was collected from a healthy human donor to the Fecal Microbiota Transplant (FMT) program at the University of Minnesota School of Medicine (“FMT” sample). Enrollment of the donor was done according to strict inclusion and exclusion criteria previously described [66], and following the Investigational New Drug Application 15071. Exclusion criteria constituted factors identified on medical record history and physical exam, including metabolic, autoimmune, and chronic pain disorders, history of gastrointestinal disease or surgery, allergies, neurologic or psychiatric disorders, or use of antimicrobial therapy. Previous stool samples collected from this donor had tested negative for viral, parasitic (including *Giardia* and *Cryptosporidium*), and specific culturable vancomycin-resistant *Enterococci*, methicillin-resistant *Staphylococcus aureus*, carbapenem-resistant *Enterobacteriaceae*, *Escherichia coli* O157:H7, *Salmonella* spp., *Shigella* spp*.*, and *Yersinia* spp pathogens. The fecal sample used in the current study was collected in a single-use toilet hat and was allotted into multiple 50-mL conical polypropylene tubes and immediately transferred to a −80 °C freezer for storage after 30 min of initial collection. All donor-specific activities were approved by the University of Minnesota Institutional Review Board.

To obtain a sample from an animal with recent antibiotic exposure, approximately 250 g of fresh fecal material was collected per rectum from a periparturient Holstein Friesian dairy cow during follow-up medical examination for metritis (“+Abx” sample). One week prior to fecal collection, the animal received a two-course, 72-h treatment with 6.6 mg/kg of injectable ceftiofur crystalline-free acid, a third-generation cephalosporin antibiotic, following label instructions. Additionally, on the same day, approximately 250 g of fresh fecal material was collected per rectum from another periparturient Holstein Friesian dairy cow deemed systemically healthy during a routine medical check of herd-level metritis and metabolic disorders (“−Abx” sample). While this healthy cow was part of the same herd as the cow with metritis, she was kept in a different freestall and had not been previously exposed to systemic antibiotics. Aside from these specific differences, both cows were subject to the same management and farm conditions. Each fecal sample (+Abx and −Abx) was placed in a 50-mL conical tube and immediately placed on ice. Samples were transferred to a −80 °C freezer for storage within 2 h of collection. All cattle handling procedures and sample collection were performed by veterinarians in accordance with the University of Minnesota Institutional Animal Care and Use Committee (IACUC).

The soil sample (“SOIL”) was collected from a strip of un-utilized prairie with perennial grasses and forbs in Mower County, Minnesota, USA, at a depth of 0–20 in. into the soil column. Samples were composited from three points within 10 m and stored at 4 °C during physical and chemical characterization, and a subsample was transferred to −80 °C prior to gDNA extraction.

#### Commercial mock community standard

For purposes of method validation, a ZymoBIOMICS^TM^ microbial community standard (CSII) was obtained (Zymo Research Corporation, Irvine, CA, USA. Product D6310, Lot#: ZRC190842), containing ~1.5 × 10^9^ cells / mL of 10 microbial species (8 bacterial and 2 fungi), in log-distributed abundance ranging from 89.1% (*Listeria monocytogenes*) to 89 × 10^−6^ % (*Staphylococcus aureus*) (“MOCK” sample). Cells were suspended in DNA/RNA Shield reagent by manufacturer and were stored frozen upon arrival (−80 °C).

### gDNA extraction

Prior to extraction, 50 g portions of the +Abx, −Abx, and FMT fecal samples and a 250-g portion of the soil matrix were thawed at room temperature. Following a 5-min manual homogenization, the samples was disbursed in 0.25-g aliquots into PowerBead Pro tubes containing zirconium beads and 800 μl lysis buffer for extraction using the Qiagen DNEasy Powersoil Pro kit (Qiagen, Hilden, Germany, Lot# 163044275). Molecular-grade sterile water was placed into 4 randomly selected tubes to serve as negative controls (extraction blanks). After 6 s of vortexer-mediated homogenization, samples were placed on a 115-V Mini-Beadbeater-96 (BioSpec Products, Bartlesville, OK, USA) for mechanical lysis. Sample bead beating proceeded at 2400 rpm for 30 s for a total of 3 rounds with a 2-min pause on ice between each round, to prevent overheating. The remainder of the extraction procedure followed the PowerSoil Pro recommendations with inhibitor removal steps, and the final 50 μl of eluted gDNA was stored at −20 °C.

For the ZymoBIOMICS mock community, all extraction procedures including additional pre-extraction steps to ensure that the final gDNA maintained optimal fragment length and species representation after isolation from the native DNA/RNA shield storage solution, were followed according to the methods outlined by Nicholls et al. [34]. The standard was divided into ten 75-μl aliquots, each centrifuged at 8000×*g* for 3.5 min before removing and retaining the supernatant, which contained lysed Gram-negative species in the DNA/RNA Shield storage solution. The pellets were resuspended in 700 μl lysis buffer and were transferred to zirconium-containing PowerBead Pro tubes of the Qiagen DNEasy Powersoil Pro kit (Qiagen, Hilden, Germany, Lot# 163044275). Molecular-grade sterile water was added to 4 randomly selected tubes to serve as negative controls (extraction blanks). After 6 s of vortexer-mediated homogenization, samples were placed on a 115-V Mini-Beadbeater-96 (BioSpec Products, Bartlesville, OK, USA) for mechanical lysis. Sample bead beating proceeded at 2400 rpm for 5 min for a total of 4 rounds with a 5-min pause on ice between each round. The resulting ~500μl of supernatant was retained and recombined with the supernatant retained earlier, and the samples were subjected to the remaining recommended procedures of the PowerSoil Pro kit, including inhibitor removal steps. The final 50μl of eluted gDNA was stored at −20 °C.

Quantitation of all isolated gDNA was performed using the Qubit 4 Fluorometer (Invitrogen, Carlsbad, CA, USA) using the dsDNA high-sensitivity assay kit. Electrophoretic assessment of DNA quality was performed using a genomic screen tape and reagents on a 4200 TapeStation (Agilent, Santa Clara, CA, USA). All extraction blanks contained no quantifiable gDNA and therefore were not carried forward into library preparation, targeted enrichment, and sequencing. All sample processing occurred in a Class II Biological Safety Cabinet and was performed by a single laboratory technician following standard decontamination practices using 70% ethanol and irradiation.

### TELSeq library preparation and enrichment

#### DNA fragmentation and size selection

The SureSelect^XT^ V1 system incorporates library preparation and targeted enrichment procedures and is optimized for 200 ng–3 μg of gDNA input to generate 150–200-bp insert fragments for Illumina sequencing (100–500 bp read length). In this study, we scaled the SureSelect^XT^ V1 system to produce enriched libraries for PacBio sequencing (>50kb read length) which require comparably longer gDNA inserts (>1000bp). Generating libraries with longer insert ranges is partially dependent on sample DNA content [67]. To determine whether gDNA fragment size impacted the enrichment dynamics of TELSeq, we initially tested a range of insert sizes using the +Abx and MOCK samples. Technical triplicate libraries for +Abx and MOCK were created as follows: 3μg gDNA to achieve a ~2-kb insert range; 4μg gDNA from two equimolar pools to achieve a ~5 kb insert range; and 6μg gDNA from three equimolar pools to achieve an ~8 kb insert range. For the 2 or 5 kb replicates, the requisite input gDNA was resuspended in 1× TE buffer (pH 8.0) to a final volume of 200μl in COVARIS miniTUBEs (COVARIS Inc, Woburn, MA, USA) and mechanically fragmented using an M220 COVARIS focused ultrasonicator (COVARIS Inc, Woburn, MA, USA) with the following settings: for 2 kb replicates, peak power-6W, duty factor-20%, cycles/burst-900, 800 s, 4 °C; for 5 kb replicates, Peak power 6W, duty factor 20%, cycles/burst 900, 500 s, 4 °C. For 8-kb replicates, the appropriate gDNA was resuspended in 1× TE buffer (pH 8.0) to a final volume of 150μl in a COVARIS g-TUBE (COVARIS Inc, Woburn, MA, USA). g-TUBEs were centrifuged at 7200 rpm for 1 min on an Eppendorf 5424 rotor (Eppendorf AG, Hamburg, Germany).

For all other samples (i.e., −Abx, FMT and SOIL), technical triplicate libraries were prepared using two equimolar gDNA pools of each sample type to achieve 4μg gDNA input for a ~5-kb insert range. Fragmentation parameters for these libraries followed as described for the 5 kb protocol above. After fragmentation, all libraries were then subjected to 0.8 (vol/vol) AMPure XP bead purification (Agencourt Biosciences Corp., Beverly, MA, USA) and electrophoretic verification using an Agilent TapeStation 4200 (Agilent, Santa Clara, CA, USA), see Additional file 1 for gDNA quality results of individual pre-pooled aliquots for all sample types.

Sheared and purified test libraries were all subjected to insert size selection to achieve either 2, 5, or 8–10 kb for +Abx and MOCK, or >5 kb insert size for replicates of −Abx, FMT and SOIL, using a 0.75% agarose gel DF cassette (cat no. BLF7510) with S1 marker (Sage Science Inc., Beverly, MA, USA) on a BluePippin pulse-field electrophoretic size selector (Sage Science Inc., Beverly, MA, USA). All library samples were eluted using 10μl of supplied elution buffer for a given runtime, to obtain a final volume of 40μl.

#### Targeted hybridization and capture

Following fragmentation, the SureSelect^XT^ V1 protocol for 200ng of input was used, with numerous modifications, as follows. The entire gDNA content of each sample (diluted in 40μl) was used beginning with the DNA end-repair step. Upon completion of end-repair and dA-tailing procedures, AMPure XP bead purification using a 0.8 (vol/vol) bead:sample mixture was performed. In preparation for adapter ligation, an adapter oligonucleotide mixture was first prepared at a 1:4 ratio, and this was added to the master mix immediately before aliquoting into each sample. Pre-capture PCR amplification in all samples was performed on the entirety of each library with a modified thermocycler program: 2 min at 96 °C, 11 cycles of the following: 20 s at 96 °C, 30 s at 65 °C and 2 min/5 min/6 min at 72 °C for the 2 kb, 5kb, and 8kb +Abx and MOCK samples, respectively. For the −Abx, FMT, and SOIL samples, the 5-min hold time was performed at 72 °C. Samples were then maintained an additional 10 min at 72 °C, and ramped down to 4°C. Pre-capture amplified libraries were subjected to 0.8 (vol/vol) AMPure XP bead purification (Agencourt Biosciences Corp., Beverly, MA, USA) and electrophoretic verification using an Agilent TapeStation 4200 (Agilent, Santa Clara, CA, USA).

Following lyophilization and addition of hybridization buffers and reagents, hybridization was performed on ~600 ng of amplified gDNA libraries with the addition of equimolar quantities of custom-designed biotinylated probes to each library, along with 10% RNase block solution. Incubation proceeded for 16 h at 65 °C with the heated lid set to 75 °C to minimize evaporation and maintain the integrity of longer fragments. Subsequent capture steps were performed using per-protocol conditioned MyONE streptavidin T1 beads (Invitrogen Co, Waltham, MA, USA). Hybridization was facilitated by placing samples on a plate mixer (1200rpm) for 5 min at room temperature. The mixer was paused and incubation proceeded for an additional 55 min at room temperature, while manually mixing (i.e., pipetting up and down 10 times) every 7 min in order to maintain capture and amplification of larger fragments for long-read library preparation. Following hybridization steps, we performed capture steps using per-protocol reagents and sample-to-reagent ratios, as well as temperatures and durations. We performed three additional bead washing cycles to optimize the retention of probe-mediated captured DNA fragments.

For post-capture indexing and amplification steps, the entire volume of each sample was used, rather than half as recommended by the manufacturer’s protocol. After combining samples with the appropriate volume of each indexing and amplification reagent, the following modified thermocycler program was used: 2-min hold at 96 °C followed by 18 cycles of ramping between 96 °C (20 s), 65 °C (30 s), and 72 °C (2 kb: 2 min; 5 kb: 5 min; or 8 kb: 6 min). For the −Abx, FMT, and SOIL samples, the 5-min hold time at 72 °C was used. Samples were held for an additional 72 °C for 10 min. Indexed and amplified captured libraries were subjected to 0.8 (vol/vol) AMPure XP bead purification (Agencourt Biosciences Corp., Beverly, MA, USA) and electrophoretic verification using an Agilent TapeStation 4200 (Agilent, Santa Clara, CA, USA).

Subsequent to enrichment steps, purity of all library gDNA was measured using a NanoDrop spectrophotometer (Thermo Fisher Scientific, Waltham, MA, USA). Samples having absorbance ratios at 260/280 nm and 260/230 nm of >1.8 were selected for sequencer-specific library preparation. TELSeq and PacBio samples were subjected to multiplexed CCS library creation, using the Pacific Biosciences template preparation protocol for metagenomic samples without additional shearing and amplification. Briefly, the PacBio SMRTbell Express Template Preparation Kit (v 2.0) was used to perform damage repair, end-repair, and 5′ phosphorylation. After ligation of PacBio hairpin loop adapters with overhang barcoding (8A Barcoded Overhang Adapter Kit: 101-628-400) to repaired and end-phosphorylated fragments in each sample, the resulting SMRTbell templates were cleaned using nuclease treatment followed by clean-up with 0.45× AMPure PB beads. For short-read (SR) metagenomic sequencing, Illumina’s Nextera DNA Flex Library Preparation Kit was used, following the manufacturer’s protocols (Illumina Inc., San Diego, CA, USA).

### Circular Consensus Sequencing (CCS)

All prepared SMRTbell template TELSEq and un-targeted long-read (i.e., PB) libraries were evenly pooled and sequenced using two serial runs of equal sequencing depth on a Pacific Biosciences Sequel 6.0 system (Pacific Biosciences, Menlo Park, CA, USA) using two Sequel SMRT cells (1M with 3.0 chemistry). A 20-h movie runtime was used for each cell. PacBio CCS reads (minPassess= 3; MinAccuracy= 90%) were generated using SMRT Link v 7.0.

### Short-read (SR) metagenomic sequencing

Replicates of −Abx, FMT, and SOIL samples were subjected to paired-end short-read sequencing (2 × 150bp) using a single S4 flow cell lane on an Illumina NovaSeq6000 platform (Illumina Inc., San Diego, CA, USA).

### Previously generated MOCK community data

The ZymoBIOMICS^TM^ log-distributed microbial community standard (CSII) was previously subjected to metagenomic sequencing using deep and ultra-deep long-read Oxford Nanopore sequencing on the GridION and PromethION platforms, respectively [34]. The mock community reads were generated from 50-ng and 400-ng aliquots of the same lot used in the current study (lot# ZRC198042) on each of the two flow cells of the Oxford Nanopore Technologies (Oxford Science Park, Oxford, UK) GridION (FASTQ accession: ERR3152366) and PromethION (FASTQ accession: ERR3152367) sequencers. Reads were produced with an expected 5-fold throughput difference per flow cell (GridION: 3.67 M reads vs PromethION: 34.5 M reads). Additional metadata describing species-level genome references, relative distribution, sequencing yield, and coverage are described in Supp Table 4 and in [34]. These libraries served as benchmarks for the MOCK replicates generated using TELSeq.

### Bioinformatic analysis

#### Quality filtering and deduplication

The target enrichment protocol deploys PCR for amplifying DNA during adaptor-ligation and targeted site capture, potentially resulting in duplicated reads in the downstream sequence data at levels above what would be expected from non-enriched metagenomic data [68]. Therefore, CCS reads [69] for both the TELSeq and PB libraries for all sample types were subjected to a deduplication procedure using a similarity-based approach as follows. First, CCS reads were clustered based on length using sklearn.cluster.KMeans class using 200 clusters. Next, all CCS reads in one cluster were pairwise aligned with Blast-Like Alignment Tool (BLAT) [70]. Reads were considered duplicates if the span of all the hit/query high-scoring segment pairs (HSPs) were greater than or equal to 90% of the total hit/query length. Sets of duplicate reads were accumulated, and deduplicated FASTQ files were generated by randomly retaining a single read for each duplicated set from the original library FASTQ. For these analyses, the pysam Python module was used to parse SAM files, and the SeqIO and SearchIO modules from Biopython were used to parse FASTA/FASTQ files and PSL files, respectively. We found that it was impractical to apply a similar deduplication procedure to MOCK libraries generated by Oxford Nanopore sequencing of Nicholls et al. [34] in a reasonable timeframe, due to limitations in computing resources necessary to process the ultra-deep read depth (i.e., >14 CPU days were needed to process <1% of the PromethION data on a machine with 1TB of memory). Additionally, the CCS duplication rate in the PB libraries was minimal (i.e., <0.1%), which was expected given that no PCR-based amplification procedures were used in PB library creation. Since no amplification procedures were likewise used to generate the Nanopore libraries [34], we determined that it was not necessary to deduplicate the MOCK libraries generated on the GridION and PromethION. We note that this decision results in a more conservative comparison of TELSeq to the GridION and PromethION datasets. Differences in deduplicated read depth between sequencing platforms were evaluated using repeated-measures analysis of variance (ANOVA), with use of Levene’s test to evaluate heteroscedasticity and an alpha of 0.05 as the predetermined level of statistical significance.

#### Resistome and mobilome analysis

In order to identify ARGs from the TELSeq and PB approaches for all +Abx, −Abx, FMT, SOIL, and MOCK libraries, the deduplicated data were aligned to MEGARes v2.1 [71] for detection of the resisome and ACLAME v0.4 [72], ICEberg v2.0 [73], and PlasmidFinder v2.1 [74] for detection of the mobilome, using Minimap 2 [75] with the -ax flag set to map-pb. The GridION and PromethION mock community datasets were divided into several FASTQ files using fastqsplitter (https://kirill-kryukov.com/study/tools/fastq-splitter/, v0.1.2) and then aligned to MEGARes v2.1 using minimap2 with the -ax flag set to map-ont. The Sequence Alignment Map (SAM) files that resulted from alignment to the MEGARes and MGE databases were used to characterize the resistomes and mobilomes in CCS-type reads. Custom Python scripts were used to count the number of unique MEGARes classes, mechanisms, groups, and genes in each library. Next, the same script was used to count the number of unique MGE accessions by type, i.e., plasmids, integrative conjugative elements (ICE), prophages, and virulence factors. Note that regions of the reads that aligned to the MEGARes database were not considered during this step. Additionally, ISfinder [76], and ISbrowser [77] were used to parse hits for plasmid, prophage, and virulence accessions, to identify insertional sequence (IS) families and other transposable elements (TEs), using BLASTN (E-value = 1 × 10^−10^, minimal identity 80% over >80% of the query length). To reduce false positive detection of ARG and MGE accessions, we used a gene fraction cutoff (defined as the proportion of nucleotides within a given reference accession that are aligned by at least one sequenced read) of 80% and 50% for resistome and mobilome accessions, respectively, based on cut-offs established in previous work that reported resistome-mobilome colocalization analysis on metagenomic data [17, 78]. The counts of unique features and alignments per feature were used to describe the richness and diversity of the resistome and mobilome in each library. We evaluated the correlation between the predictor sequencing depth (i.e., number of raw TELSeq reads per replicate) and the dependent variable on-target rate (i.e., proportion of reads that aligned to ARGs out of all sequenced reads) and class-level ARG richness (i.e., number of unique ARG classes detected per replicate) using a linear mixed-effects model as implemented in the *lmer* function of *lme4* [79]. For both correlation analyses, sample type was specified as the random effect to account for non-independence of the technical replicates, and the number of sequence reads was log-transformed to meet model assumptions. Statistical significance using an alpha of 0.05 was evaluated via a Type III analysis of variance with Satterthwaite approximation, as implemented in the *anova* function in *lme4.* Ordination of the resistome and microbiome count matrices was performed using non-metric multidimensional scaling of Hellinger-transformed counts, with subsequent testing of significant differences between sample type and sequencing platform using analysis of similarities (ANOSIM) and permutational multivariate analysis of variance (PERMANOVA), with an alpha of 0.05 as the predetermined level of statistical significance.

De-multiplexed metagenomic libraries obtained via the short-read sequencing approach were analyzed using the AMRPlusPlus [71]. After read trimming and quality filtering using TRIMMOMATIC [80], AmrPlusPlus identifies reads that align to the relevant host genome (+Abx and −Abx samples: *Bos taurus* [UMD3.1] reference genome; FMT, SOIL, and MOCK samples: *Homo sapiens* [hg19] reference genome) using Burrows-Wheeler Aligner (BWA) software [81], and filters host-aligned reads via SamTools [82] to create a set of non-host reads for subsequent characterization of the resistome and mobilome.

#### Identification of ARG-MGE colocalizations and cargo genes

To identify high-confidence colocalizations, we implemented a strict definition in which a single read had to contain both an ARG and an MGE at 80 and 50% gene fraction, respectively, again based on previously used cut-offs [17, 78]. We applied convergence restrictions as well as a regional 250 bp buffer zone around all identified ARGs to remove the potential for multiple alignments to both the ARG and MGEs within the same start:stop position on the read. This was done due to extensive sequence homology between multiple accessions in the ARG and MGE reference databases. In order to identify potential cargo genes on the reads that contained at least one ARG and MGE (i.e., colocalizations), we aligned each such read to the KEGG [83] gene database for prokaryotic organisms. For MOCK replicates, we used a subset of the KEGG database containing only the 10 organisms contained in the mock community. A cargo gene was considered present if the length of the alignment on the reference gene in KEGG covered at least 50% of the length of the entire gene.

The MEGARes alignment file, the three MGE database alignment files and the KEGG alignment file were parsed with a custom Python script to generate a CSV file in which each row contained the following colocalization information: read identifier, MEGARes accession, MGE database accession, and the KEGG accession, along with the position of the genes on the read. We note that each possible combination of ARG, MGE, and KEGG gene was considered and stored in the CSV file. Next, we defined colocalization distance as the distance between the end of the ARG alignment on the read and the start of the MGE alignment on the read, or vice versa. Two colocalizations were defined to be non-unique if their ARGs belonged to the same MEGARes group, if their MGEs had the same database accession, and if their colocalization distances were within 250 bp of each other. A custom Python script was used to group colocalizations into 250-bp intervals based on the order in which they were encountered going through the CSV of colocalizations. For example, if three colocalizations with the same MEGARes group and MGE accession were encountered with colocalization distances of 50, 150, and 350 bp, in that order, the first two colocalizations would be considered non-unique and the third would be considered unique. Additionally, analysis of colocalizations was restricted to only those colocalized ARGs that did not require additional confirmation of single-nucleotide polymorphisms (SNPs).

#### Benchmarking TELSeq using mock community genome coverage analysis

Each TELSeq and non-targeted mock community library was aligned to the reference genomes of all 10 microbes within the mock community using minimap2. For the TELSeq mock community libraries, a custom Python script was used to parse the resulting SAM file to determine the percentage of reads that aligned to each genome and to find the longest alignment from a TELSeq read to each genome. Another Python script was used to compare the coverage along each genome between the three TELSeq mock community libraries and the two non-targeted mock community libraries sequenced by high-depth (GridION) and ultra high-depth (PromethION) Nanopore libraries. For all TELSeq MOCK libraries, probe sensitivity (i.e., “on-target” rate) was quantified as the proportion of all probe-targeted bases present in each genome that received at least 1× TELSeq read coverage. *L. fermentum*, *C. neoformans*, and *S. cerevisiae* did not have any known ARGs in their genome and did not receive any probe-specific coverage; therefore, the on-target rates by TELSeq for these organisms were not calculable. Probe specificity of TELSeq (i.e., “off-target” rate) was quantified as the proportion of non-targeted bases that did not receive TELSeq read coverage. TELSeq sensitivity and the inverse specificity (土 95% CI) across all genomes was plotted against log-normalized genome relative abundance in the mock community using LOESS regression specification with minimum spanning of 0.2. We independently evaluated the correlation between the genome relative abundance and the dependent variables of either on-target or off-target rates using a linear mixed-effects model as implemented in the *lmer* function of *lme4* [79]. For both correlation analyses, genome type was specified as the random effect while shearing size among technical replicates was specified as a fixed effect. Statistical significance using an alpha of 0.05 was evaluated via a Type III analysis of variance with Satterthwaite approximation, as implemented in the *anova* function in *lme4.* We further explored off-target sequencing in TELSeq by profiling reads generated for *L. fermentum*, *C. neoformans*, and *S. cerevisiae*, all of which do not have any probe-aligned ARGs in their genomes and can therefore be considered as entirely off-target sequences. Resistome analysis of these off-target reads was conducted as previously described using the same pipeline parameters and defaults, specifying MEGARes v1.0 as the reference, as this version formed the basis for TELSeq probe design. Alignments were parsed to remove any ARG hits that required additional confirmation of single-nucleotide polymorphisms (SNPs).

## Supplementary Information

**Additional file 1.** Agilent TapeStation 4200 gDNA gel and electropherogram results. Output of gDNA size distribution (bp) on genomic tape runs of technical replicates used as input for TELSeq, PB, and Illumina library generation for either: (a) +Abx, Bovine fecal sample retrieved from a Holstein-Fresian dairy cow with recent antimicrobial drug exposure; (b) −Abx, Bovine fecal sample retrieved from a Holstein-Fresian dairy cow with no recent history of antimicrobial drug exposure reared under the same husbandry conditions as +Abx cow; (c) FMT, Fecal microbiota transplant sample submitted from a healthy U.S. human donor; (d) SOIL, Composite prairie soil sample collected from an undeveloped easement in Mower County, MN, USA ; and (e) MOCK, ZymoBIOMICS^TM^ microbial community standard composed of 8 prokaryotic and 2 eukaryotic microorganisms in logarithmic distribution (CSII) obtained from Zymo Research Corporation, Irvine, CA, USA, Product D6310, Lot#: ZRC190842.

**Additional file 2: Supplementary Figure 1.** Resistome beta-diversity*.* Non-metric multidimensional scaling ordination plots used to identify differences in resistome beta-diversity at the group level of ARG ontology, across sample types (a): +Abx (black), −Abx (red), FMT (green), and SOIL (blue), as well as according to sample type and sequencing approach (b): −Abx using TELSeq (green); −Abx using PacBio (pink); −Abx using Illumina (black); FMT using TELSeq (magenta); FMT using PacBio (cyan); FMT using Illumina (blue); SOIL using TELSeq (orange); SOIL using PacBio (gray); SOIL using Illumina (yellow). Ordinations were based on Euclidean distances derived from Hellinger-transformed alignments counts.

**Additional file 3: Supplementary Figure 2.** Mobilome abundance, by MGE type. Kernel density (y-axis) plots depicting the log_10_ relative abundance (x-axis) of sequence depth-normalized MGE accessions detected in −Abx, FMT, and SOIL samples by TELSeq, PacBio, and Illumina sequencing platforms. Mobile element data are shown for specific mobilome classes: ICE (purple); IS (chartreuse); plasmid (blue); prophage (green); TE (magenta); Virus (sky blue).

**Additional file 4: Supplementary Figure 3.** Sequencing coverage for two eukaryotic constituents of MOCK. Sequencing coverage achieved by GridION is indicated in gray, while TELSeq coverage is indicated in tan (2kb library), blue (5kb library) and green (8 kb library). Zoomed-in subsets from select loci of each genome are included for visualization purposes. Log_10_ relative abundance of each genome in MOCK is displayed beneath the genome name.

**Additional file 5: Supplementary Table 1.** Sequencing and on-target statistics by sample type and platform.

**Additional file 6: Supplementary Table 2.** Summary of ARG and MGE relative composition and richness identified by TELSeq, PacBio, and Illumina reads generated from +Abx, −Abx, FMT, SOIL and MOCK replicates.

**Additional file 7: Supplementary Table 3.** TELSeq sequencing sensitivity, sequencing specificity, and excess read depth, by genome and MOCK replicate (2kb, 5kb and 8kb).

**Additional file 8 Supplementary Table 4.** Summary of species distribution and previously generated sequencing results for the ZymoBIOMICS^TM^ mock microbial community standard (CSII) in logarithmic abundance.

## Data Availability

Custom scripts used to perform CCS read analysis as well as resistome, mobilome, and cargo gene analysis, colocalization analysis and annotation, as well as coverage mapping, are freely available at https://github.com/marco-oliva/argmobrich_analysis. All raw sequencing data and sequence metadata generated in this study have been submitted to the NCBI Sequence Read Archive (SRA) under BioProject accession number PRJNA751055 (https://www.ncbi.nlm.nih.gov/bioproject/PRJNA751055). Biosamples were created for all TELSeq libraries utilizing the MIMS v5.0 reporting guidelines developed by the Genomic Standards Consortium (GSC). All analyzed data during this study are included in this published article and its supplementary files.

## Abbreviations

TELSeq: Target-enriched long-read sequencing
MGE: Mobile genetic element
AMR: Antimicrobial resistance
ARG: Antimicrobial resistance gene
Hi-C: High-throughput chromatin capture
ICE: Integrative and conjugative element
PacBio: Pacific Biosciences of California, Inc
CCS: Circular consensus sequencing
BLAST: Basic local alignment search tool
IS: Insertional sequence
HGT: Horizontal gene transfer
MDR: Multidrug resistance
VRE: Vancomycin-resistant *Enterococci*
TE: Transposable element
ESBL: Extended-spectrum β-lactamase
UMI: Unique molecular index
CSII: Community standard II (ZymoBIOMICS microbial community)
BLAT: BLAST-like alignment tool
HSP: High-scoring segment pair
SRA: Sequence read archive
GSC: Genomic Standards Consortium
NIH: National Institutes of Health
NIAID: National Institute of Allergy and Infectious Disease
UMGC: University of Minnesota Genomics Center
