# Supplementary material for: Target-enriched long-read sequencing (TELSeq) contextualizes antimicrobial resistance genes in metagenomes
Source: Microbiome. 2022 Nov 2;10:185. doi: 10.1186/s40168-022-01368-y (PMC9628182; doi:10.1186/s40168-022-01368-y)
Supplement: Supplementary file 2 — Additional file 1. Agilent TapeStation 4200 gDNA gel and electropherogram results. Output of gDNA size distribution (bp) on genomic tape runs of technical replicates used as input for TELSeq, PB, and Illumina library generation for either: (a) +Abx, Bovine fecal sample retrieved from a Holstein-Fresian dairy cow with recent antimicrobial drug exposure; (b) −Abx, Bovine fecal sample retrieved from a Holstein-Fresian dairy cow with no recent history of antimicrobial drug exposure reared under the same husbandry conditions as +Abx cow; (c) FMT, Fecal microbiota transplant sample submitted from a healthy U.S. human donor; (d) SOIL, Composite prairie soil sample collected from an undeveloped easement in Mower County, MN, USA ; and (e) MOCK, ZymoBIOMICSTM microbial community standard composed of 8 prokaryotic and 2 eukaryotic microorganisms in logarithmic distribution (CSII) obtained from Zymo Research Corporation, Irvine, CA, USA, Product D6310, Lot#: ZRC190842. [file 40168_2022_1368_MOESM1_ESM.pdf]

Additional Data File 1

Target-enriched long-read sequencing (TELSeq) contextualizes antimicrobial resistance risk in metagenomes

Ilya B. Slizovskiy[†1], Marco Oliva[†2], Jonathen K. Settle[2], Lidiya V. Zyskina[3], Mattia Prosperi[4], Christina Boucher[2], Noelle R. Noyes[1]*

Affiliations:
1. Food-Centric Corridor, Infectious Disease Laboratory, Department of Veterinary Population Medicine, College of Veterinary Medicine, University of Minnesota, St. Paul, MN, USA

2. Department of Computer and Information Science and Engineering, Herbert Wertheim College of Engineering, University of Florida, Gainesville, FL, USA

3. Program in Human-Computer Interaction, College of Information Studies, University of Maryland, College Park, MD, USA

4. Data Intelligence Systems Lab, Department of Epidemiology, College of Public Health and Health Professions and College of Medicine, University of Florida, Gainesville, FL, USA

[†]Designated as first authors with equal contribution

****To whom correspondence shall be addressed: Dr. Noelle R. Noyes: nnoyes@umn.edu***

Additional File 1. Agilent TapeStation 4200 gDNA gel and electropherogram results. Output of gDNA size distribution (bp) on genomic tape runs of technical replicates used as input for TELSeq, PB, and Illumina library generation for either: (**a**) +Abx, Bovine fecal sample retrieved from a Holstein-Fresian dairy cow with recent antimicrobial drug exposure; (**b**) -Abx, Bovine fecal sample retrieved from a Holstein-Fresian dairy cow with no recent history of antimicrobial drug exposure reared under the same husbandry conditions as +Abx cow; (**c**) FMT, Fecal microbiota transplant sample submitted from a healthy U.S. human donor; (**d**) SOIL, Composite prairie soil sample collected from an undeveloped easement in Mower County, MN, USA ; and (**e**) MOCK, ZymoBIOMICS™ microbial community standard composed of 8 prokaryotic and 2 eukaryotic microorganisms in logarithmic distribution (CSII) obtained from Zymo Research Corporation, Irvine, CA, USA, Product D6310, Lot#: ZRC190842.

a. + Abx

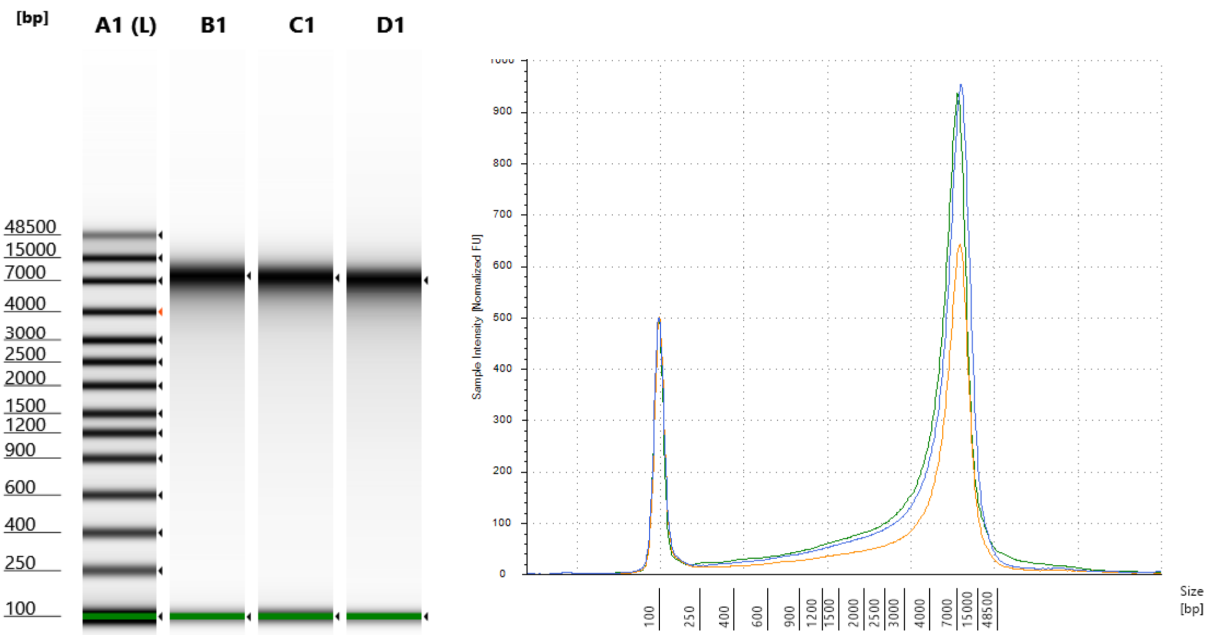
[bp]
A1 (L)
B1
C1
D1
48500
15000
7000
4000
3000
2500
2000
1500
1200
900
600
400
250
100
Sample Intensity [Normalized FU]
900
800
700
600
500
400
300
200
100
0
Size [bp]
100
250
400
600
900
1200
1500
2000
2500
3000
4000
7000
15000
48500

b. - Abx

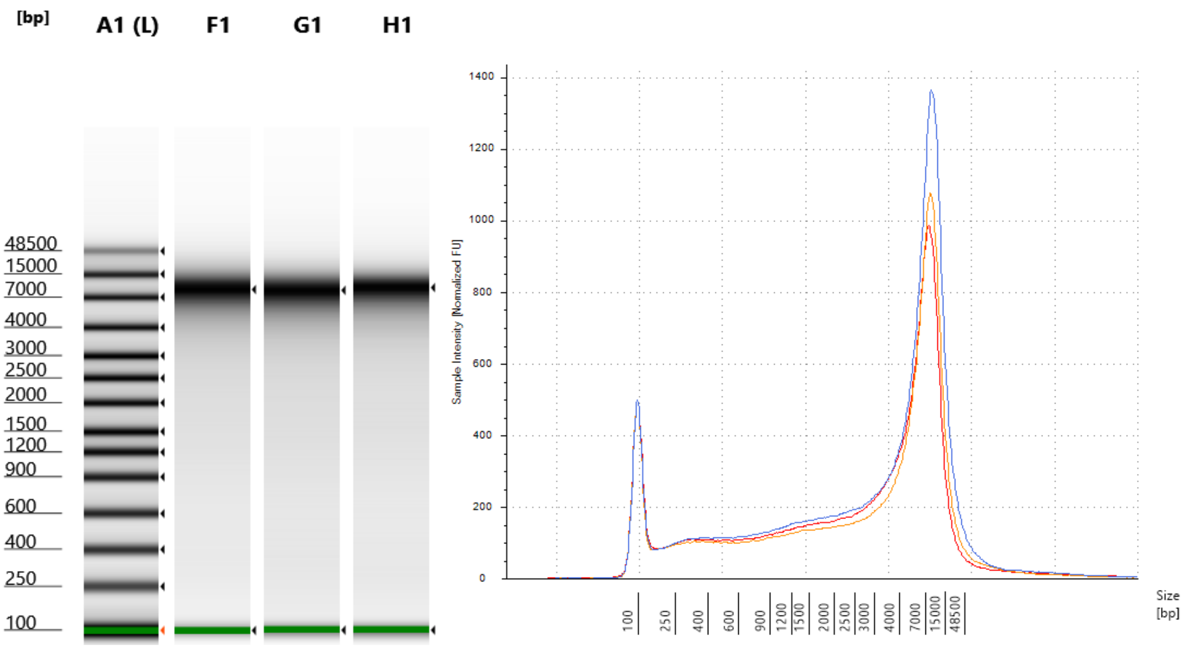
[bp]
A1 (L)
F1
G1
H1
48500
15000
7000
4000
3000
2500
2000
1500
1200
900
600
400
250
100
Sample Intensity [Normalized FU]
1400
1200
1000
800
600
400
200
0
Size [bp]
100
250
400
600
900
1200
1500
2000
2500
3000
4000
7000
15000
48500

c. FMT

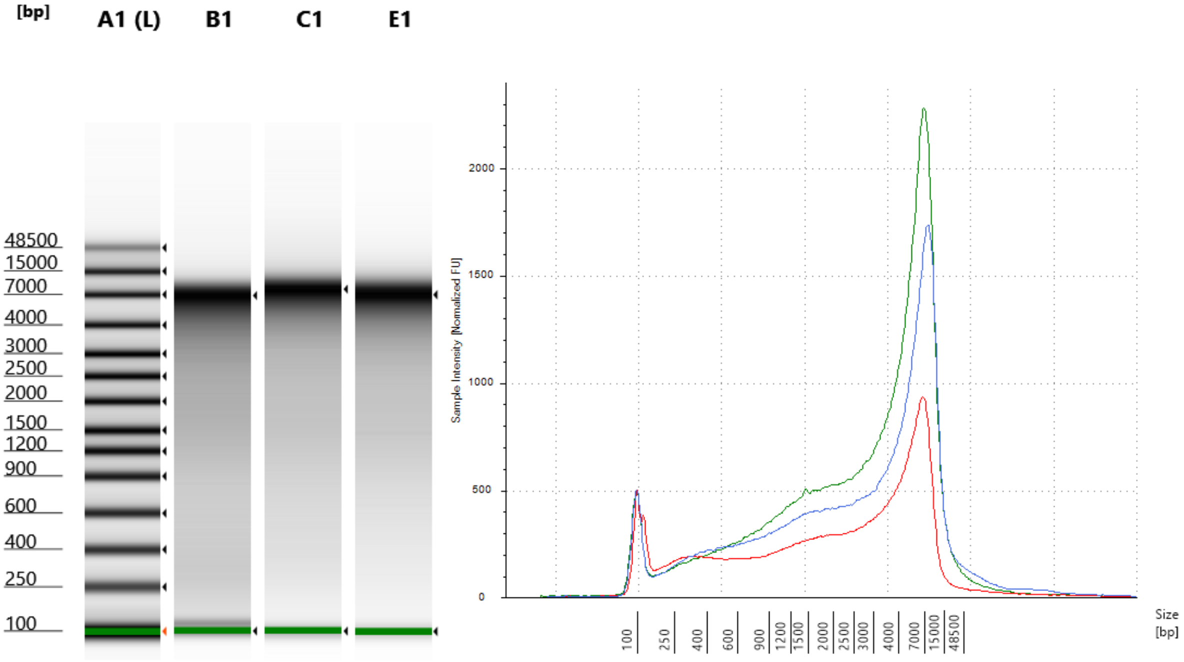

[bp]
A1 (L)
B1
C1
E1
48500
15000
7000
4000
3000
2500
2000
1500
1200
900
600
400
250
100
Sample Intensity [Normalized FU]
2000
1500
1000
500
0
100
250
400
600
900
1200
1500
2000
2500
3000
4000
7000
15000
48500
Size
[bp]

d. SOIL

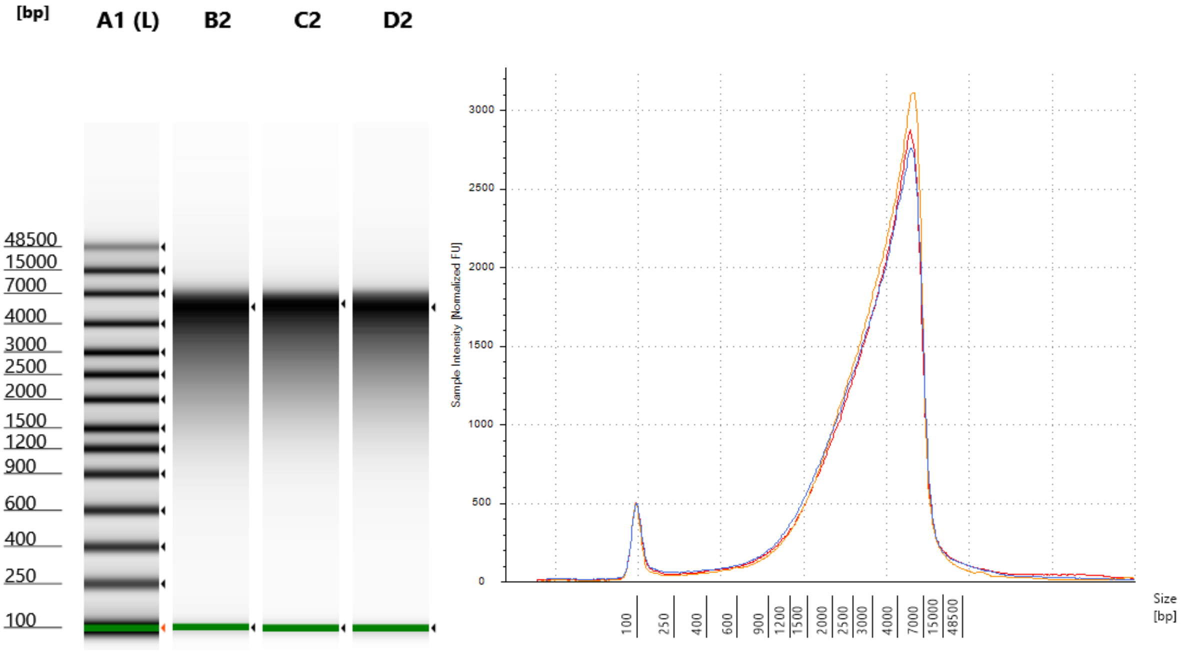

[bp]
A1 (L)
B2
C2
D2
48500
15000
7000
4000
3000
2500
2000
1500
1200
900
600
400
250
100
Sample Intensity [Normalized FU]
3000
2500
2000
1500
1000
500
0
100
250
400
600
900
1200
1500
2000
2500
3000
4000
7000
15000
48500
Size
[bp]

e. MOCK

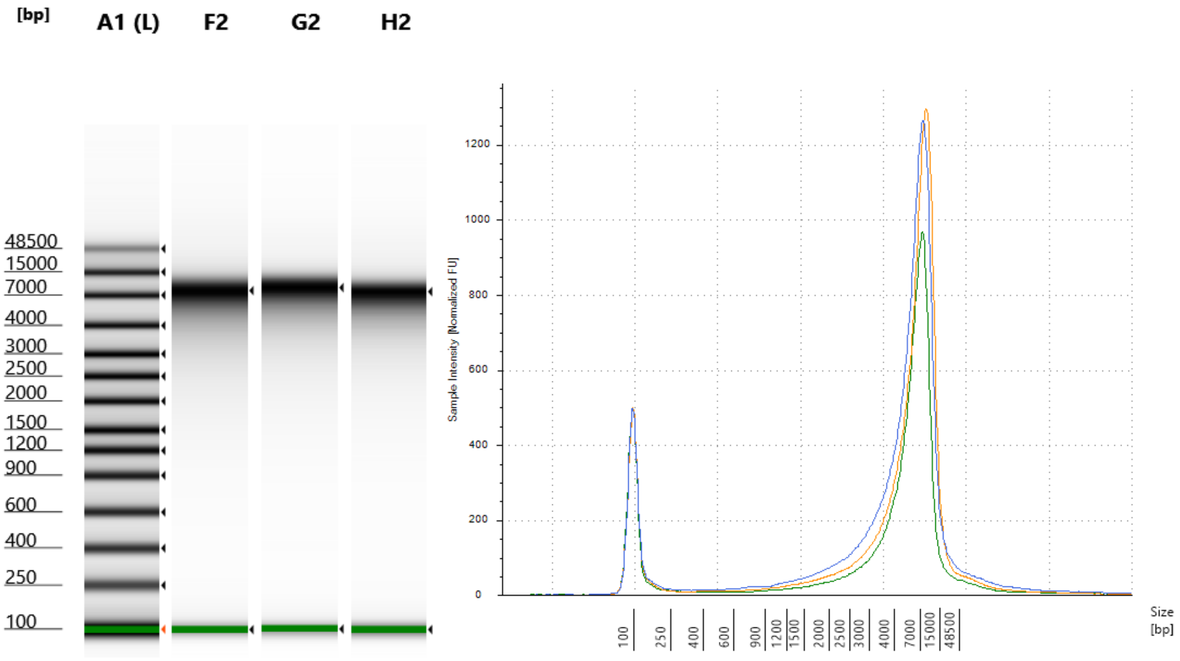
[bp]
A1 (L)
F2
G2
H2
48500
15000
7000
4000
3000
2500
2000
1500
1200
900
600
400
250
100
Sample Intensity [Normalized FU]
1200
1000
800
600
400
200
0
Size [bp]
100
250
400
600
900
1200
1500
2000
2500
3000
4000
7000
15000
48500
