# Supplementary material for: Target-enriched long-read sequencing (TELSeq) contextualizes antimicrobial resistance genes in metagenomes
Source: Microbiome. 2022 Nov 2;10:185. doi: 10.1186/s40168-022-01368-y (PMC9628182; doi:10.1186/s40168-022-01368-y)
Supplement: Supplementary file 6 — Additional file 5: Supplementary Table 1. Sequencing and on-target statistics by sample type and platform. [file 40168_2022_1368_MOESM5_ESM.docx]

**Supplementary Table 1.** Sequencing and on-target statistics by sample type and platform.

| **Sample** | **Sequencing platform** | **Yield (Gb)** | **Raw reads** | **De-duplicated reads** | **Duplication**  **(%)** | **ARG On-target (%)** | **MGE On-target**  **(%)** |
| --- | --- | --- | --- | --- | --- | --- | --- |
| Bovine fecal (+Abx) a | TELSeq | 5.50 | 170,966 | 123,438 | 27.8 | 41.6 | 4.8 |
| Bovine fecal (+Abx) b | TELSeq | 4.84 | 160,393 | 124,792 | 22.2 | 40.0 | 5.0 |
| Bovine fecal (+Abx) c | TELSeq | 5.05 | 175,691 | 133,256 | 24.2 | 35.8 | 4.7 |
| Bovine fecal (-Abx) a | TELSeq | 1.10 | 70,309 | 68,201 | 3.0 | 17.5 | 0.5 |
| Bovine fecal (-Abx) b | TELSeq | 1.24 | 80,562 | 74,881 | 7.1 | 17.4 | 0.5 |
| Bovine fecal (-Abx) c | TELSeq | 2.23 | 142,793 | 122,837 | 14.0 | 33.0 | 1.6 |
| Bovine fecal (-Abx) a | PacBio | 1.13 | 38,766 | 38,762 | 0.0 | 1.3 | 0.1 |
| Bovine fecal (-Abx) b | PacBio | 1.04 | 35,931 | 35,926 | 0.0 | 1.1 | 0.1 |
| Bovine fecal (-Abx) c | PacBio | 1.48 | 51,115 | 51,105 | 0.0 | 1.2 | 0.1 |
| Bovine fecal (-Abx) a | Illumina | 10.95 | 112,924,341 | __ | __ | 0.4 | 0.2 |
| Bovine fecal (-Abx) b | Illumina | 10.02 | 100,568,957 | __ | __ | 0.4 | 0.2 |
| Bovine fecal (-Abx) c | Illumina | 8.87 | 112,682,117 | __ | __ | 0.4 | 0.1 |
| FMT a | TELSeq | 2.33 | 149,001 | 139,094 | 6.6 | 19.3 | 3.1 |
| FMT b | TELSeq | 2.47 | 147,688 | 144,497 | 2.2 | 13.7 | 3.0 |
| FMT c | TELSeq | 0.91 | 86,647 | 83,820 | 3.3 | 18.6 | 2.9 |
| FMT a | PacBio | 1.40 | 5,137 | 5,137 | 0.0 | 1.5 | 0.6 |
| FMT b | PacBio | 2.10 | 75,841 | 75,652 | 0.2 | 1.5 | 1.2 |
| FMT c | PacBio | 1.00 | 32,301 | 32,282 | 0.1 | 1.6 | 1.4 |
| FMT a | Illumina | 10.54 | 118,610,715 | __ | __ | 0.94 | 1.2 |
| FMT b | Illumina | 11.55 | 140,905,584 | __ | __ | 0.68 | 1.0 |
| FMT c | Illumina | 10.05 | 122,693,830 | __ | __ | 0.74 | 1.0 |
| SOIL a | TELSeq | 5.20 | 332,406 | 311,302 | 6.3 | 48.5 | 2.7 |
| SOIL b | TELSeq | 4.33 | 283,317 | 261,935 | 7.5 | 40.6 | 2.4 |
| SOIL c | TELSeq | 1.78 | 120,070 | 117,896 | 1.8 | 26.1 | 1.3 |
| SOIL a | PacBio | 0.68 | 30,846 | 30,845 | 0.0 | 0.2 | 0.7 |
| SOIL b | PacBio | 2.21 | 98,621 | 98,621 | 0.0 | 0.2 | 1.0 |
| SOIL c | PacBio | 0.84 | 38,462 | 38,462 | 0.0 | 0.2 | 0.8 |
| SOIL a | Illumina | 11.69 | 147,538,159 | __ | __ | 0.15 | 2.9 |
| SOIL b | Illumina | 12.19 | 147,770,845 | __ | __ | 0.16 | 3.0 |
| SOIL c | Illumina | 11.74 | 153,805,143 | __ | __ | 0.16 | 2.8 |
| MOCK a | TELSeq | 4.79 | 164,742 | 96,241 | 41.6% | 90.4 | 7.3 |
| MOCK b | TELSeq | 4.20 | 138,000 | 89,049 | 35.5% | 62.3 | 5.4 |
| MOCK c | TELSeq | 5.79 | 194,900 | 122,031 | 37.4% | 59.1 | 5.4 |
| MOCK | GridION | 13.46 | 3,504,550 | __ | __ | 2.6 | 2.9 |
| MOCK | PromethION | 122.46 | 33,108,987 | __ | __ | 2.7 | 2.9 |
