# Supplementary material for: Target-enriched long-read sequencing (TELSeq) contextualizes antimicrobial resistance genes in metagenomes
Source: Microbiome. 2022 Nov 2;10:185. doi: 10.1186/s40168-022-01368-y (PMC9628182; doi:10.1186/s40168-022-01368-y)
Supplement: Supplementary file 7 — Additional file 6: Supplementary Table 2. Summary of ARG and MGE relative composition and richness identified by TELSeq, PacBio, and Illumina reads generated from +Abx, −Abx, FMT, SOIL and MOCK replicates. [file 40168_2022_1368_MOESM6_ESM.docx]

**Supplementary Table 2.** Summary of ARG and MGE relative composition and richness identified by TELSeq, PacBio, and Illumina reads generated from +Abx, -Abx, FMT, SOIL and MOCK replicates. ARG composition is characterized by Type (■Drug; ■Metal; ■Multi-compound; and ■Biocide), and MGE composition is summarized by horizontal transfer mechanism (■Plasmid; ■Phage; ■Transposable element (TE); ■Insertional sequence (IS); ■Integrative conjugative element (ICE); and ■Virus).

| **Sample** | **Sequencing platform** | **ARG Composition** | **ARG Class richness** | **ARG Mechanism richness** | **ARG Group richness** | **MGE Composition** | **MGE Accession richness** |
| --- | --- | --- | --- | --- | --- | --- | --- |
| Bovine fecal (+Abx) a | TELSeq | 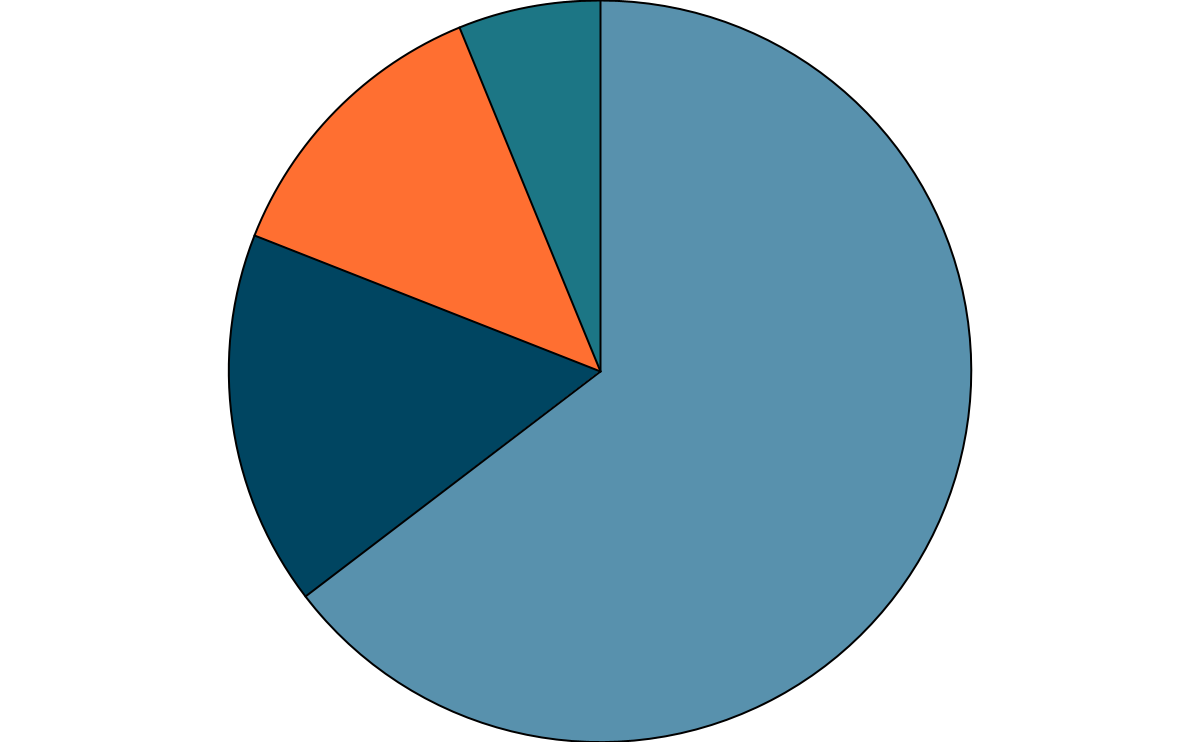 | 33 | 74 | 545 | 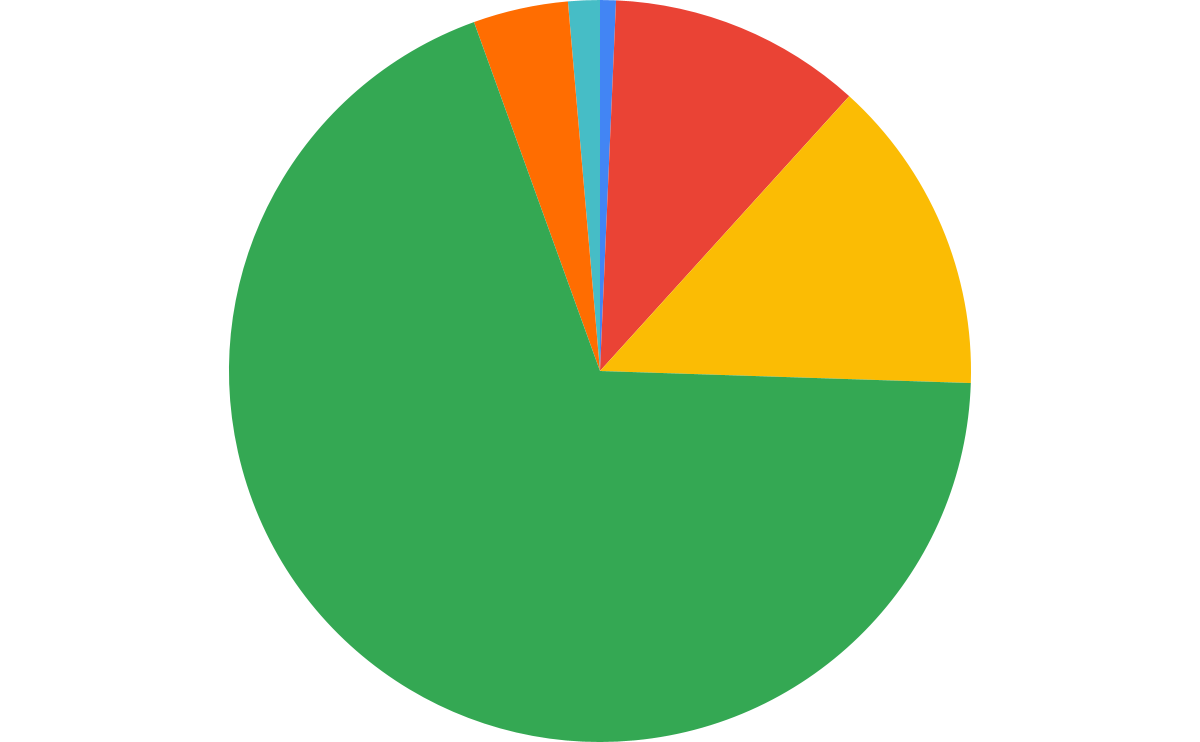 | 152 |
| Bovine fecal (+Abx) b | TELSeq | 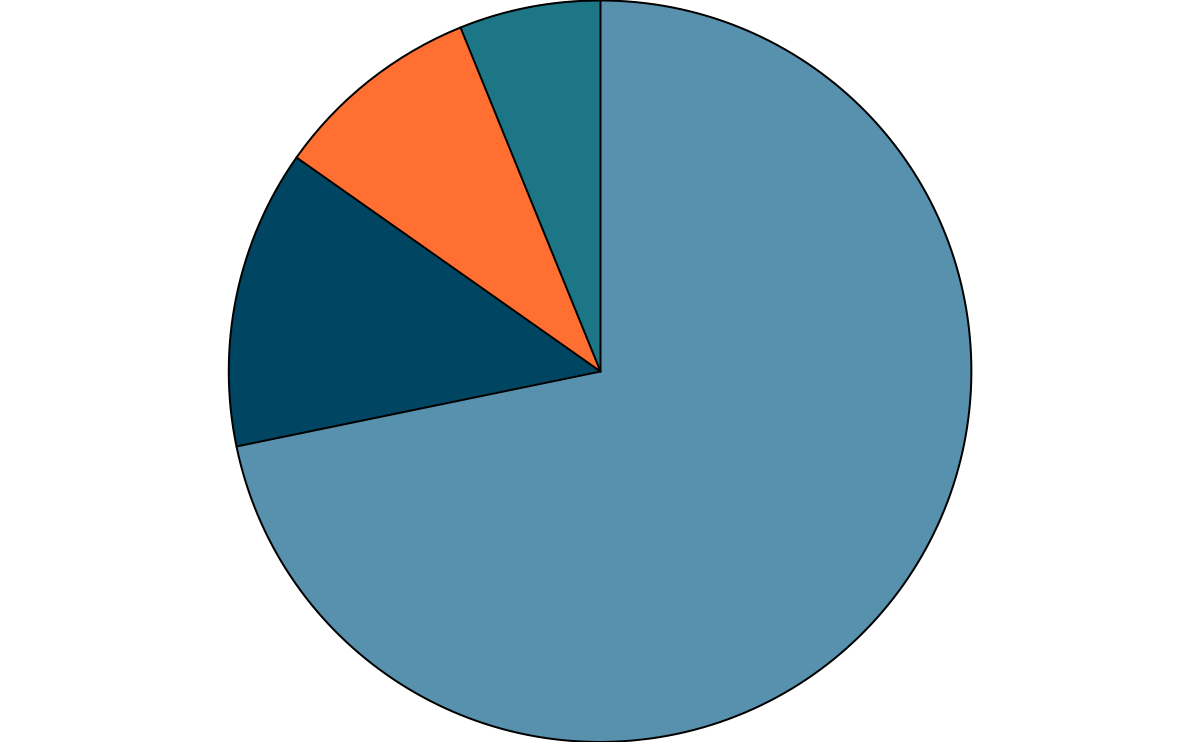 | 35 | 76 | 494 | 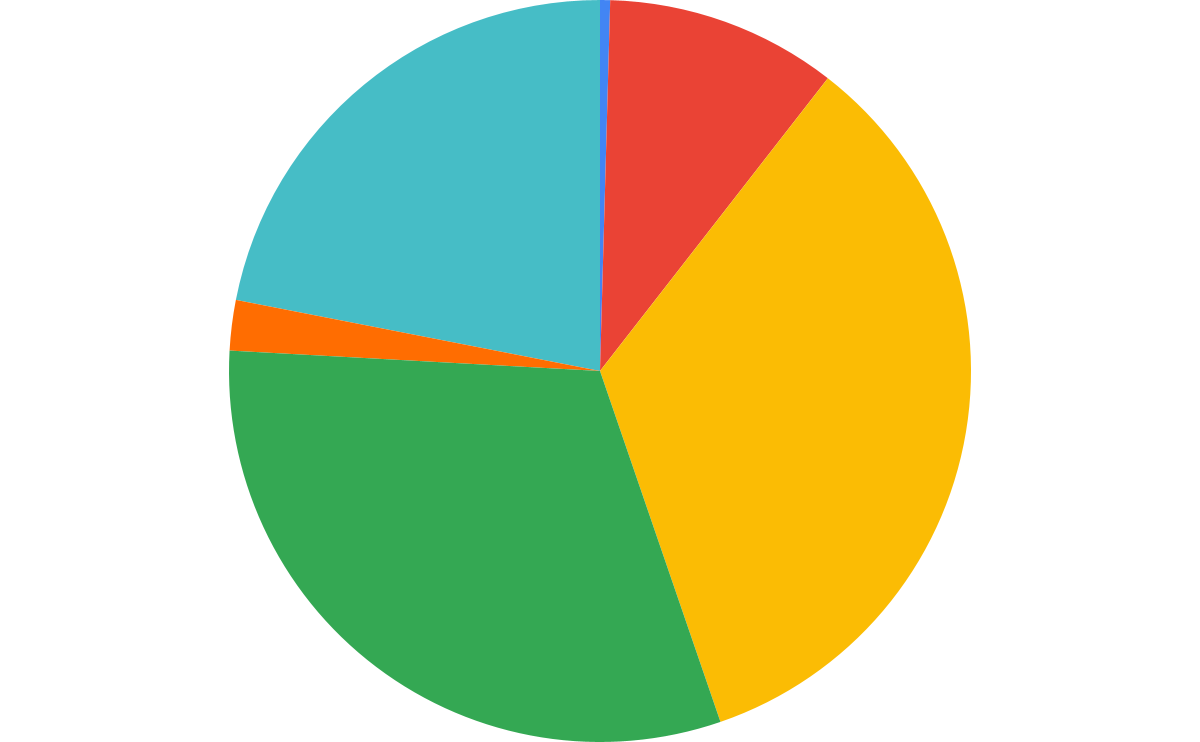 | 234 |
| Bovine fecal (+Abx) c | TELSeq | 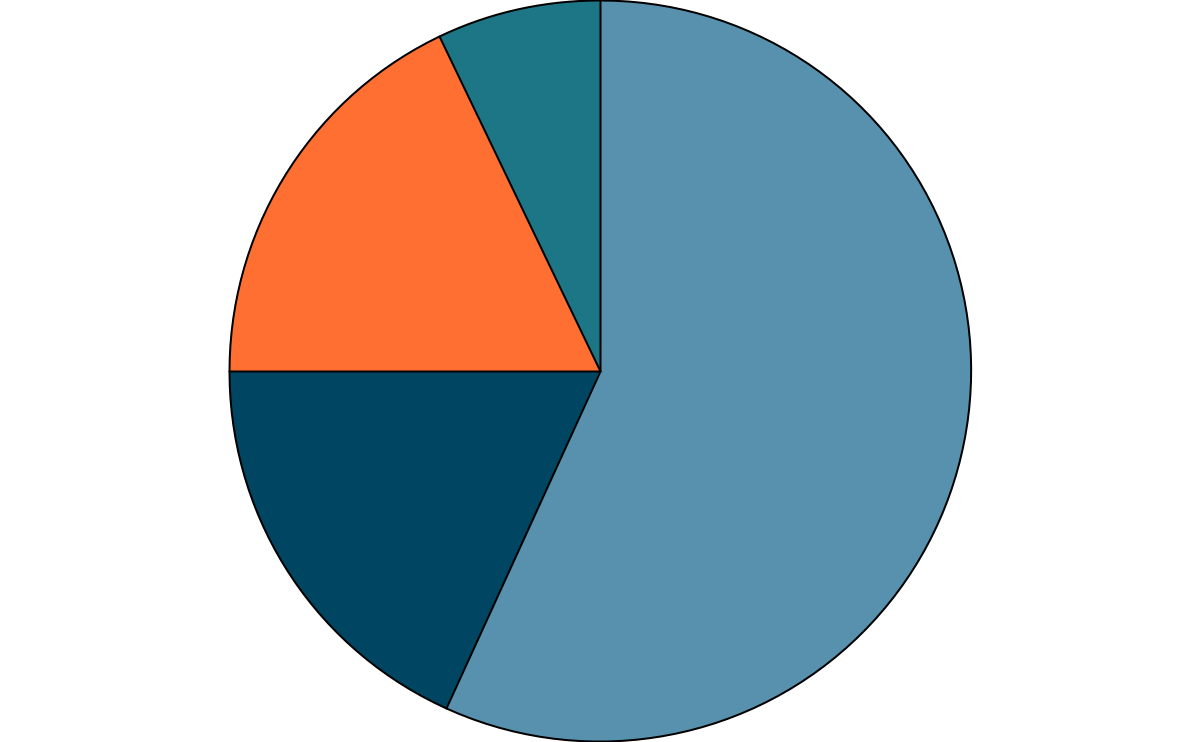 | 37 | 88 | 678 | 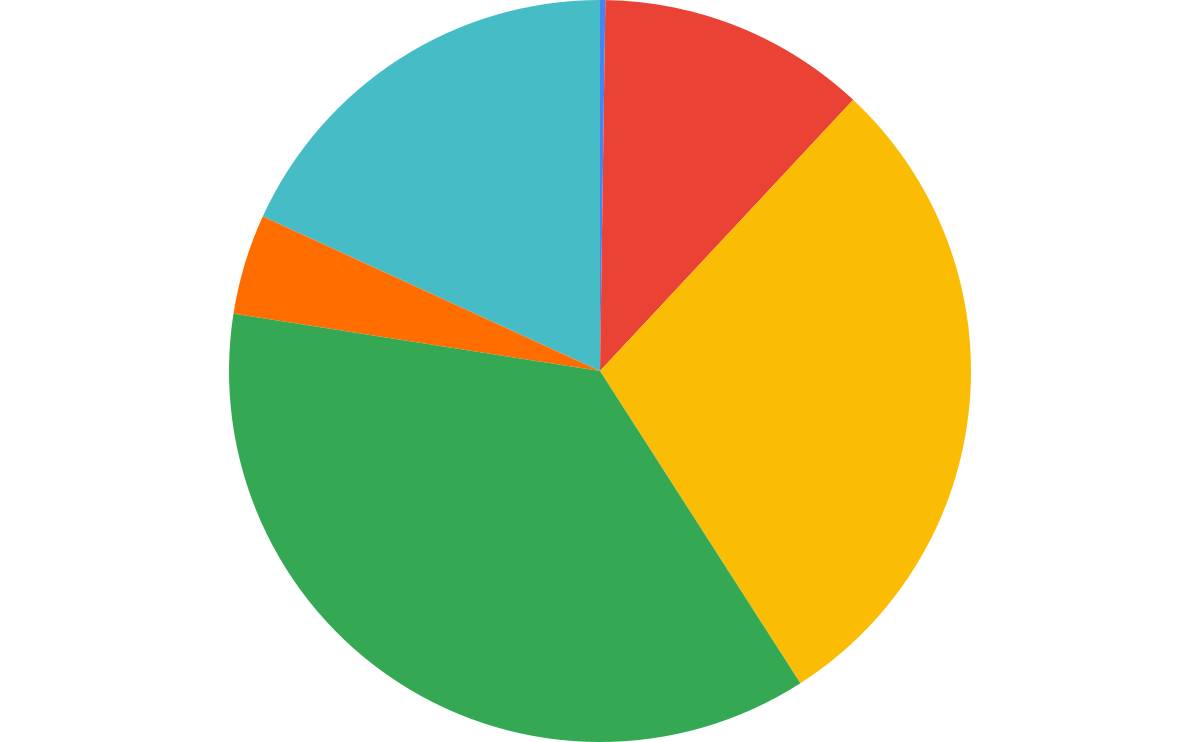 | 438 |
| Bovine fecal (-Abx) a | TELSeq | 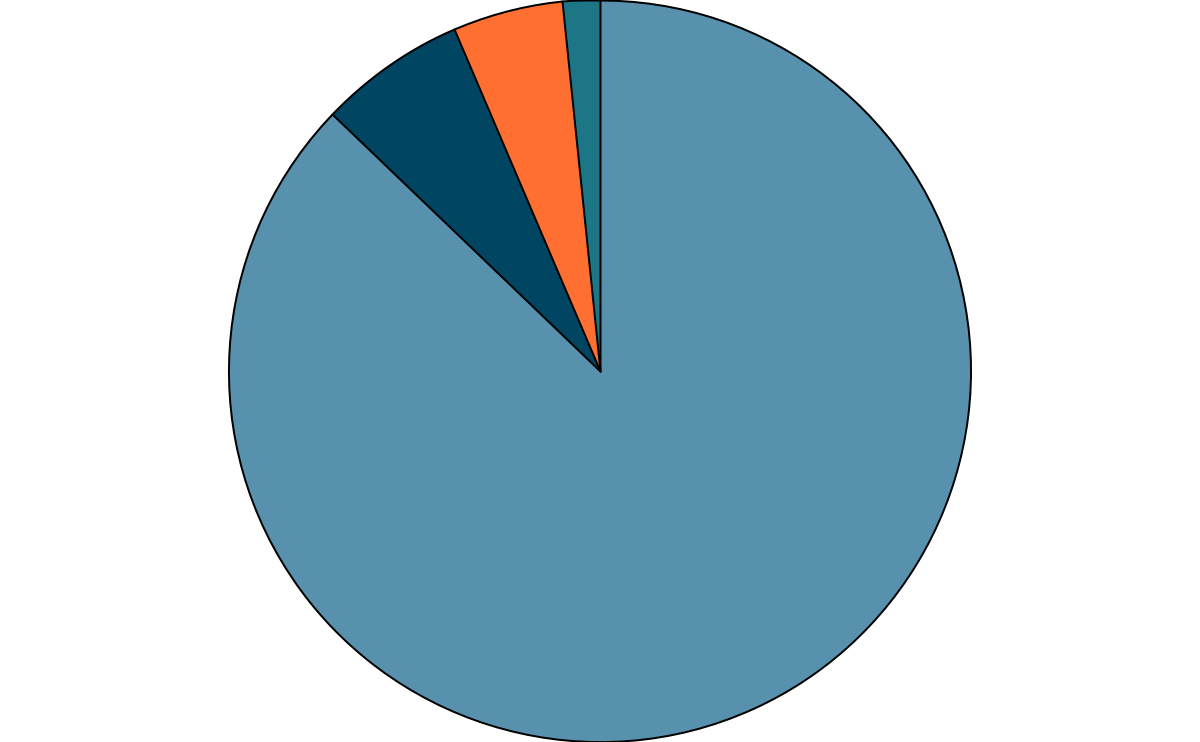 | 37 | 21 | 249 | 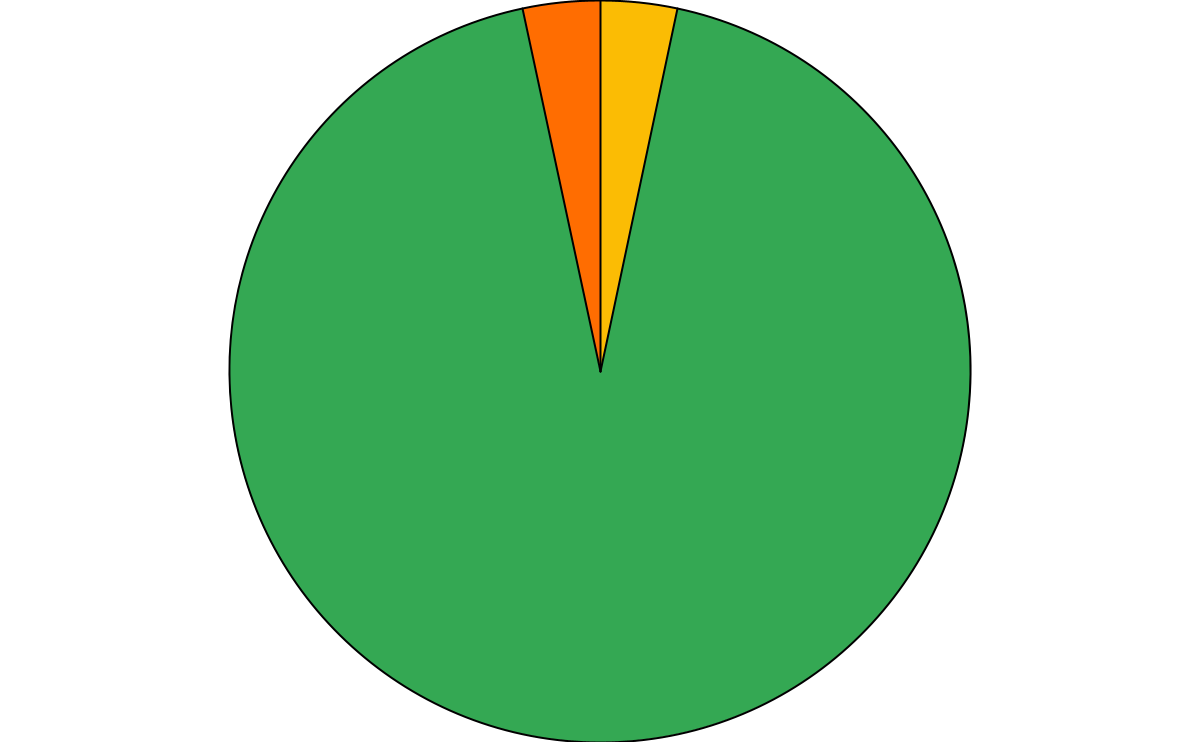 | 30 |
| Bovine fecal (-Abx) b | TELSeq | 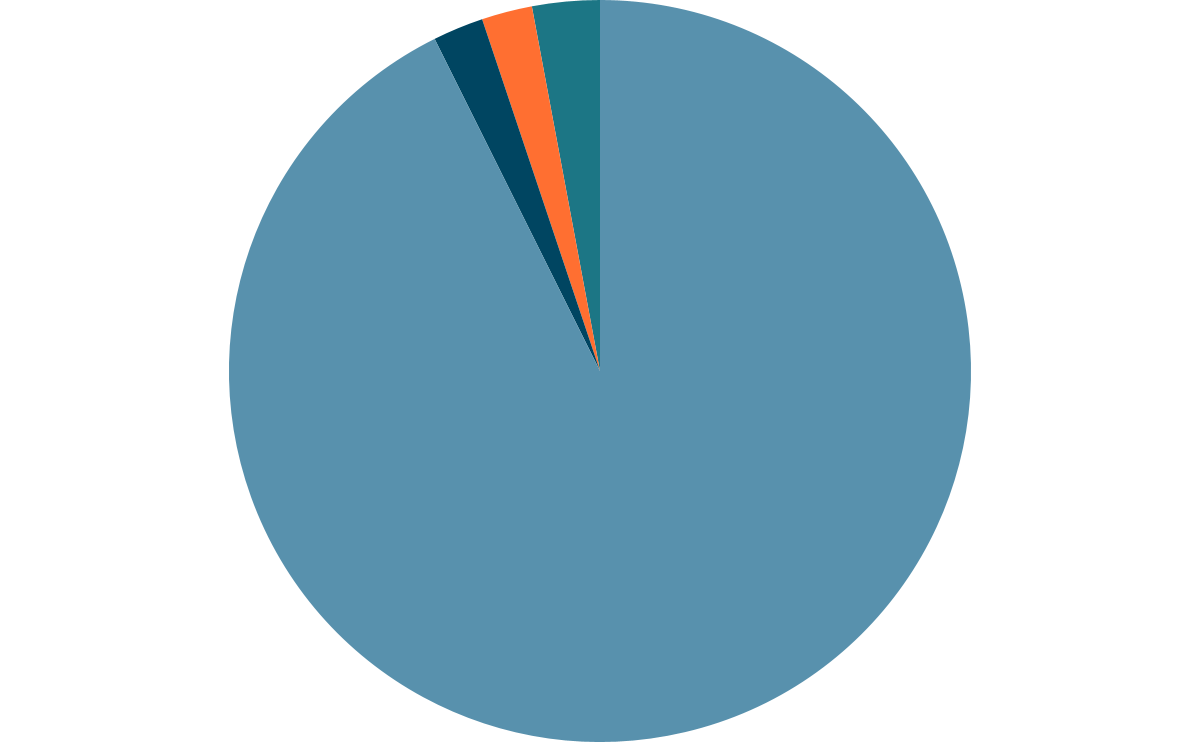 | 27 | 18 | 180 | 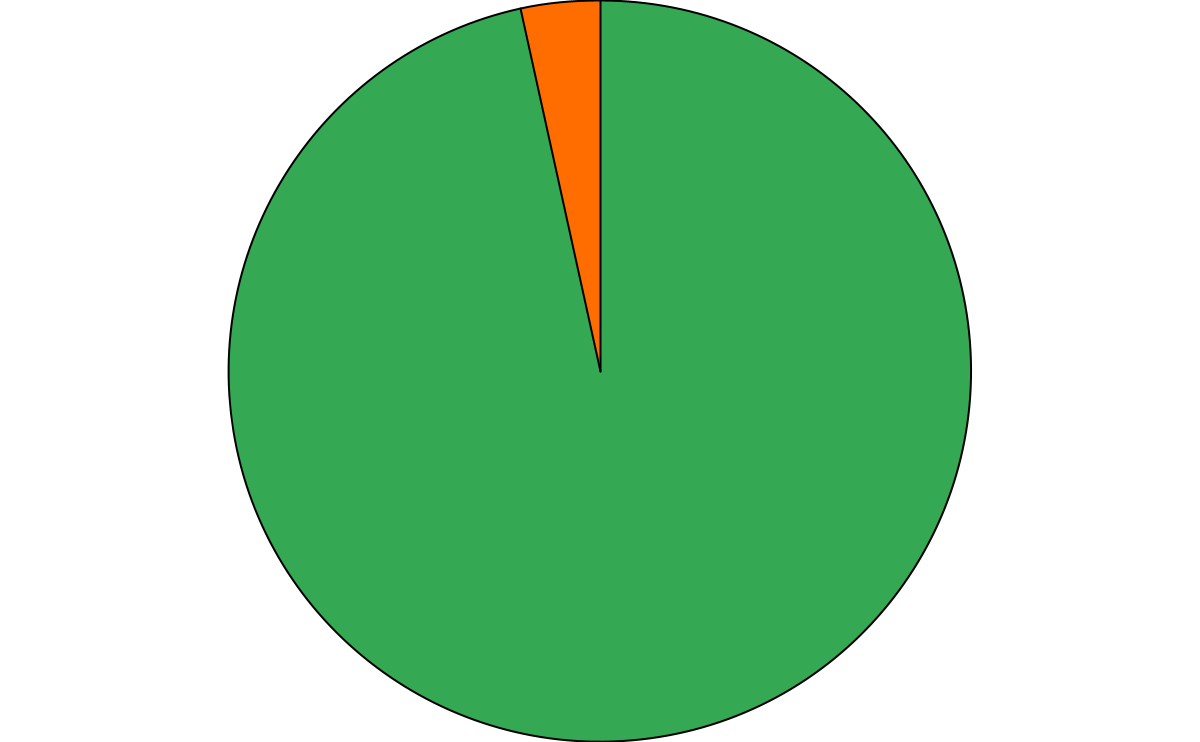 | 29 |
| Bovine fecal (-Abx) c | TELSeq | 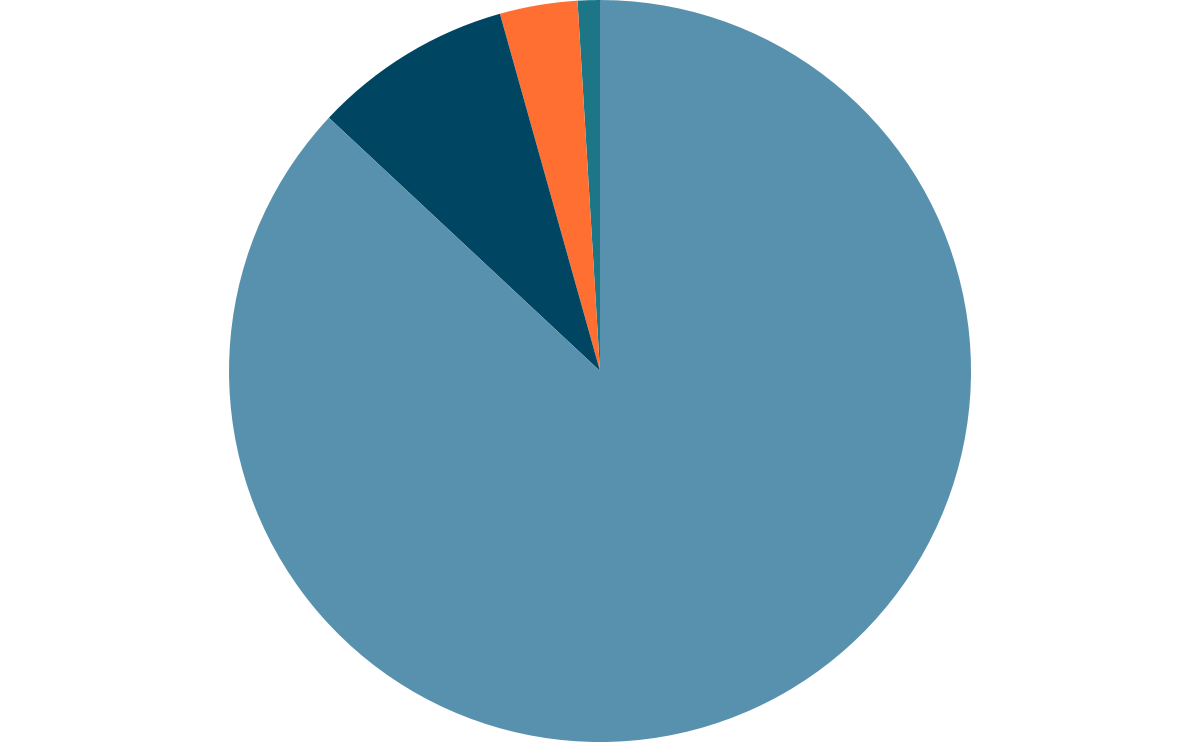 | 47 | 28 | 271 | 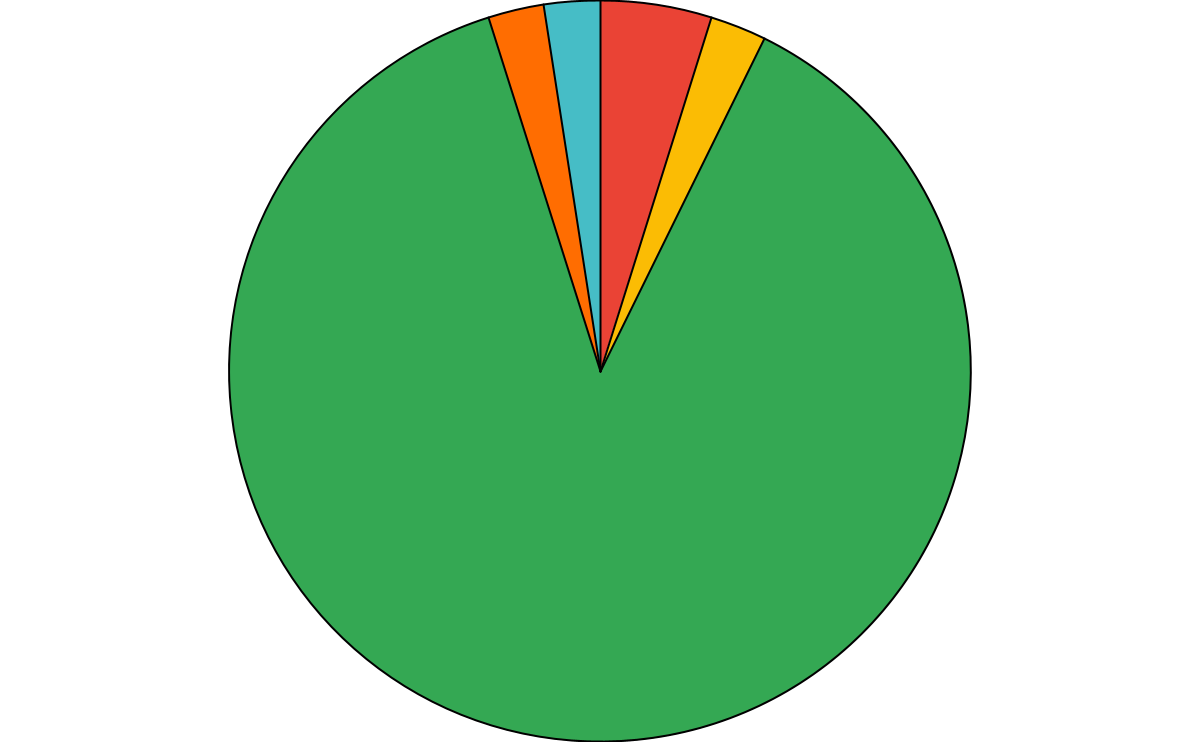 | 44 |
| Bovine fecal (-Abx) a | PacBio | 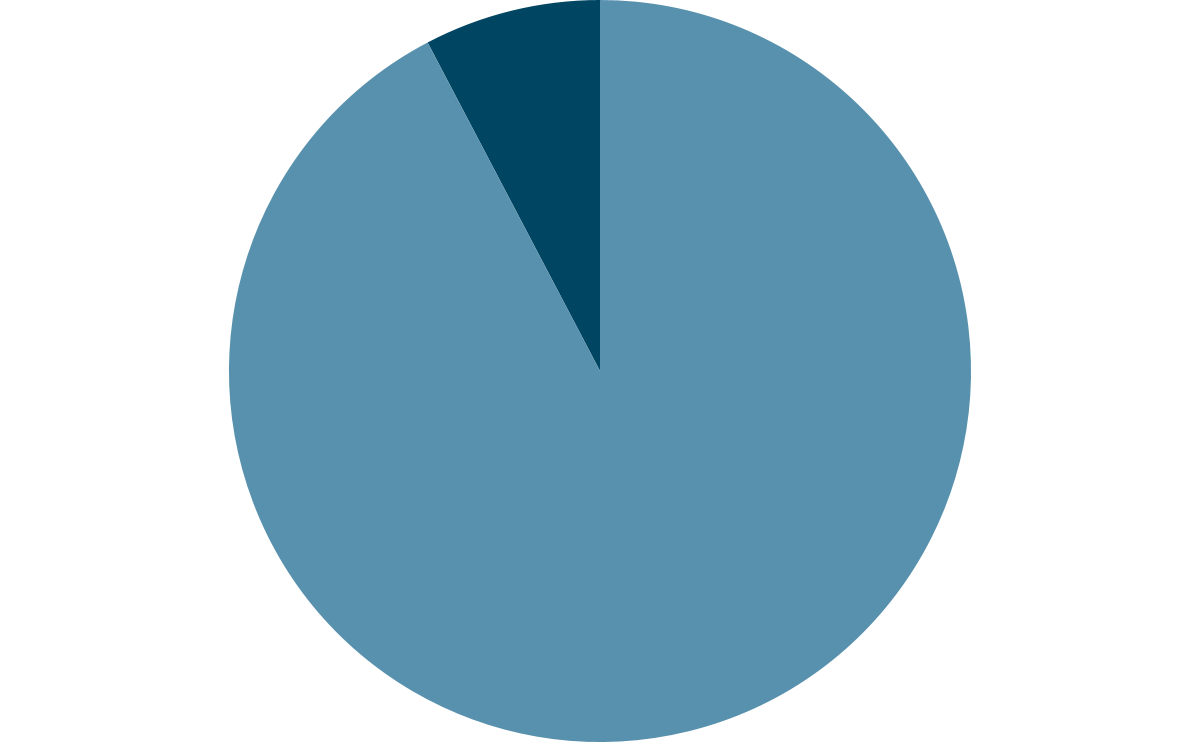 | 19 | 15 | 111 | 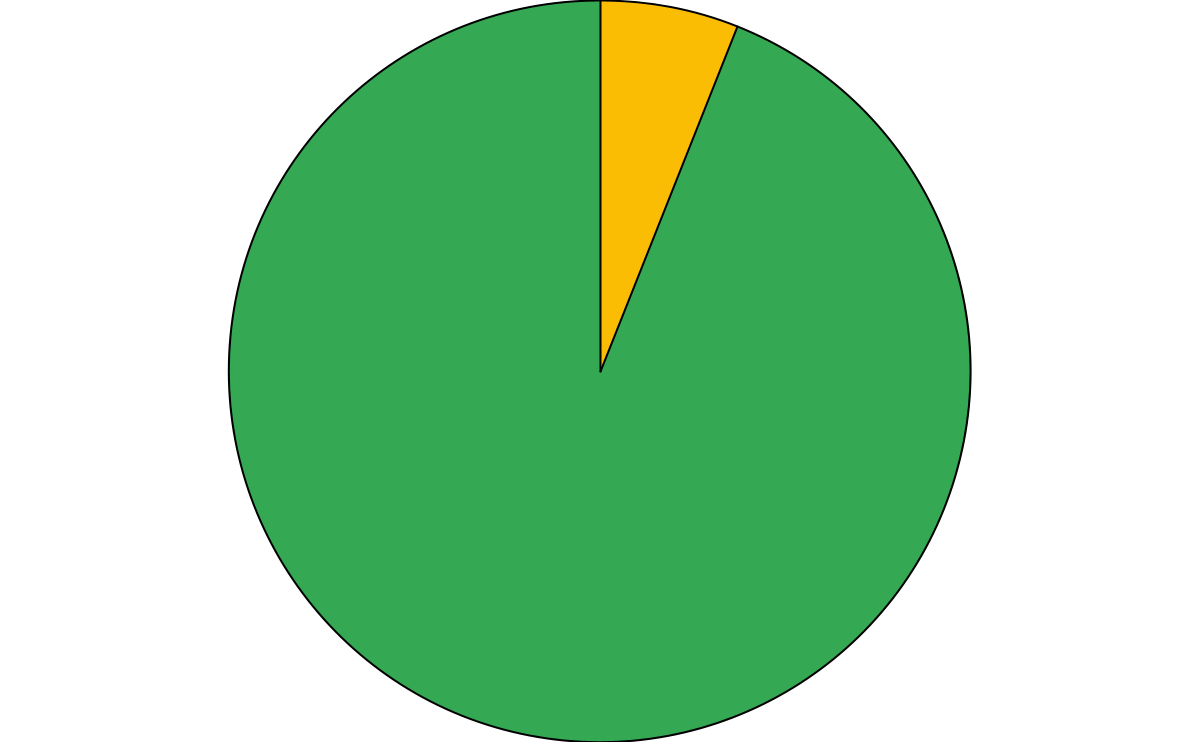 | 34 |
| Bovine fecal (-Abx) b | PacBio | 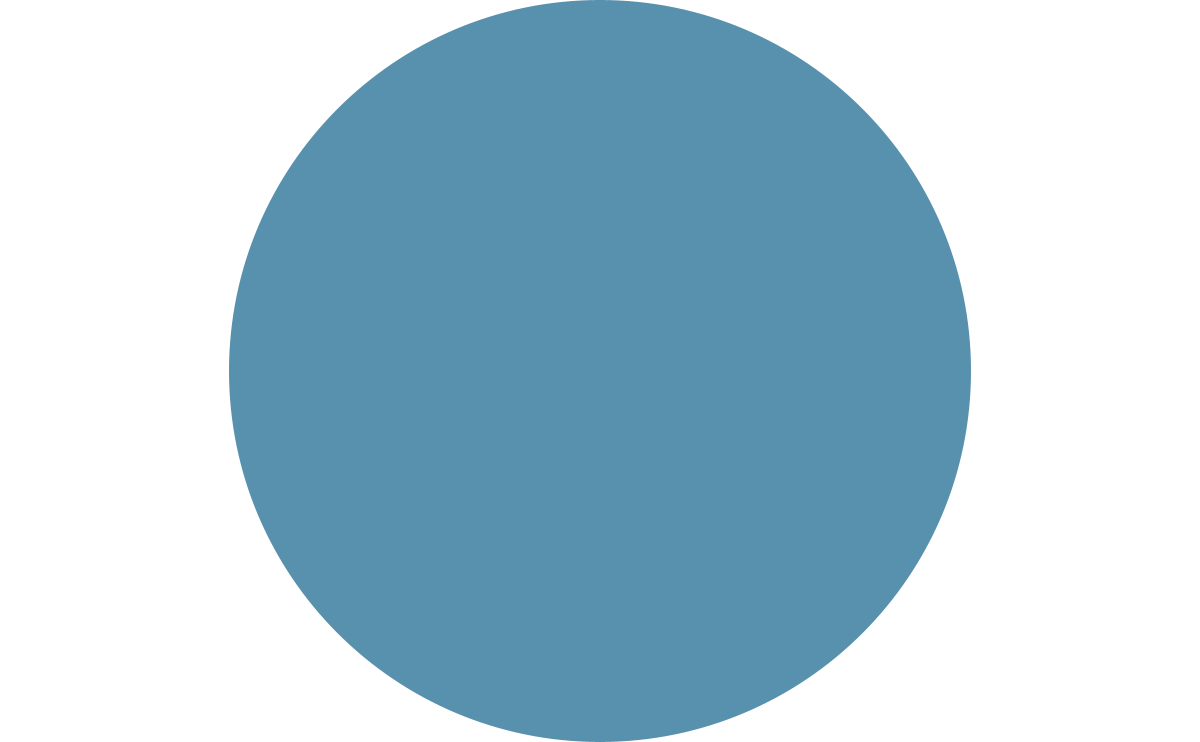 | 17 | 14 | 102 | 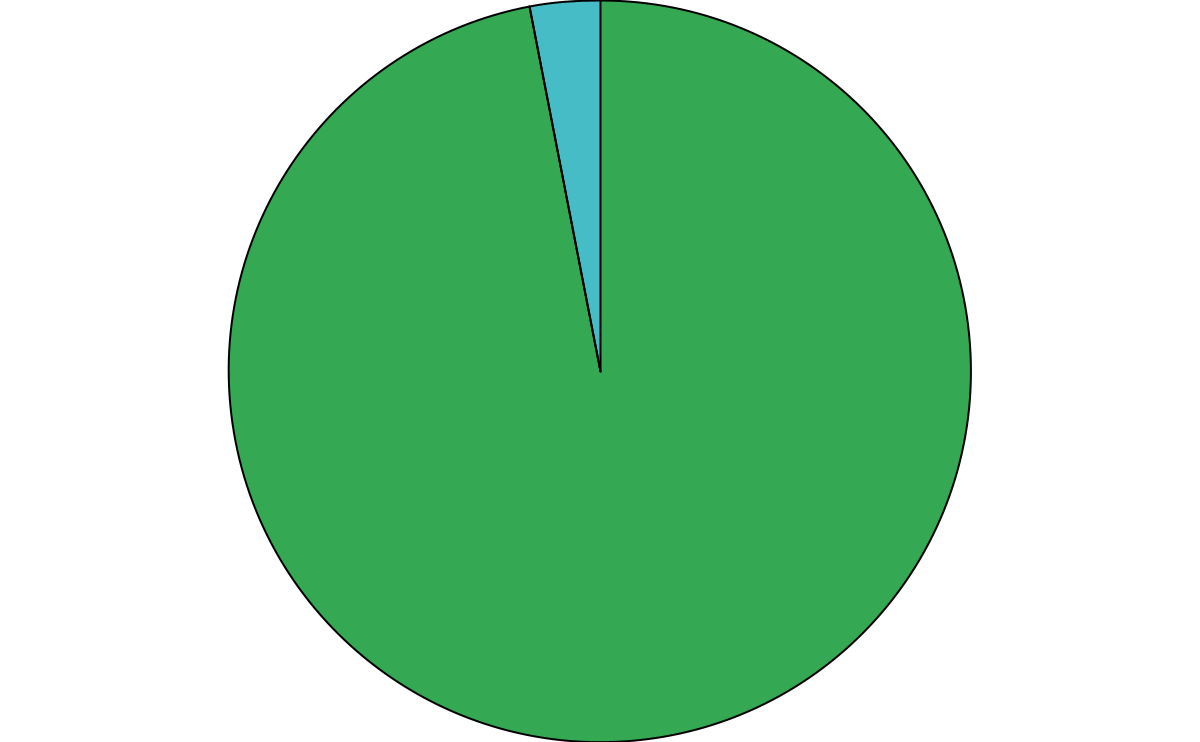 | 34 |
| Bovine fecal (-Abx) c | PacBio | 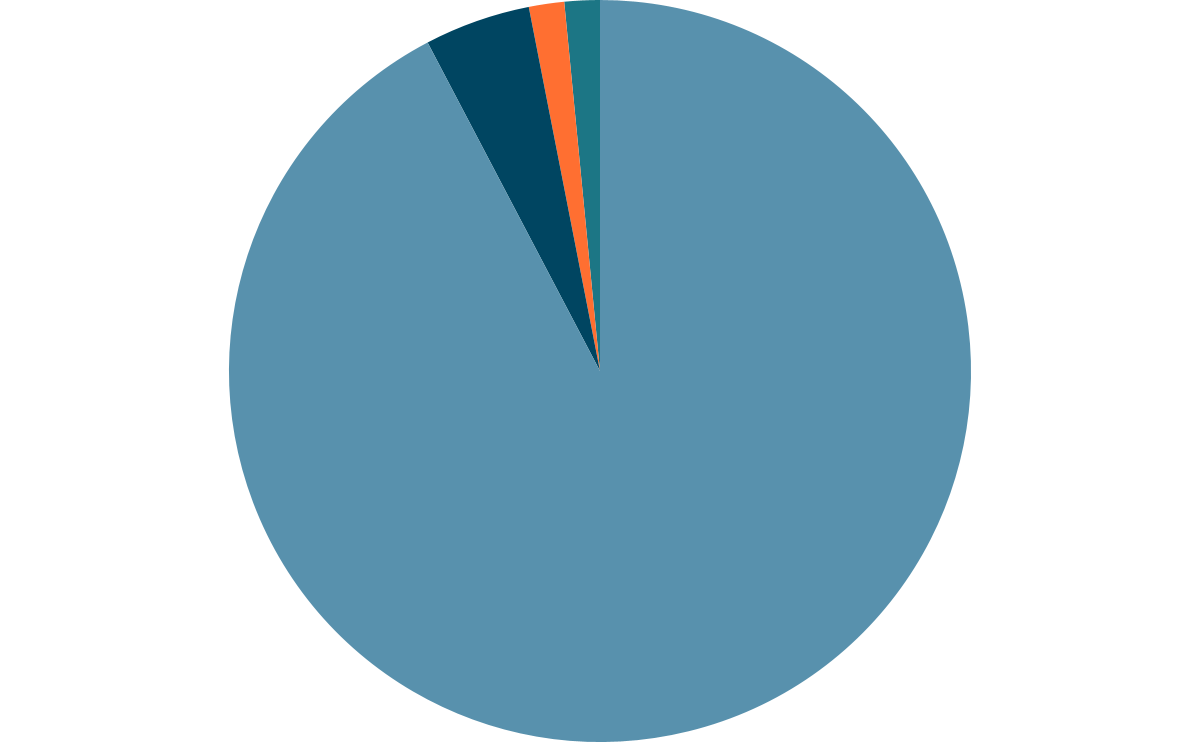 | 19 | 15 | 115 | 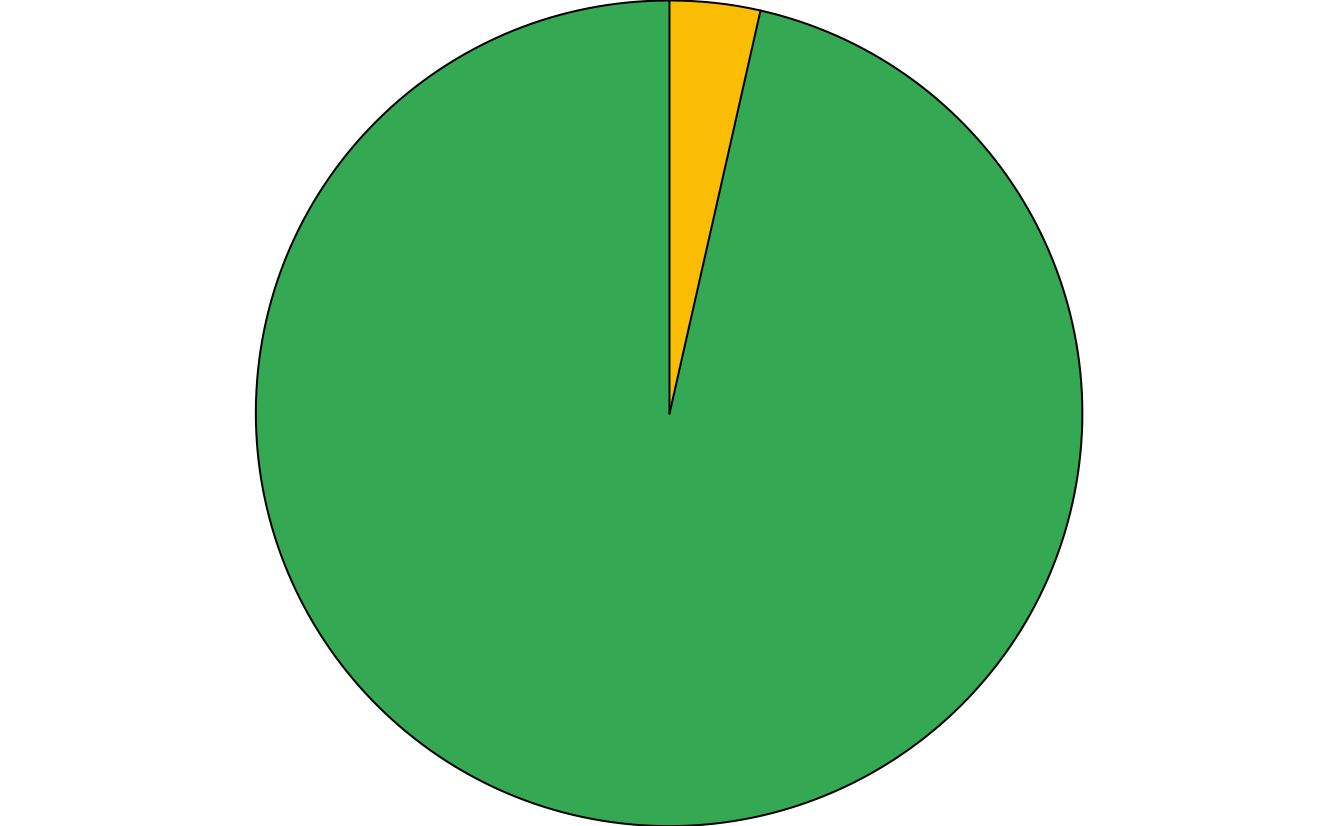 | 30 |
| Bovine fecal (-Abx) a | Illumina | 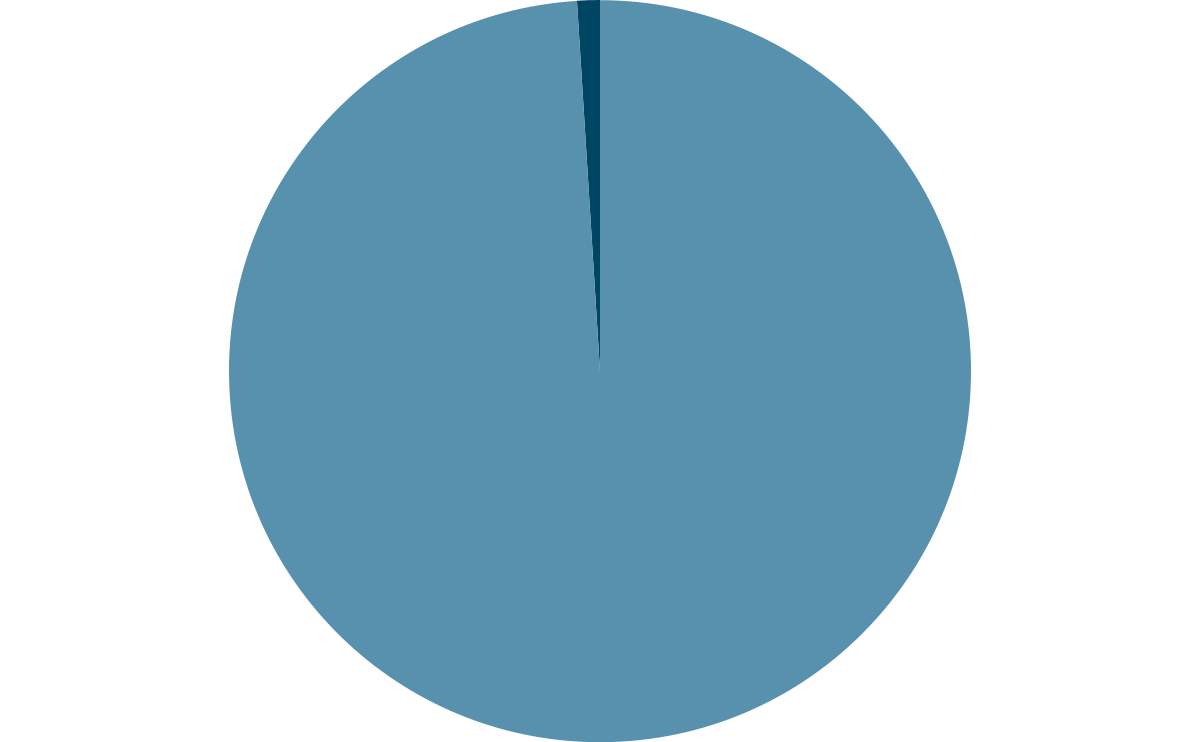 | 10 | 6 | 22 | 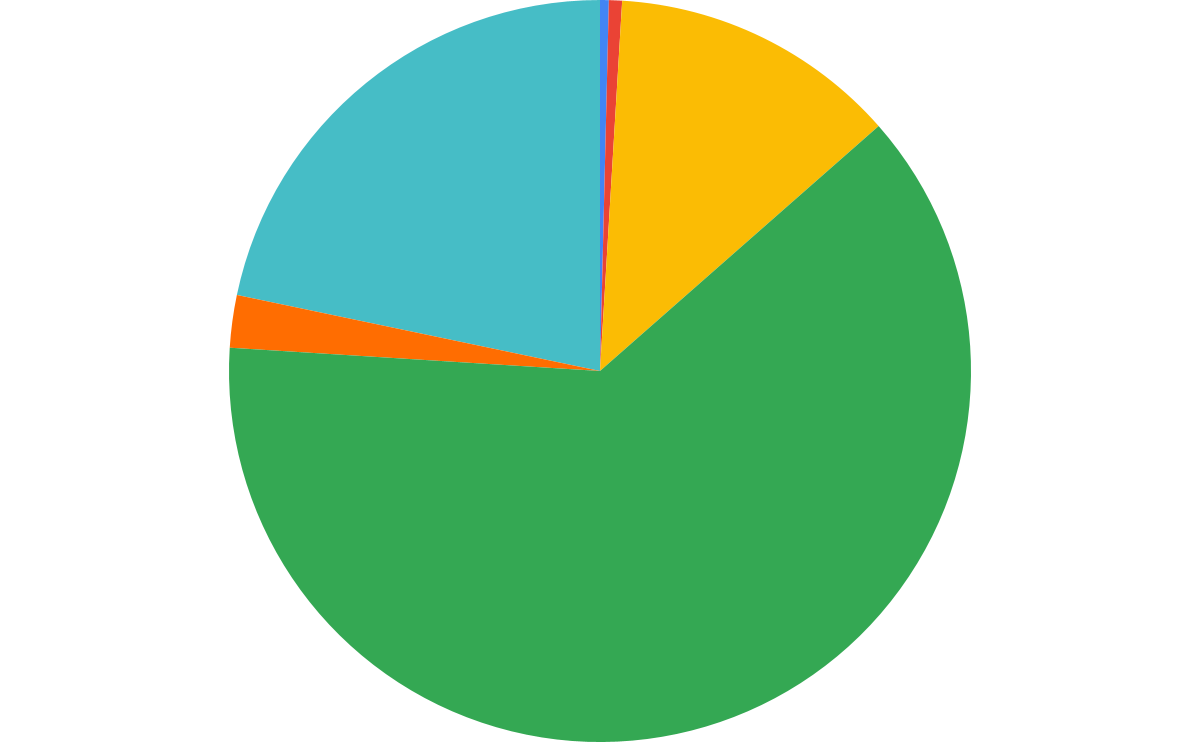 | 528 |
| Bovine fecal (Abx) b | Illumina | 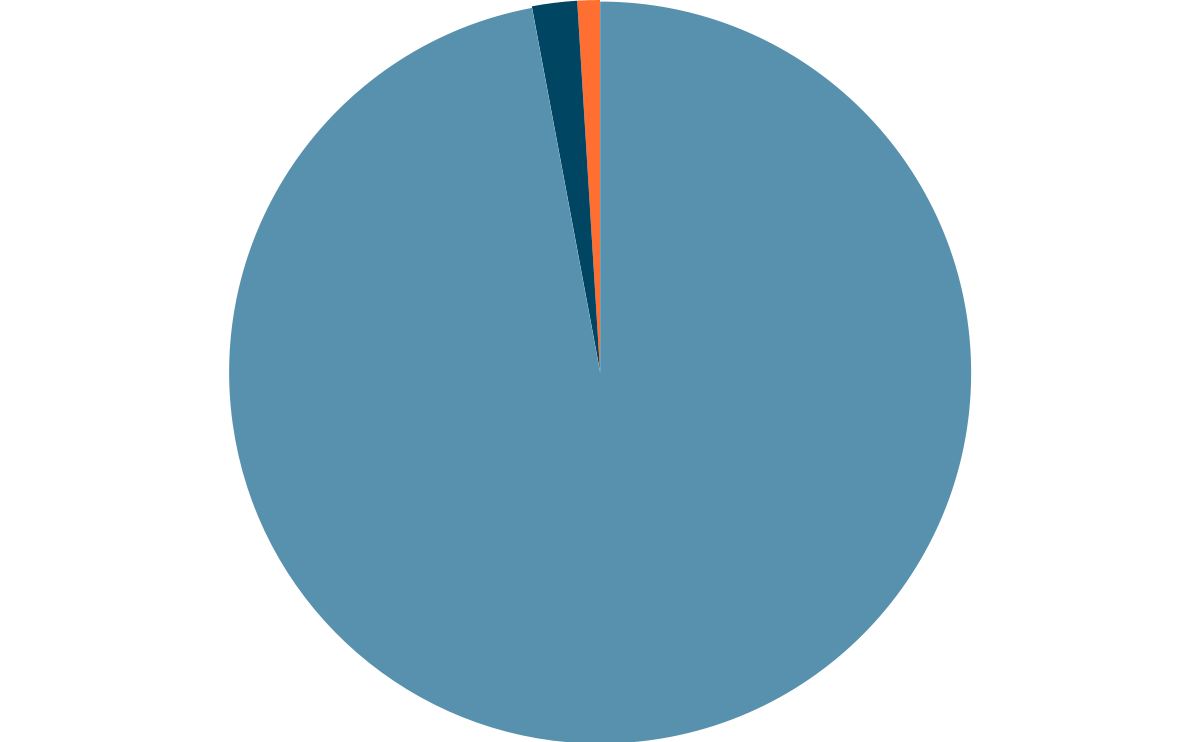 | 11 | 7 | 24 | 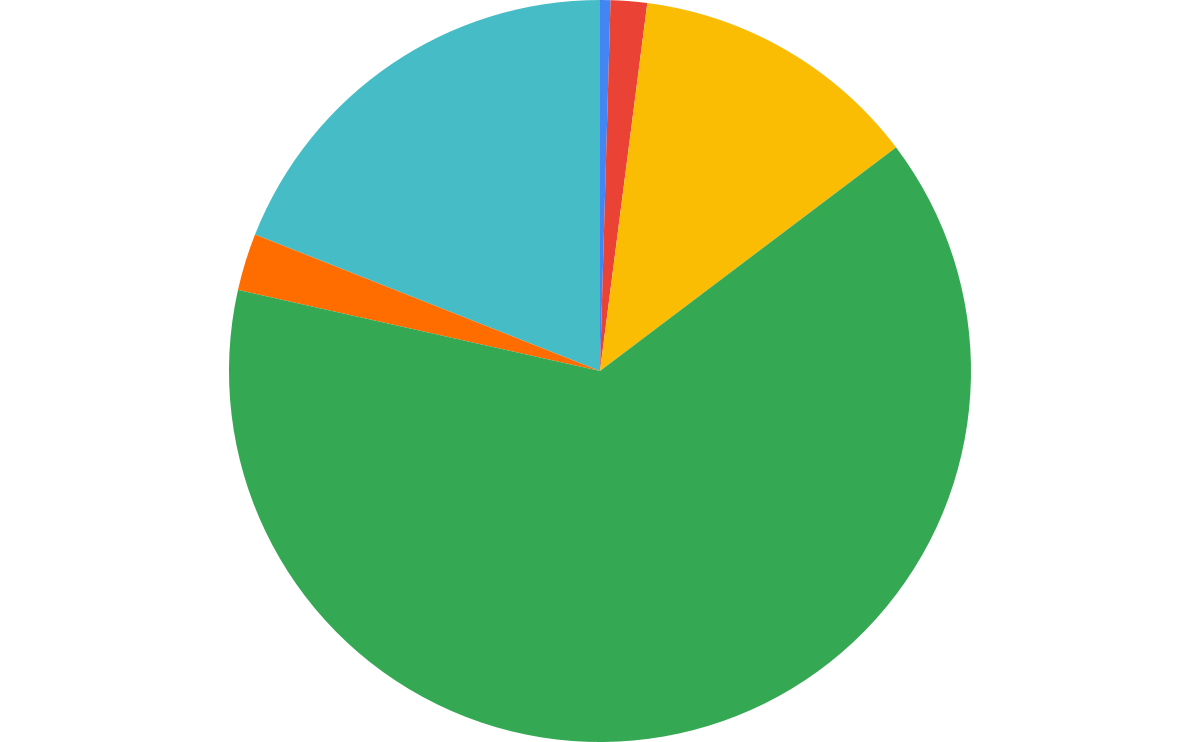 | 442 |
| Bovine fecal (-Abx) c | Illumina | 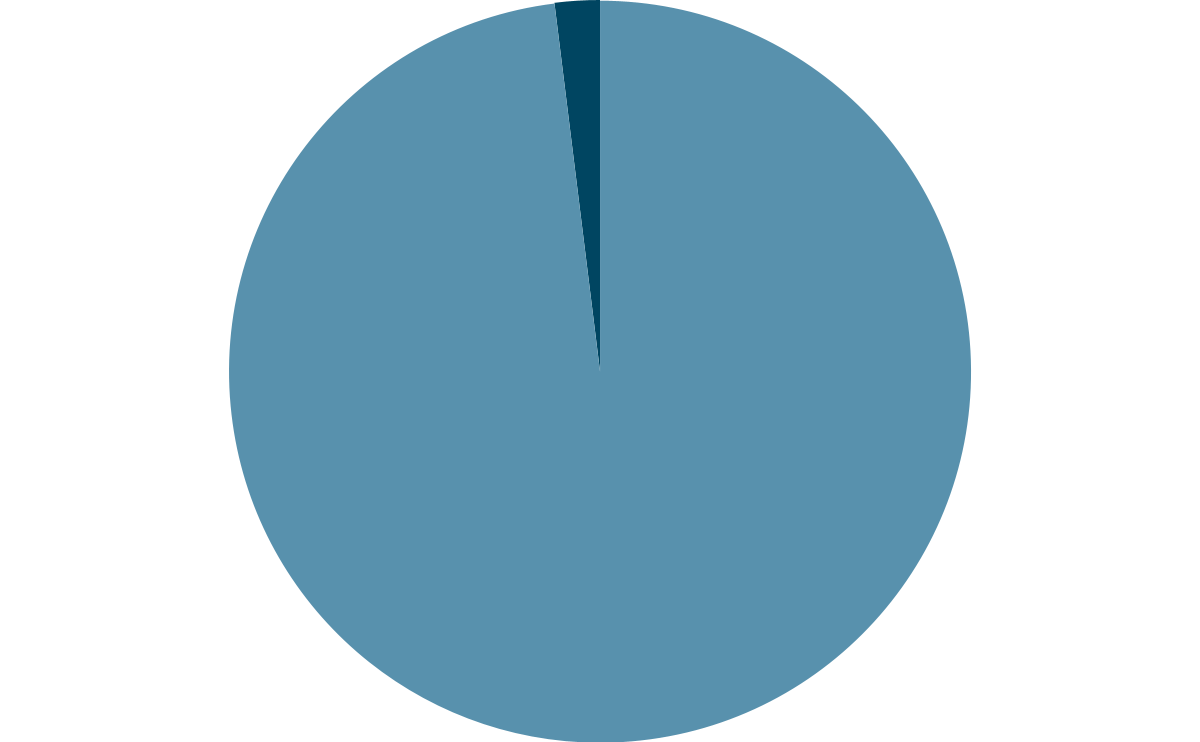 | 11 | 6 | 23 | 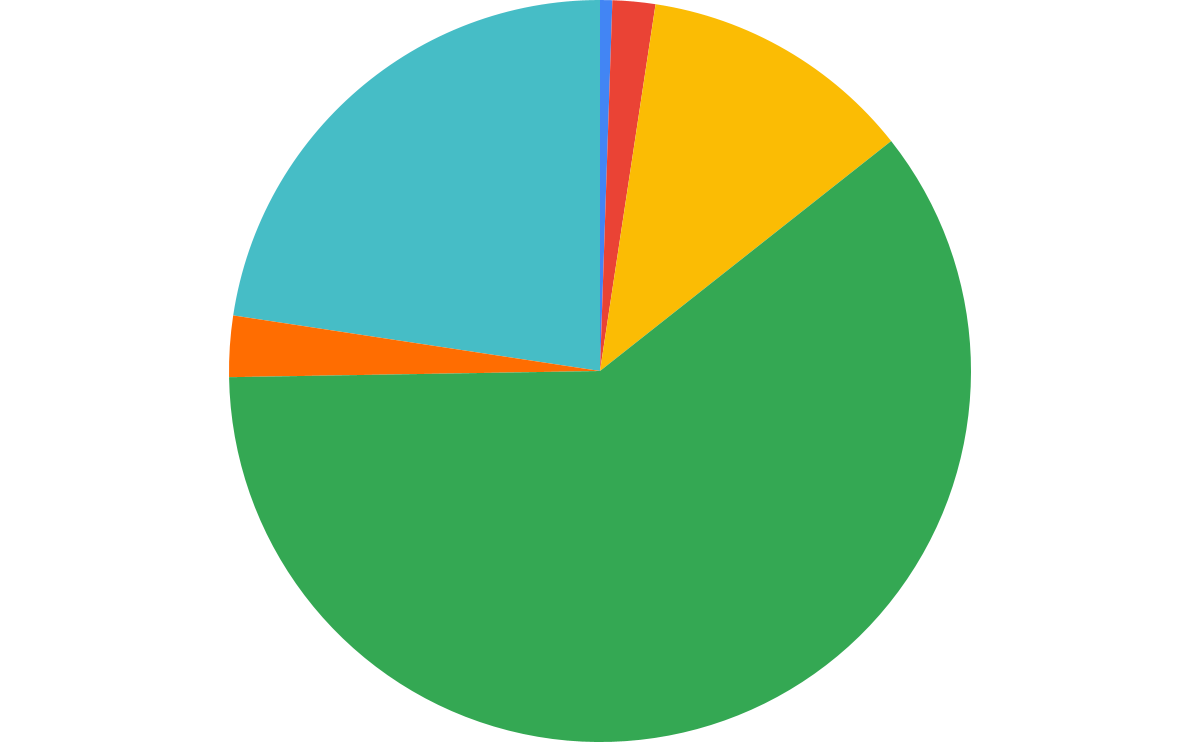 | 376 |
| FMT a | TELSeq | 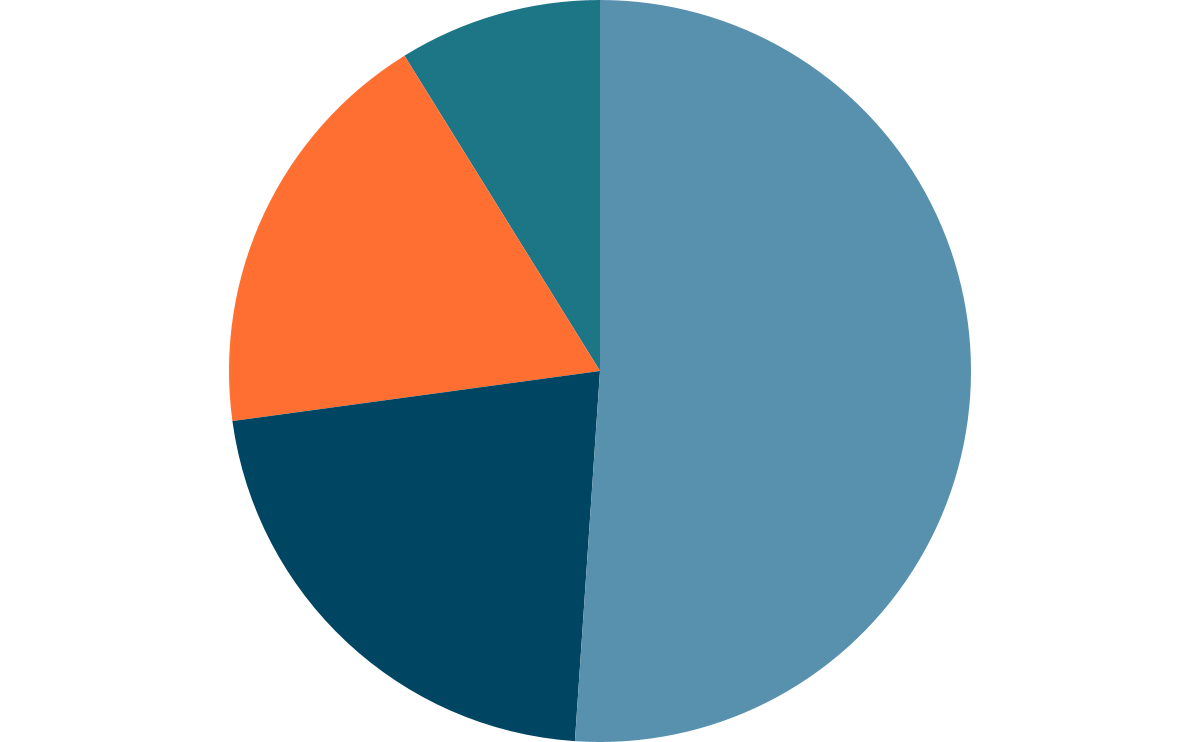 | 84 | 38 | 564 | 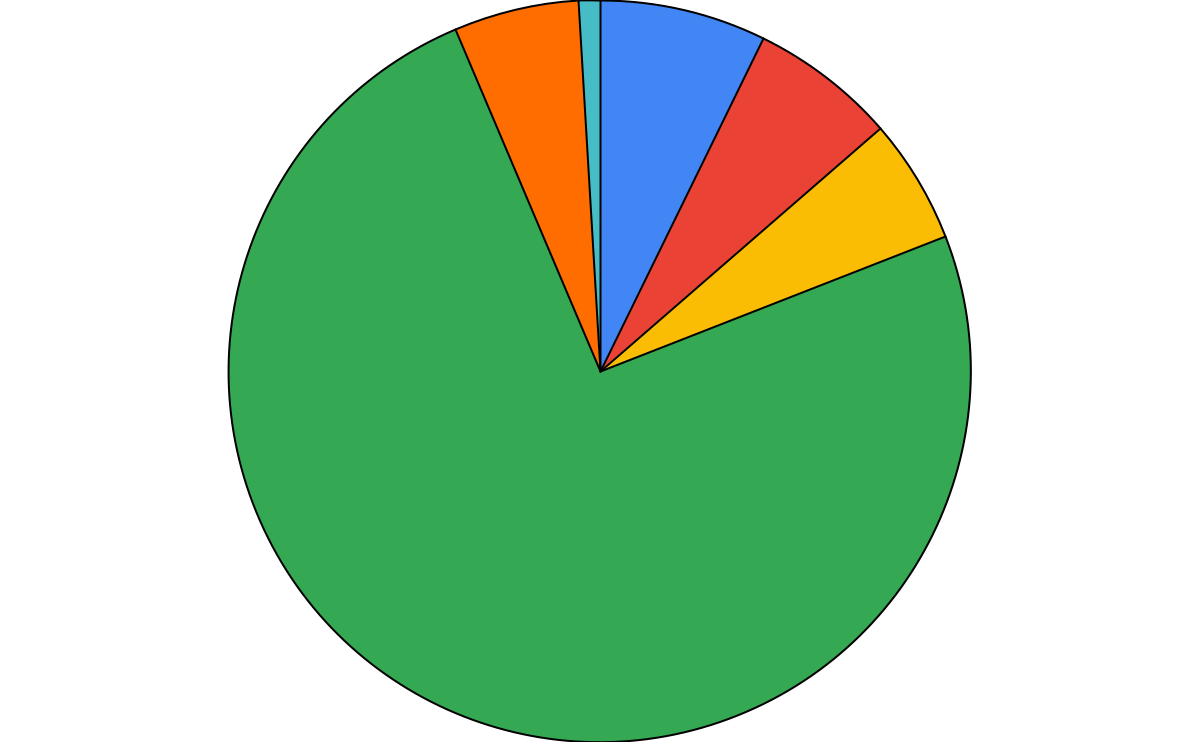 | 120 |
| FMT b | TELSeq | 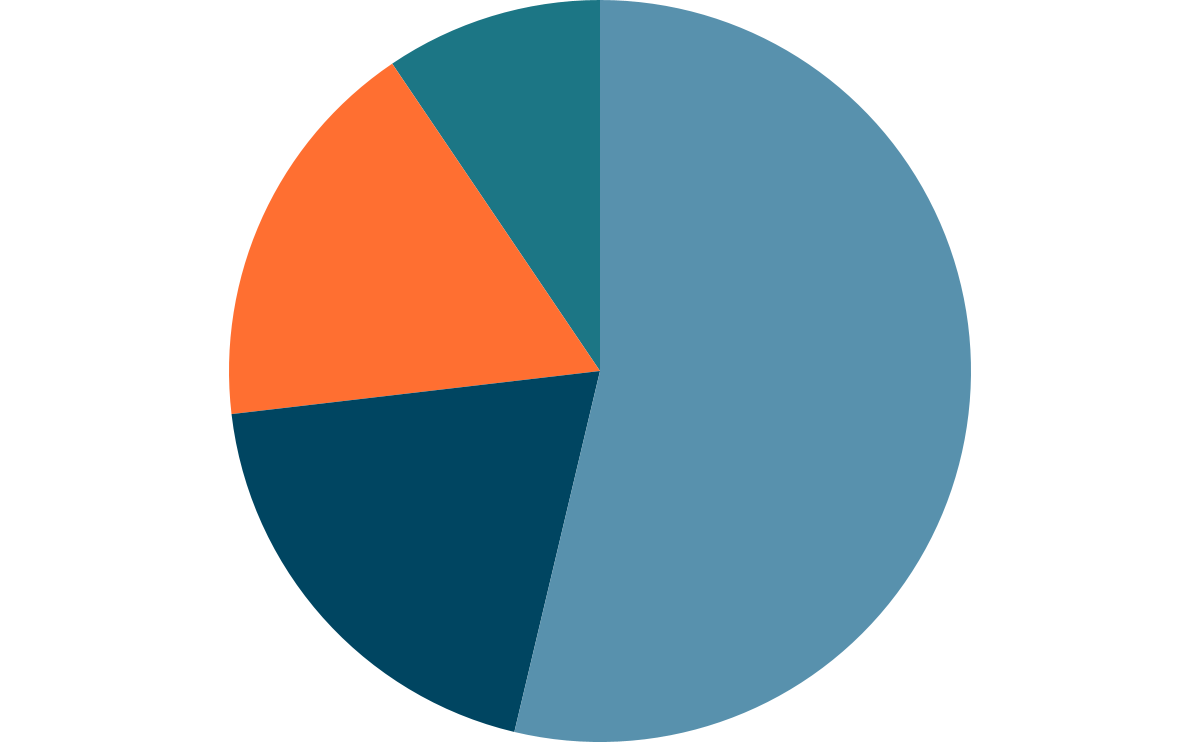 | 79 | 36 | 479 | 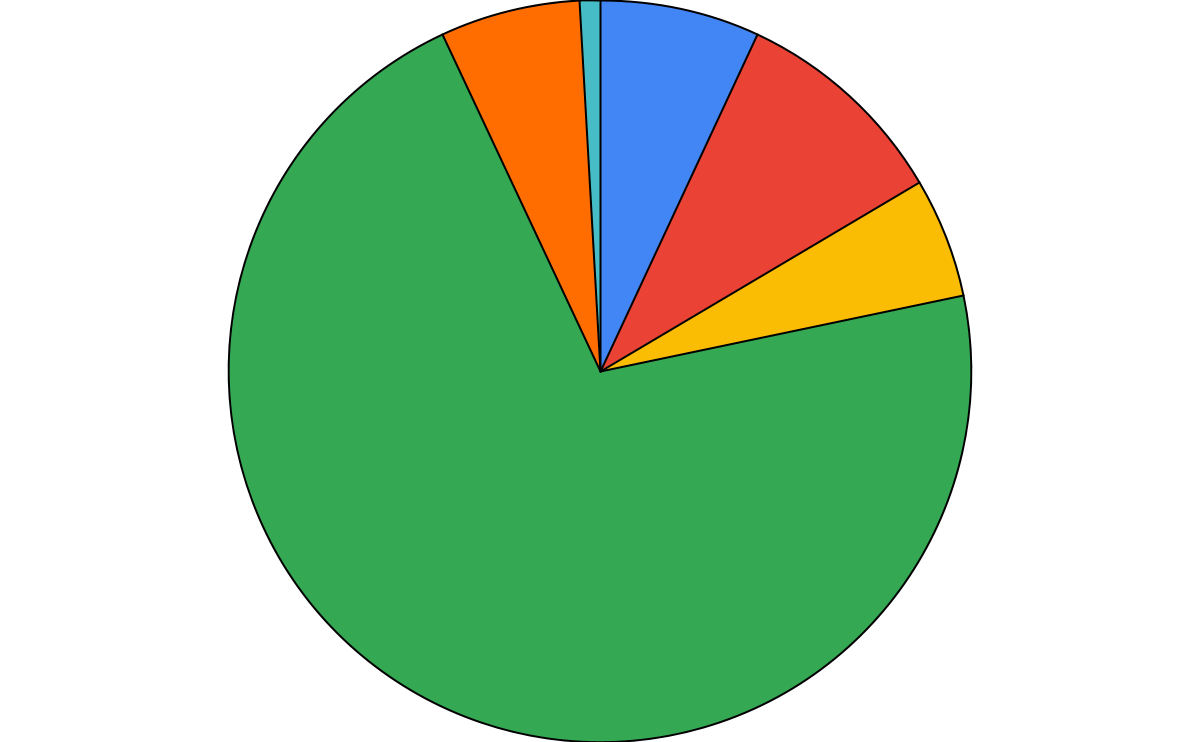 | 117 |
| FMT c | TELSeq | 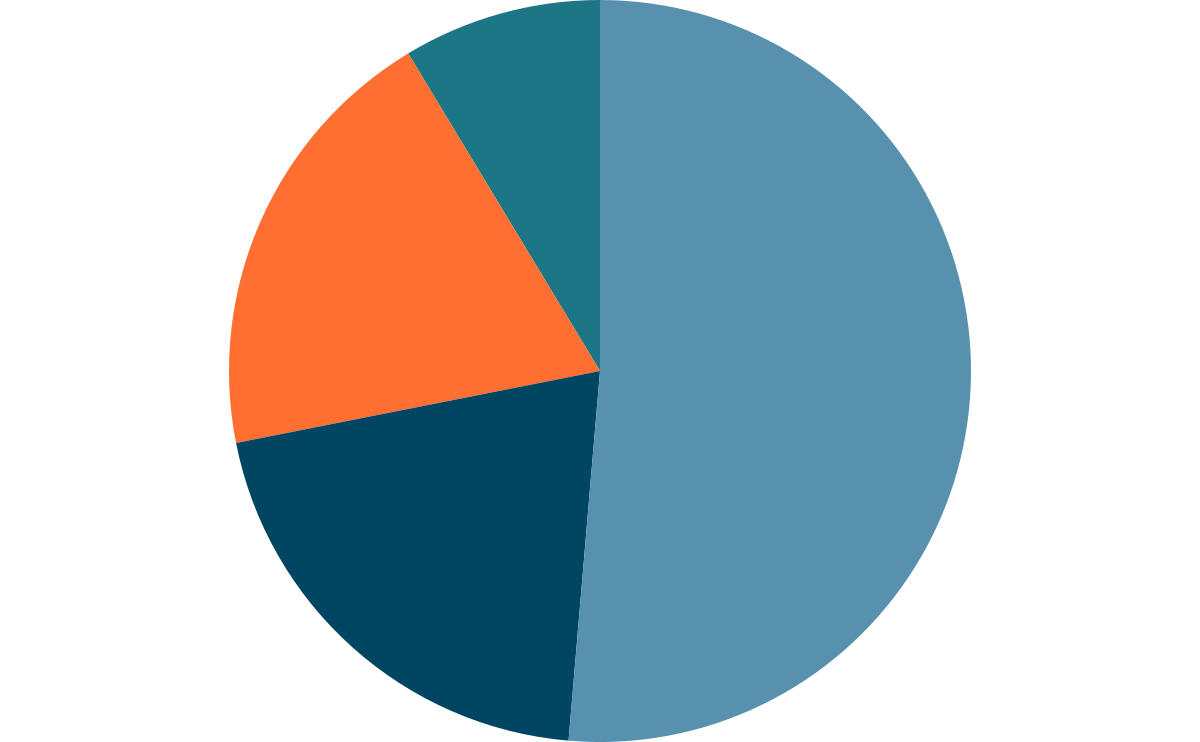 | 82 | 37 | 460 | 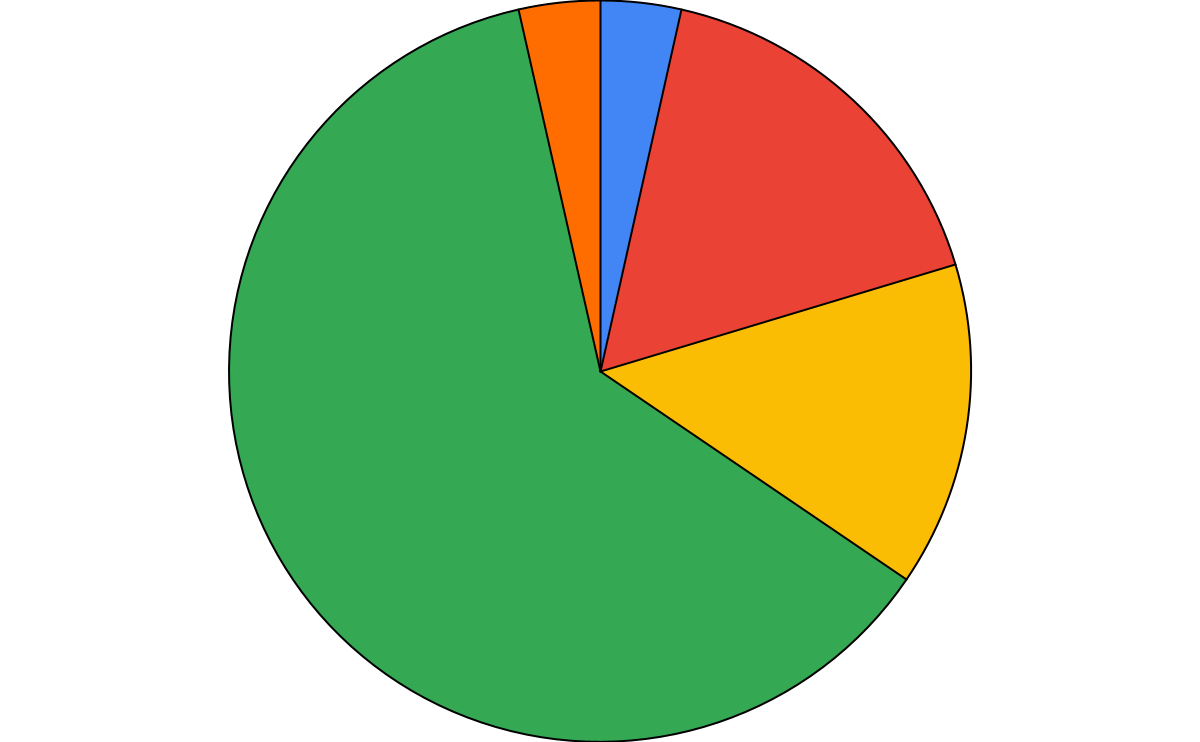 | 113 |
| FMT a | PacBio | 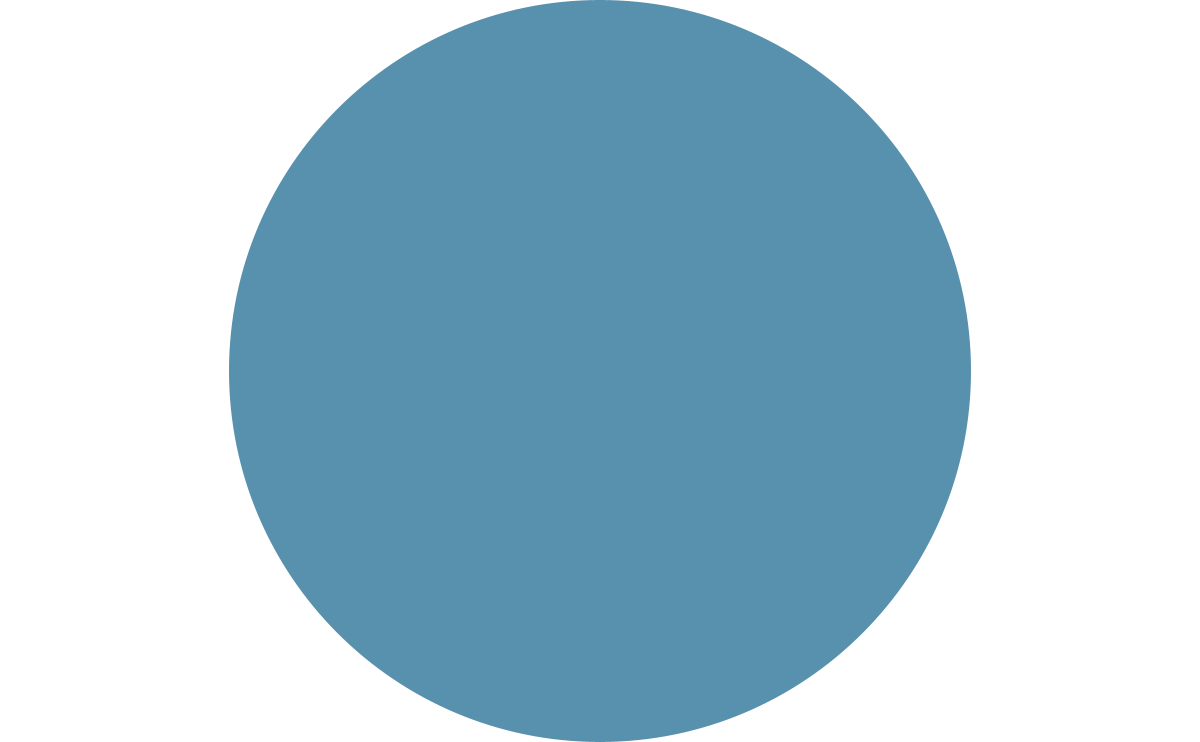 | 10 | 7 | 30 | 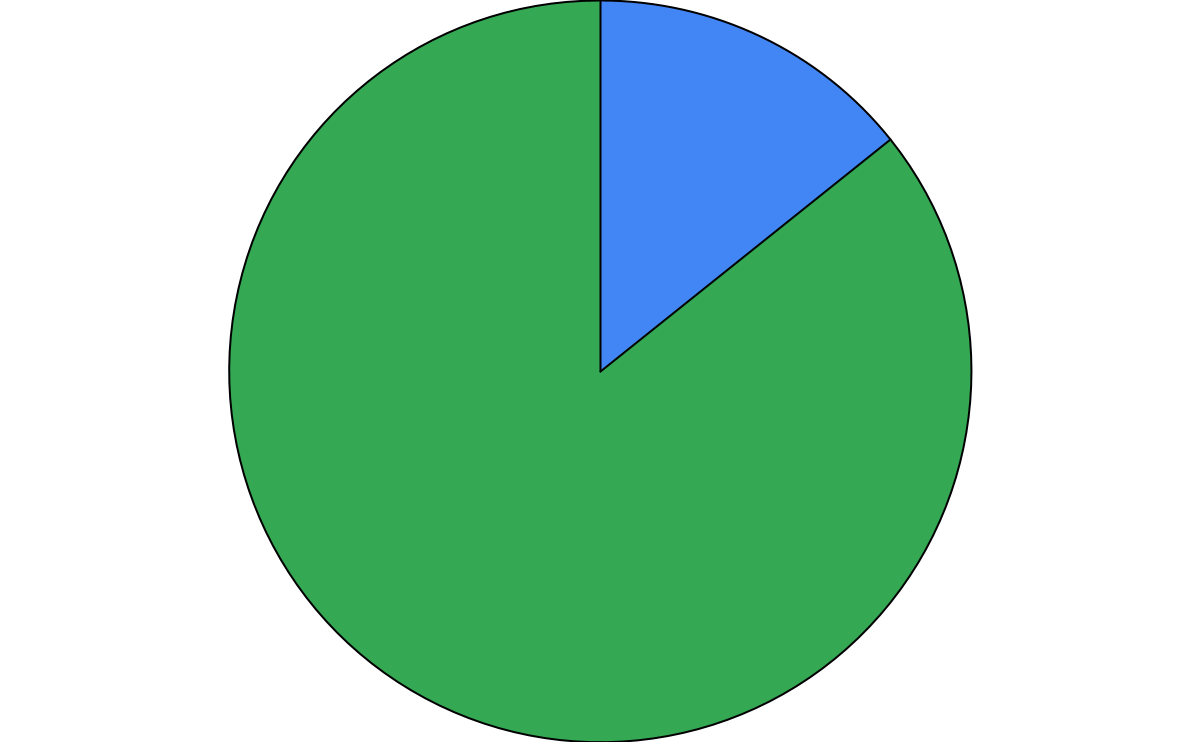 | 34 |
| FMT b | PacBio | 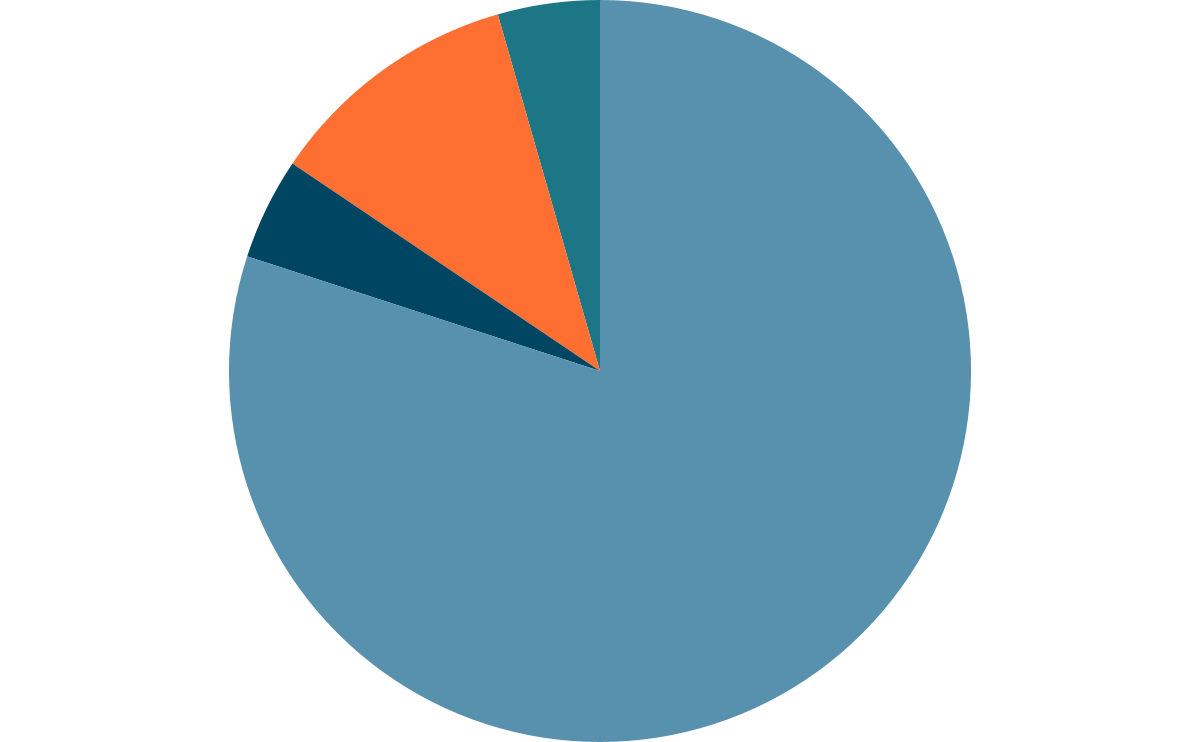 | 25 | 17 | 94 | 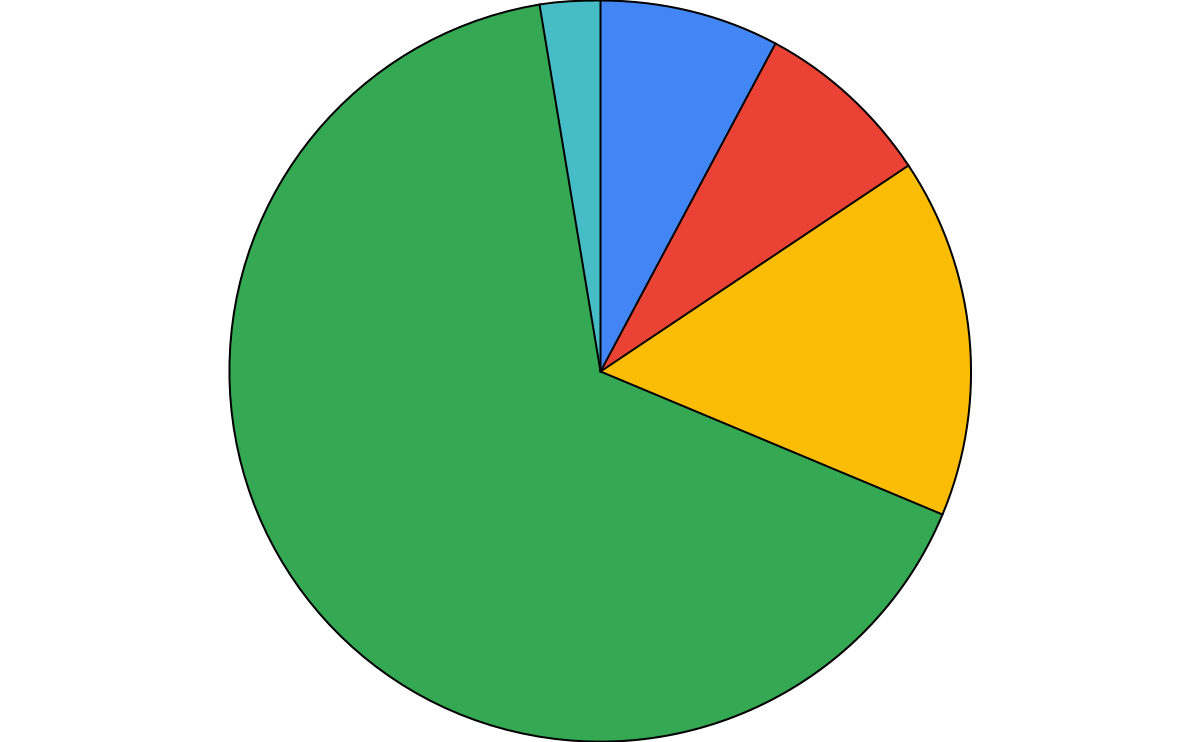 | 34 |
| FMT c | PacBio | 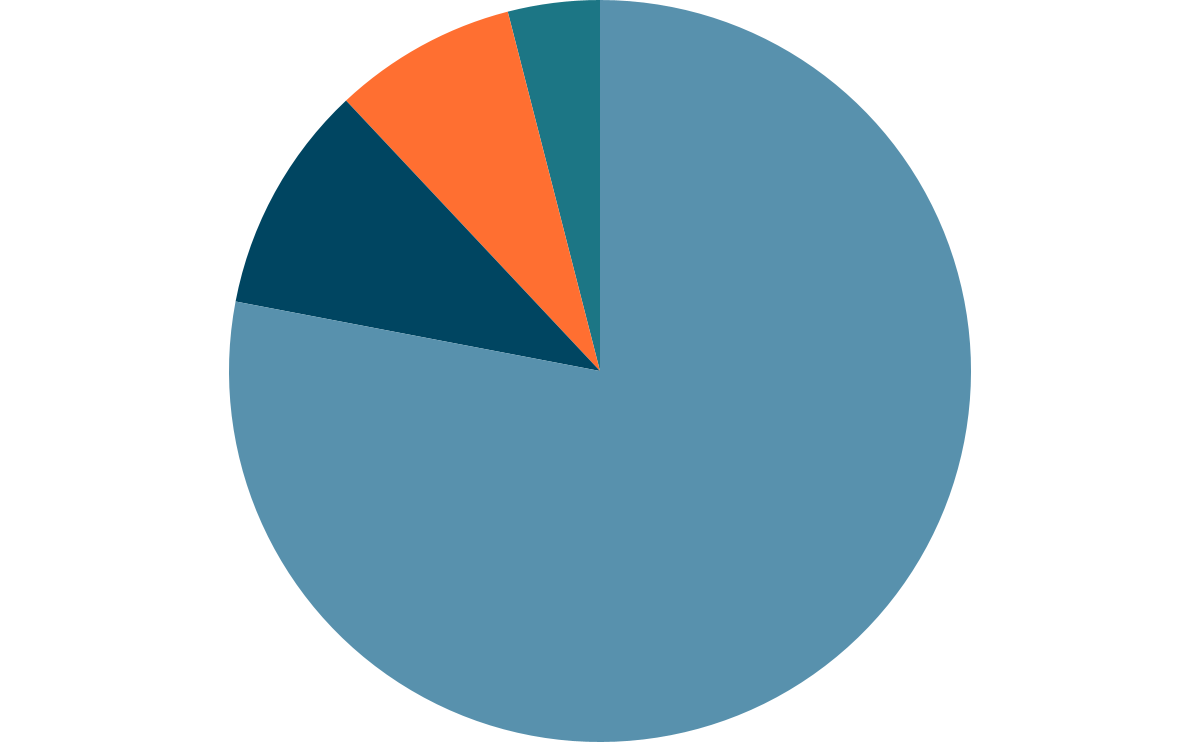 | 23 | 17 | 100 | 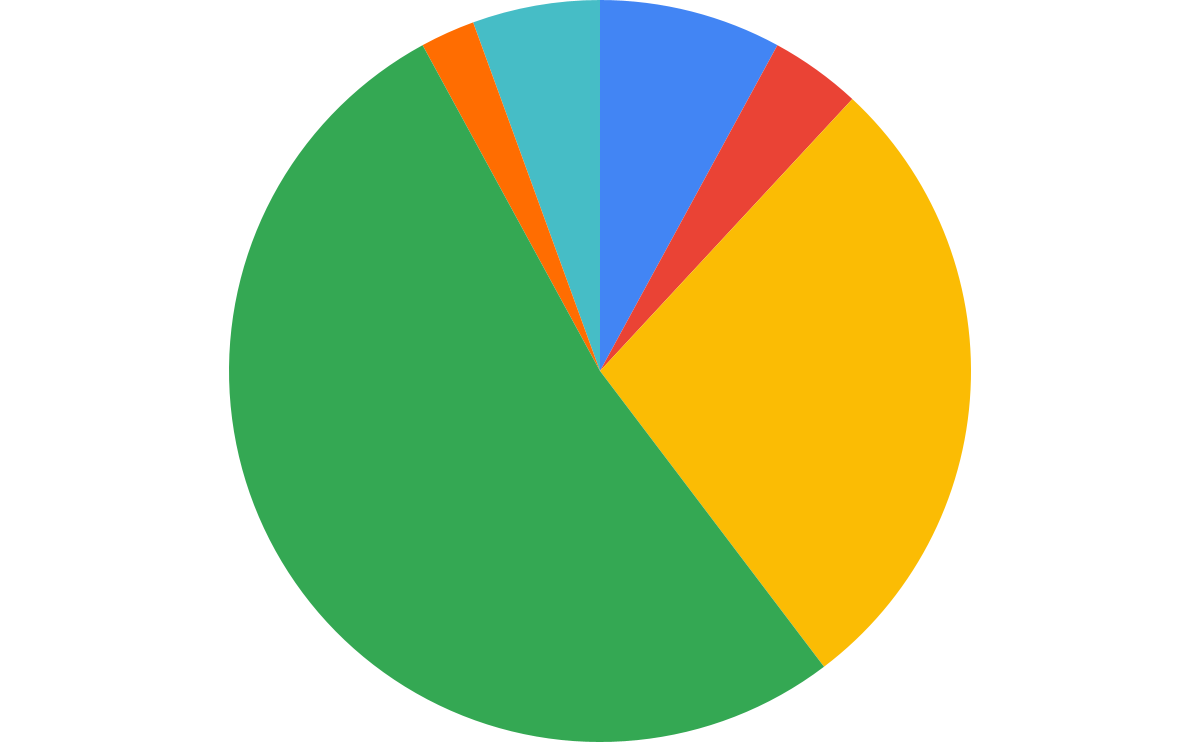 | 30 |
| FMT a | Illumina | 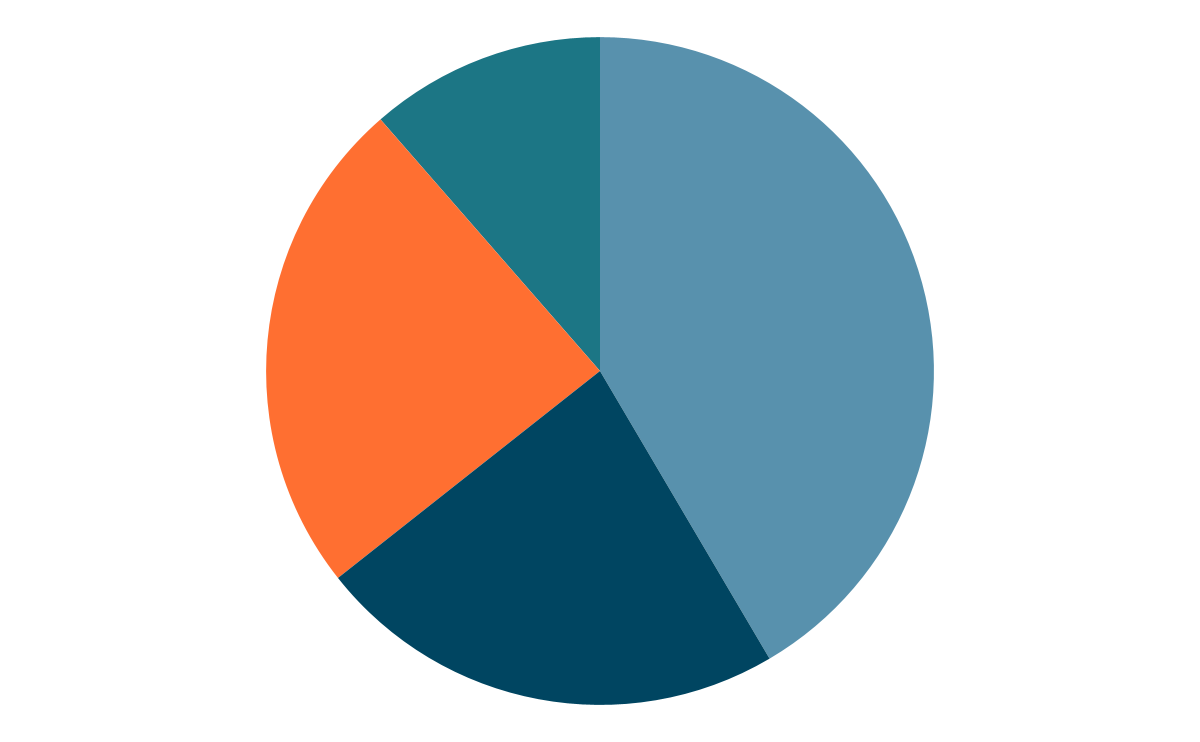 | 64 | 29 | 179 | 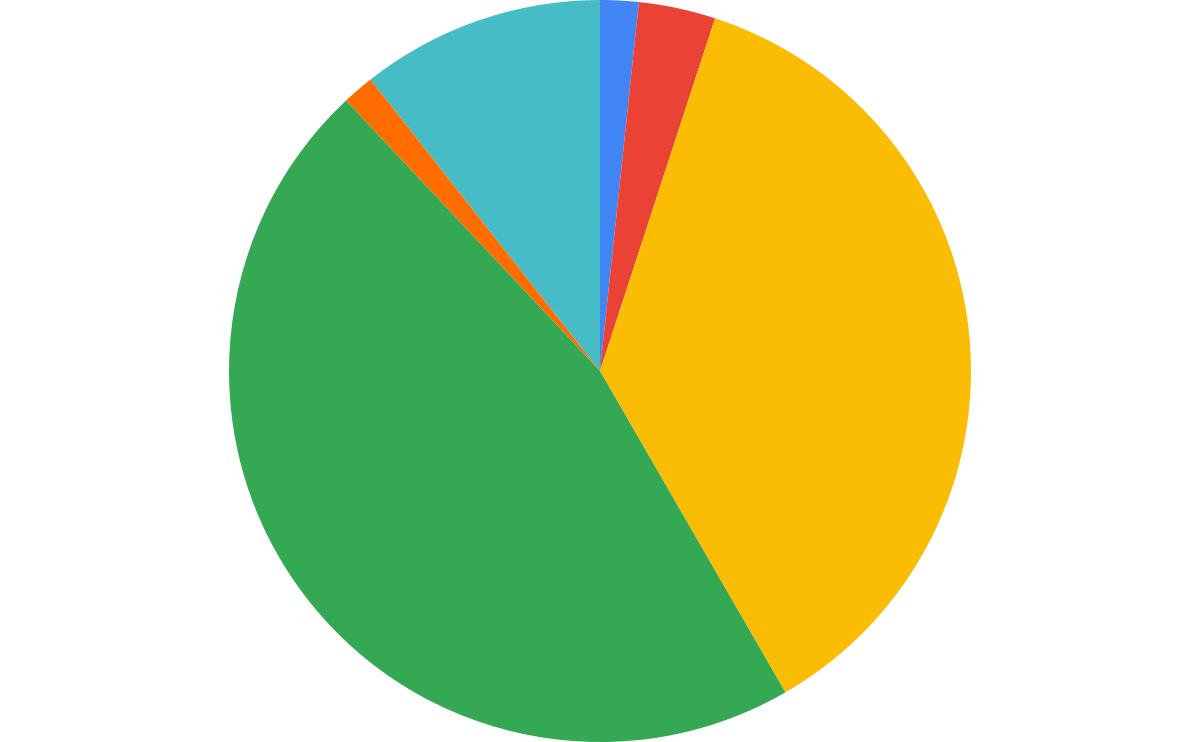 | 1,348 |
| FMT b | Illumina | 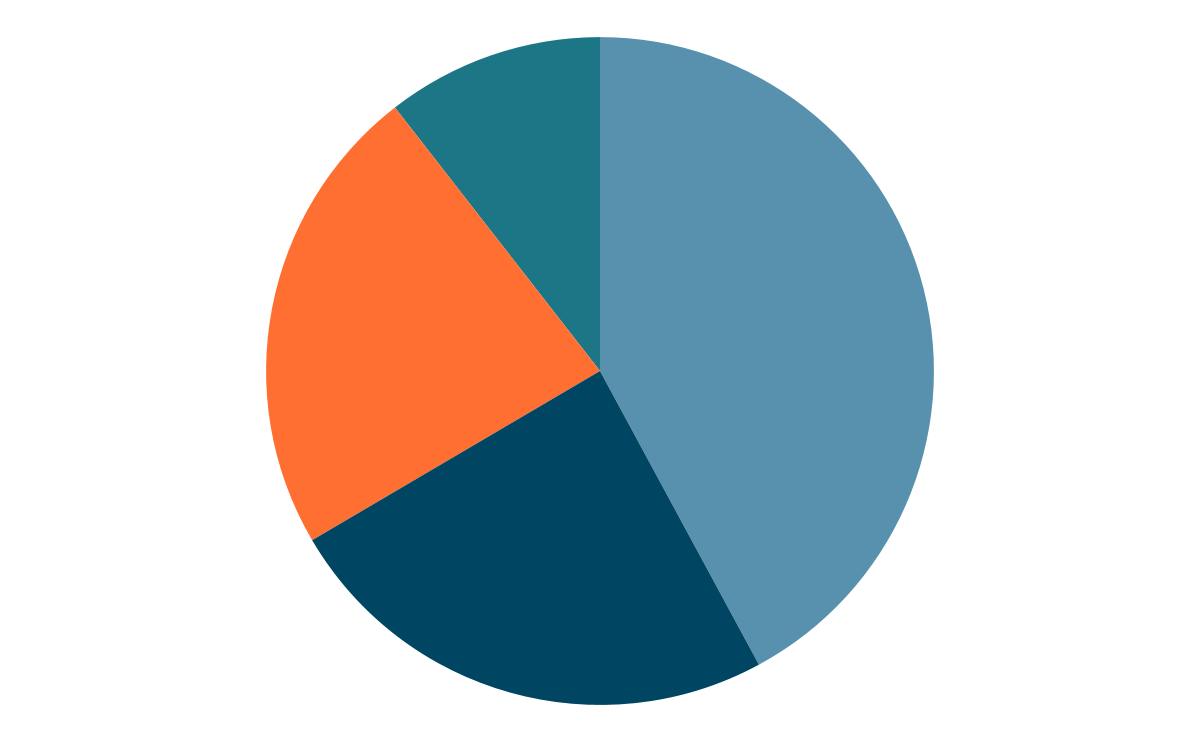 | 60 | 27 | 171 | 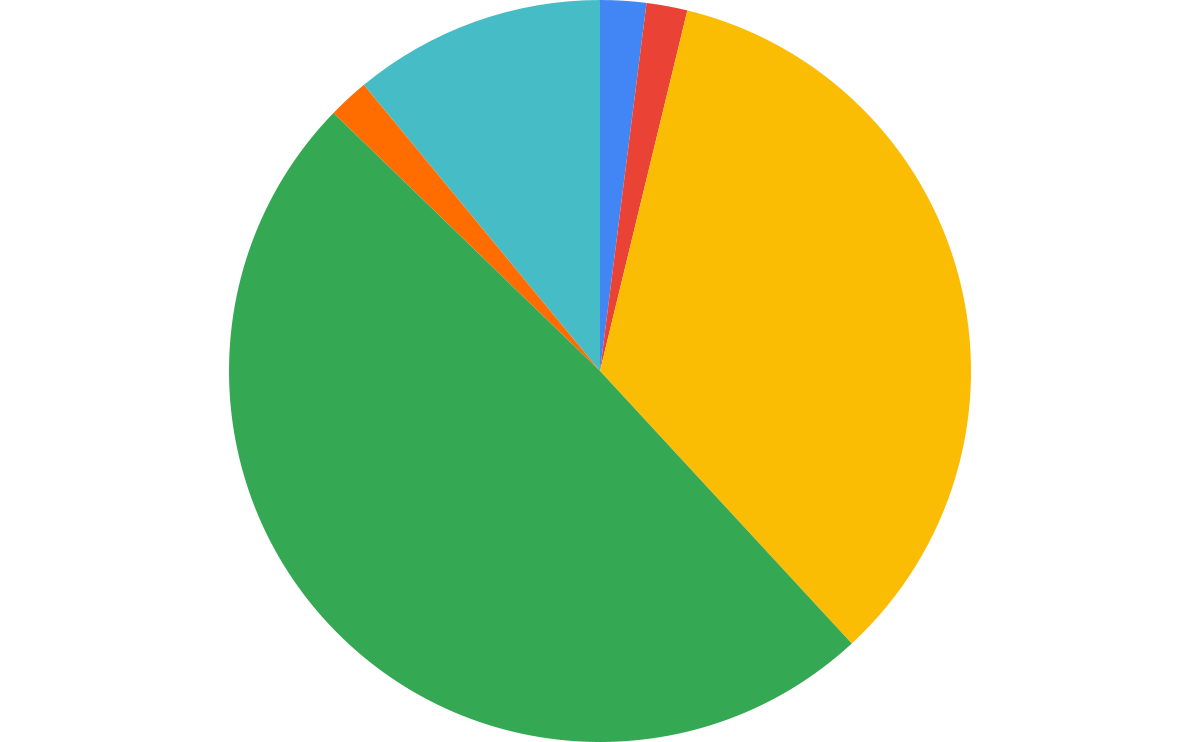 | 1,375 |
| FMT c | Illumina | 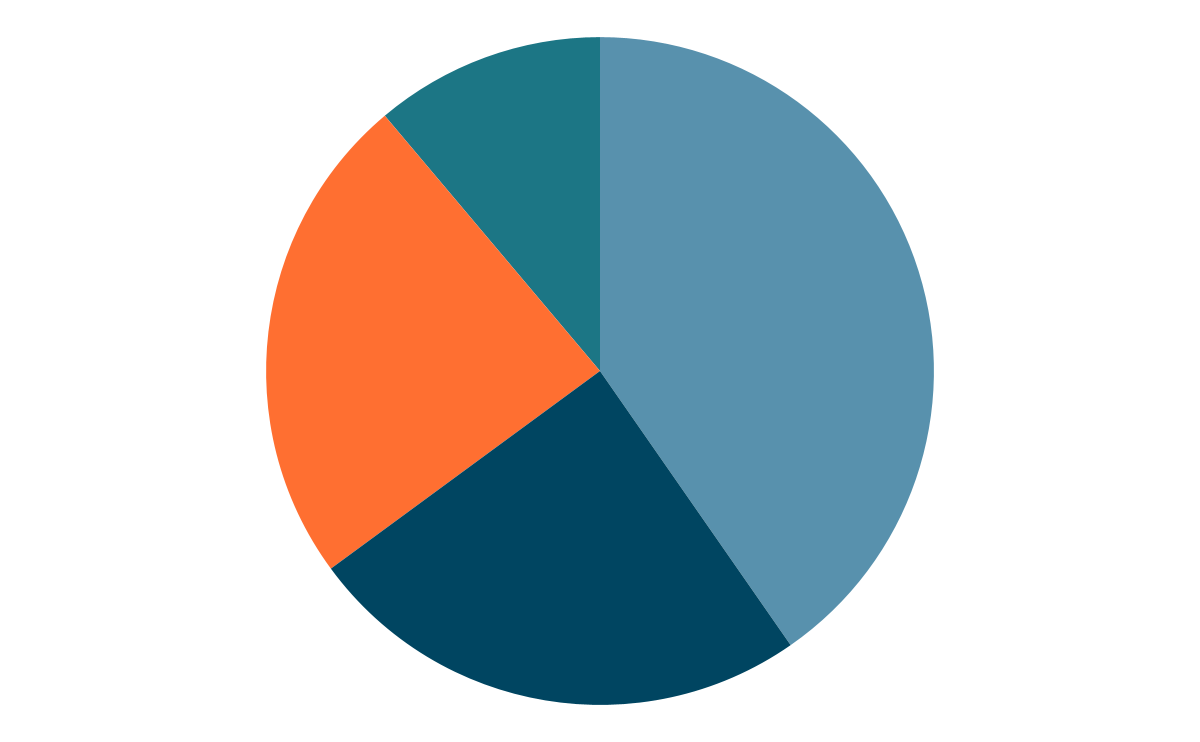 | 61 | 28 | 179 | 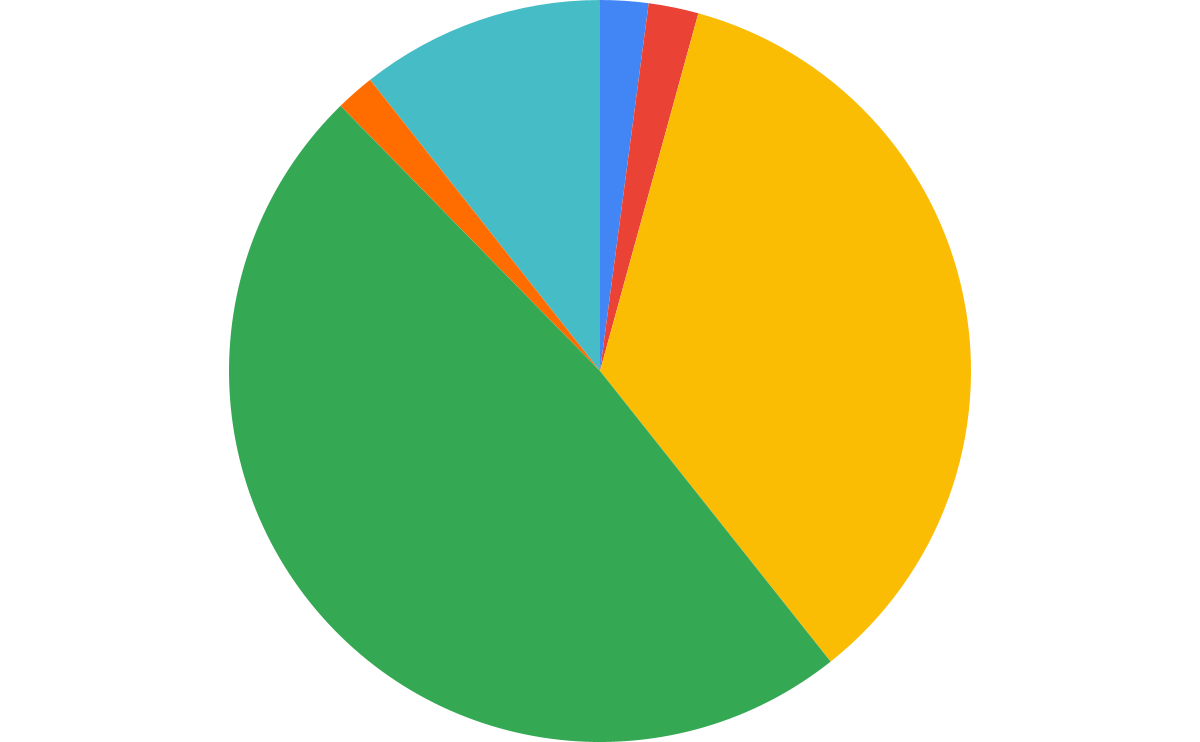 | 1,241 |
| SOIL a | TELSeq | 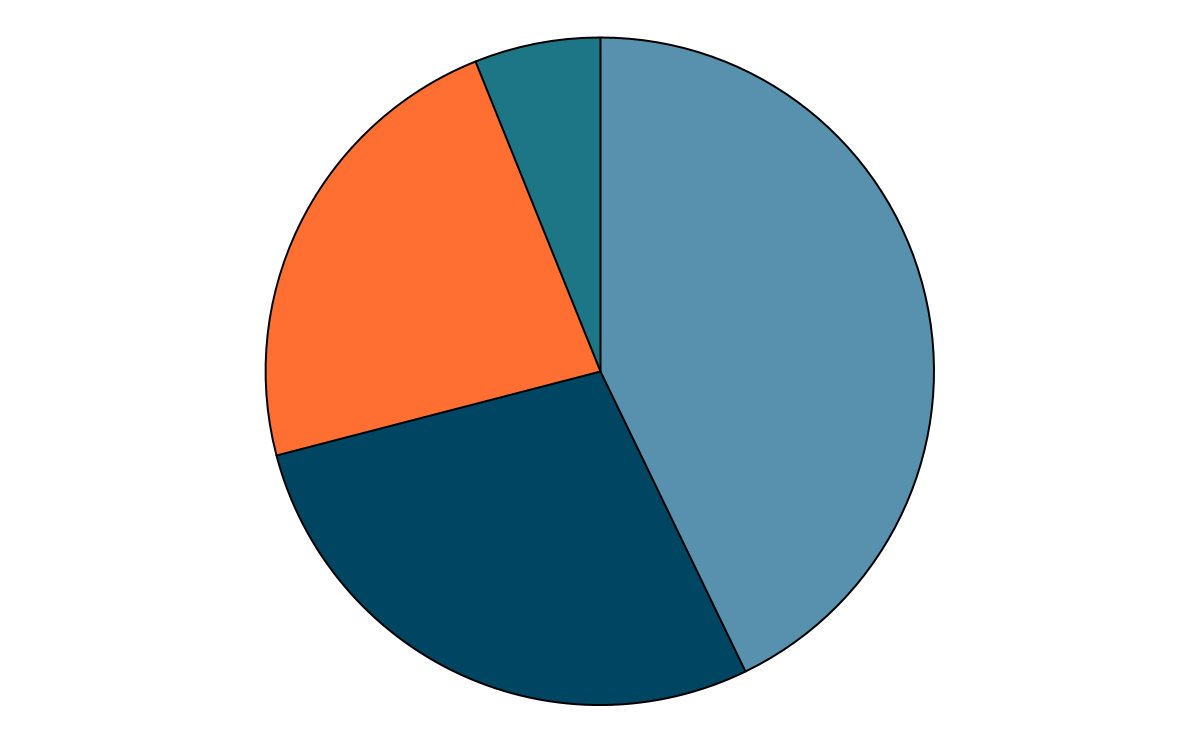 | 86 | 37 | 443 | 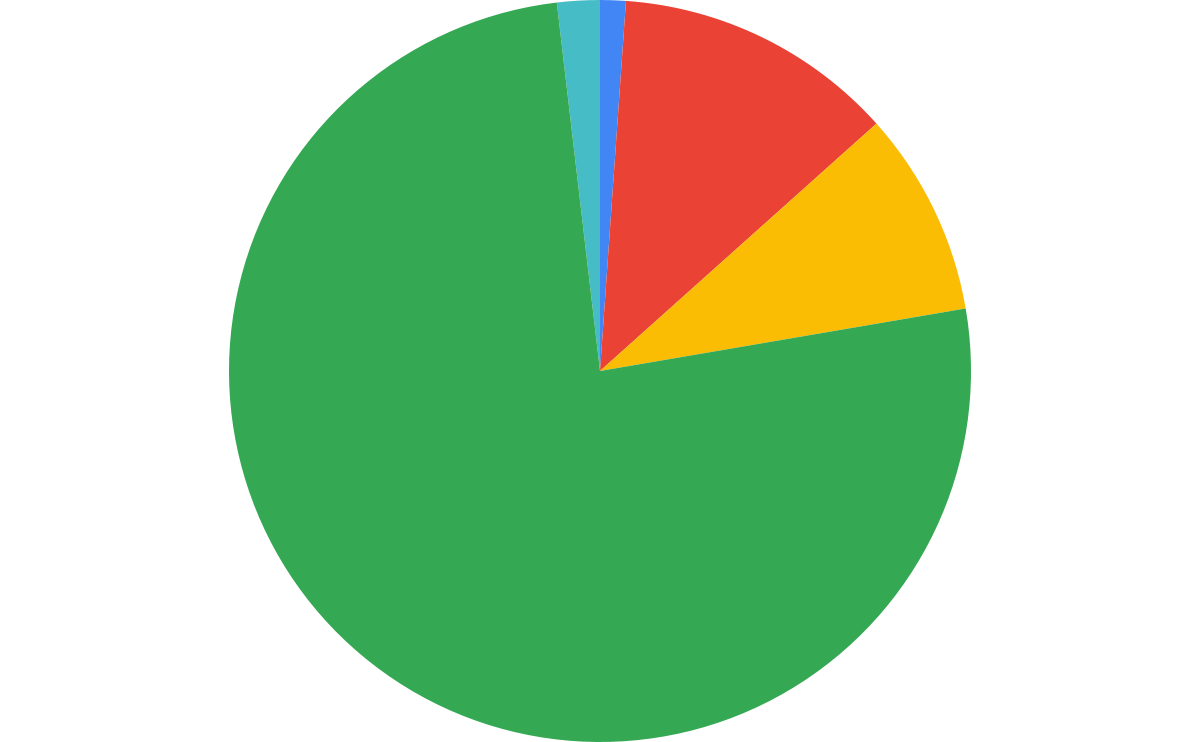 | 279 |
| SOIL b | TELSeq | 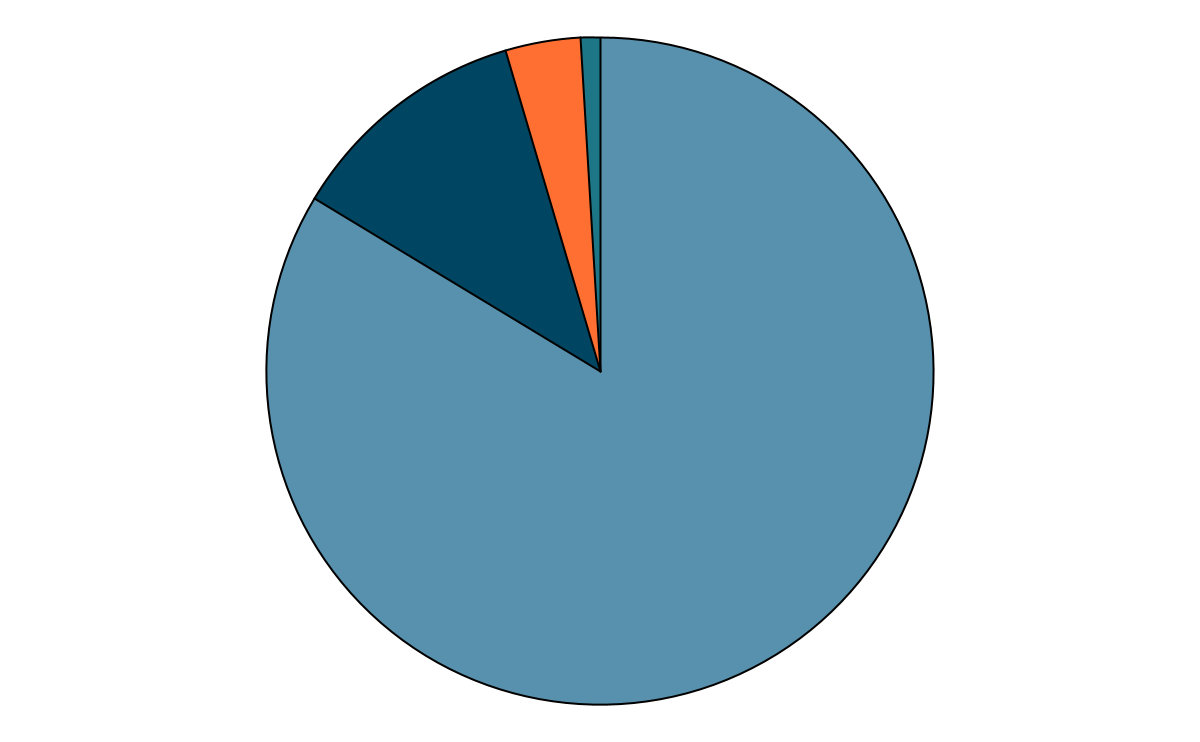 | 52 | 29 | 206 | 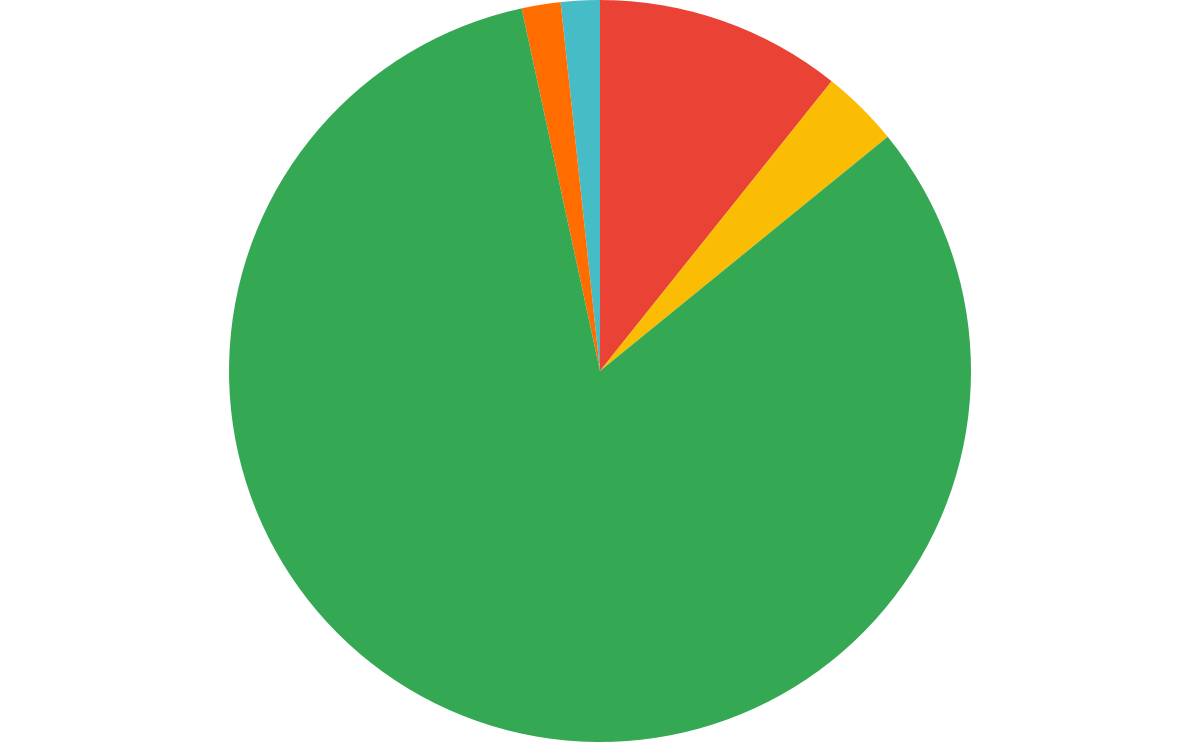 | 186 |
| SOIL c | TELSeq | 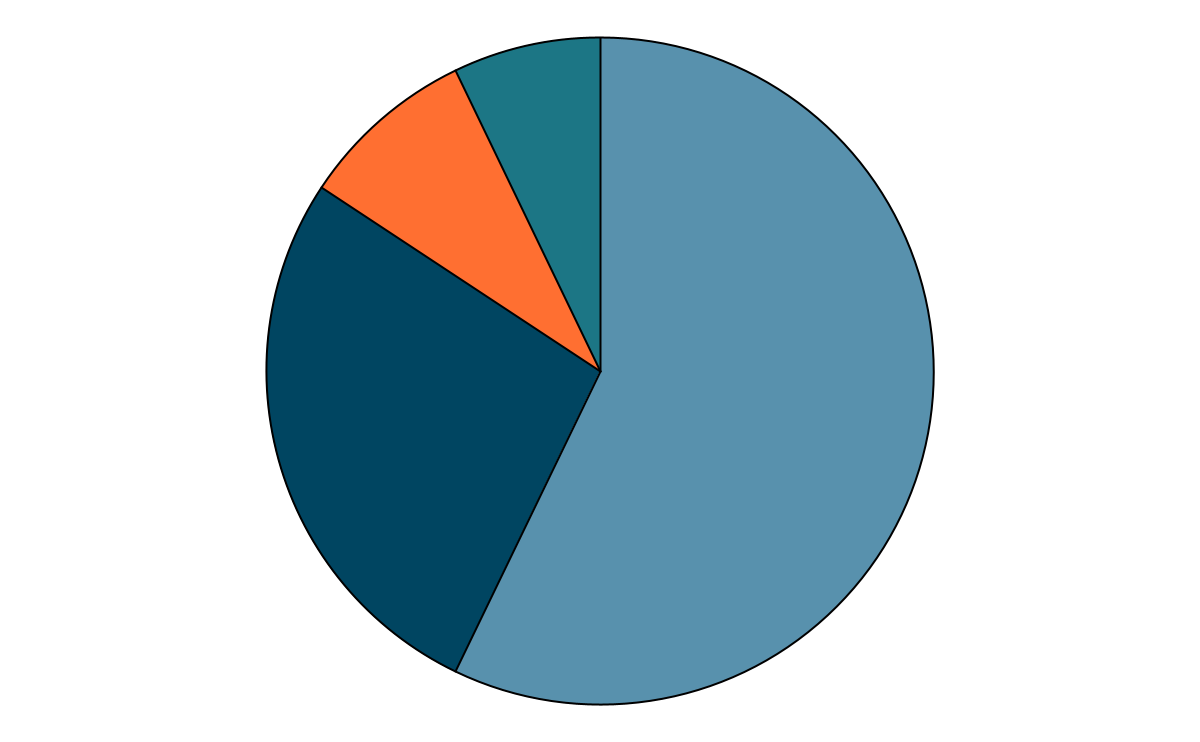 | 49 | 29 | 148 | 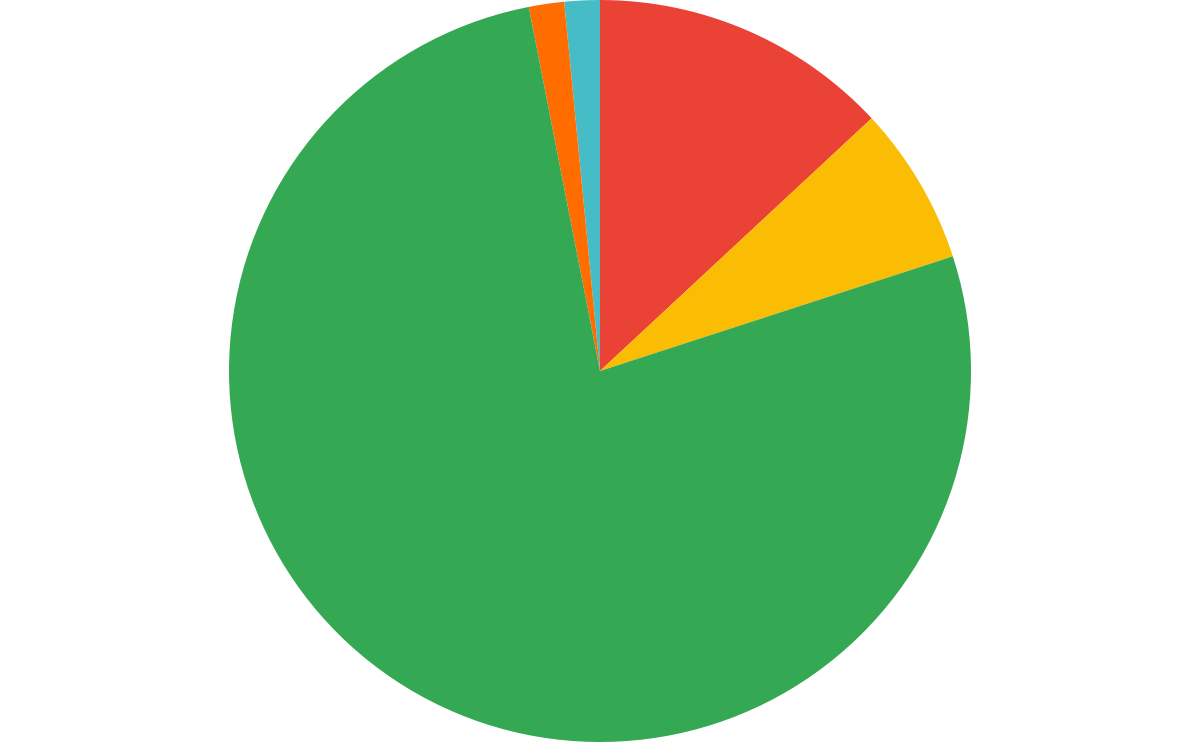 | 132 |
| SOIL a | PacBio | 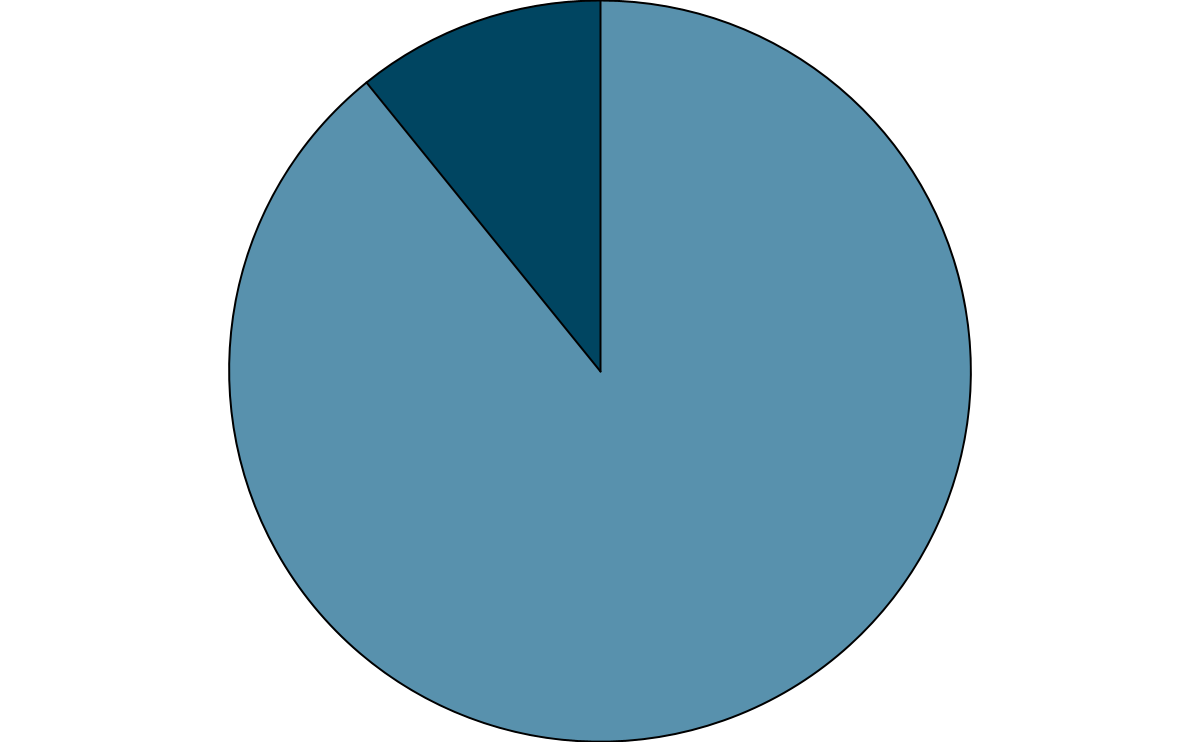 | 12 | 12 | 37 | 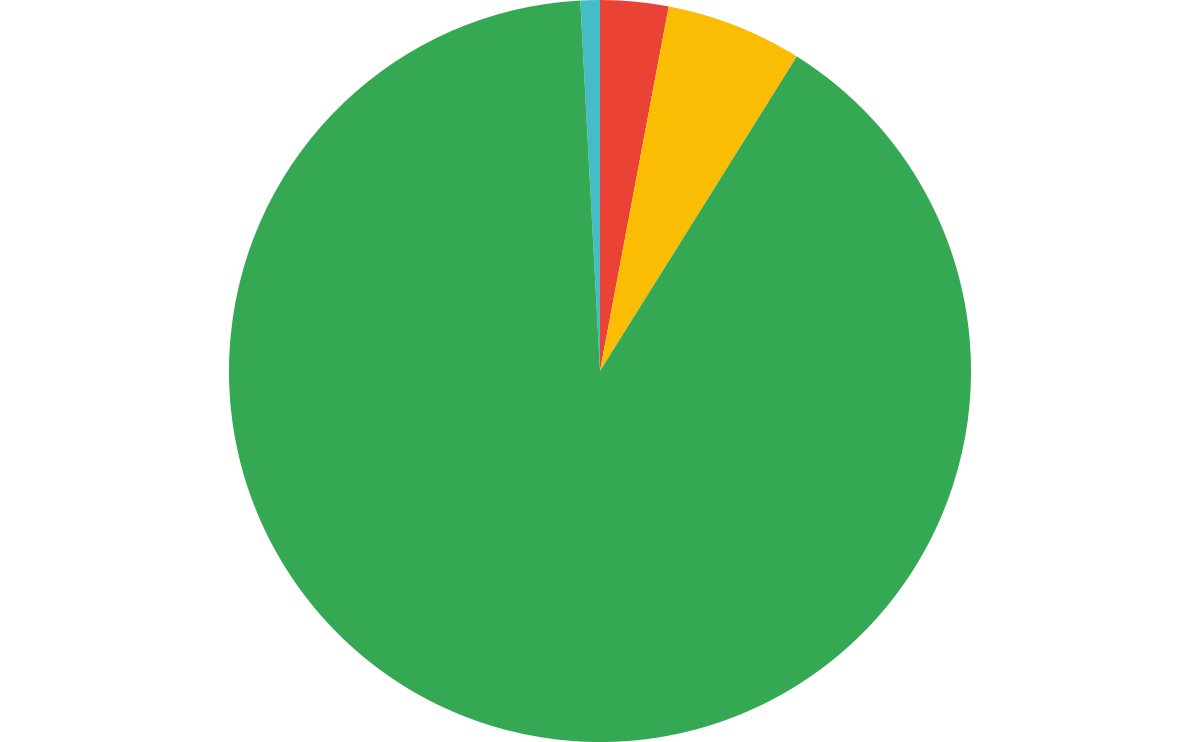 | 240 |
| SOIL b | PacBio | 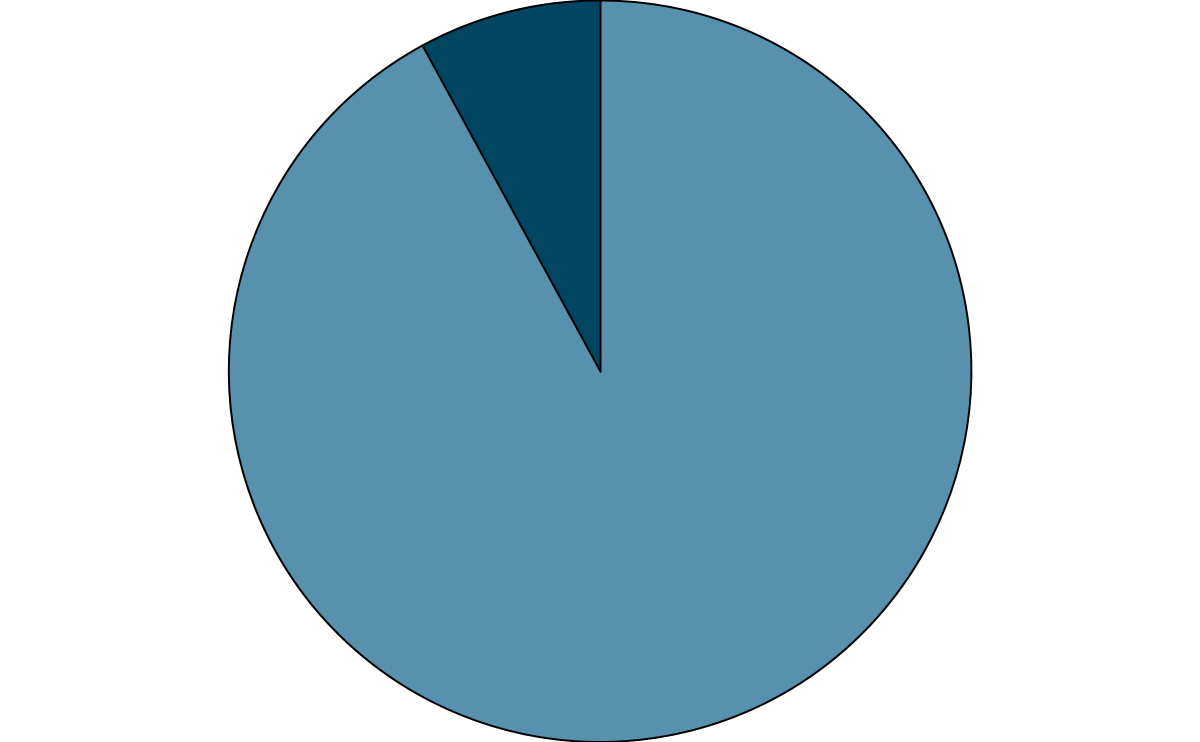 | 21 | 15 | 63 | 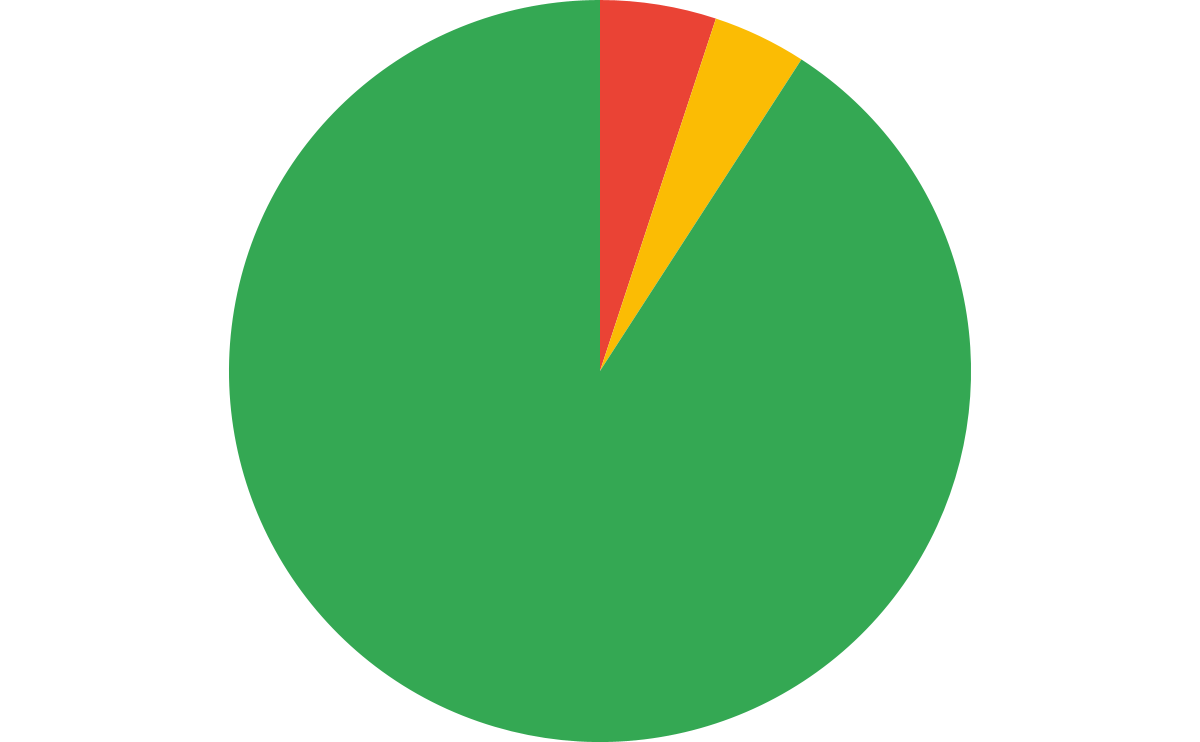 | 623 |
| SOIL c | PacBio | 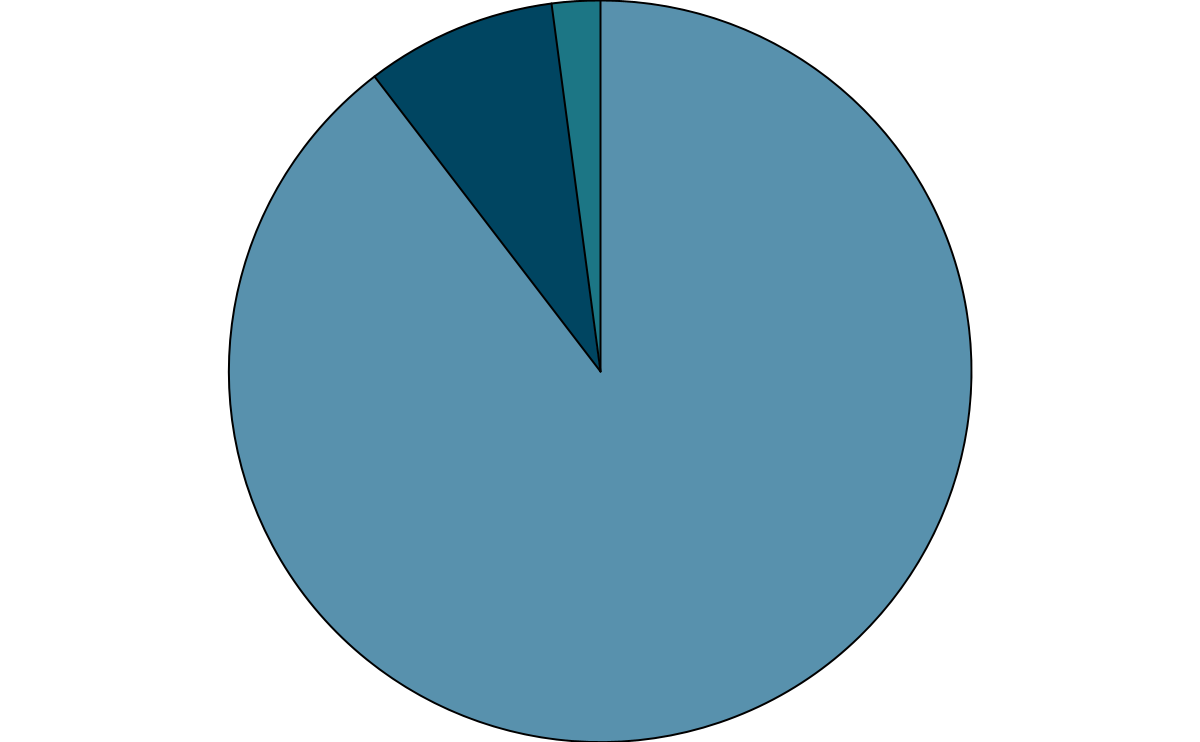 | 18 | 14 | 48 | 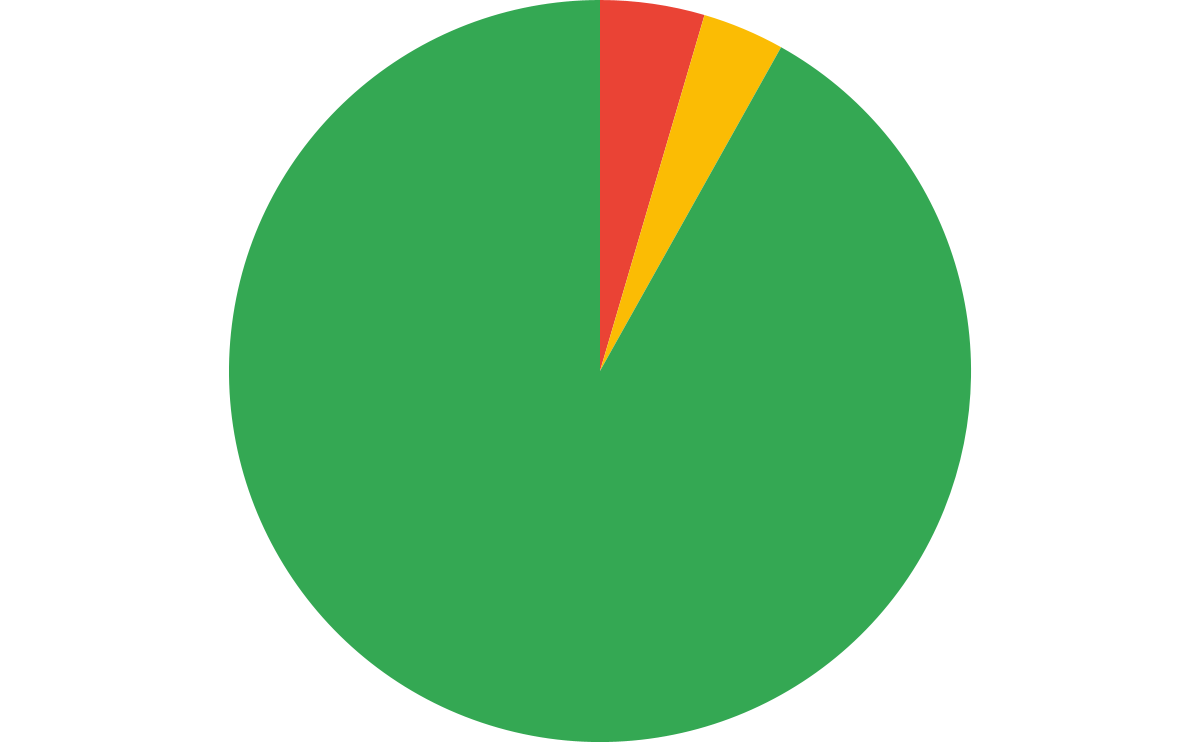 | 314 |
| SOIL a | Illumina | 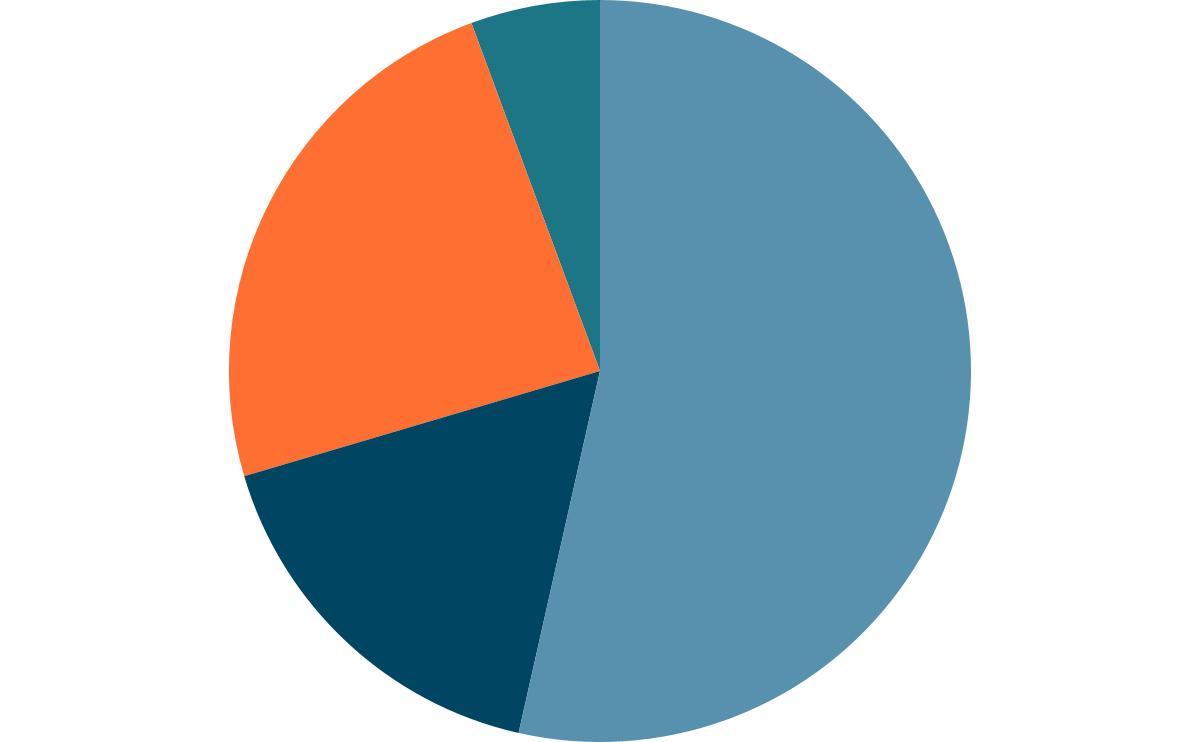 | 43 | 23 | 66 | 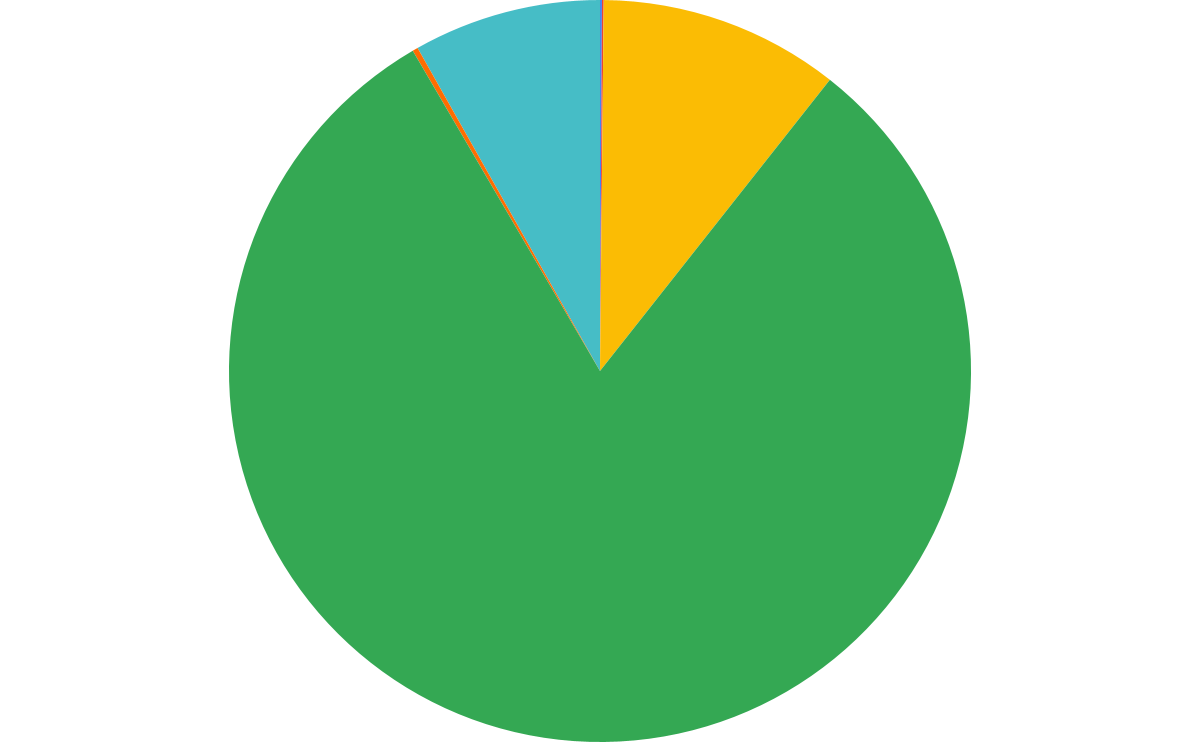 | 16,389 |
| SOIL b | Illumina | 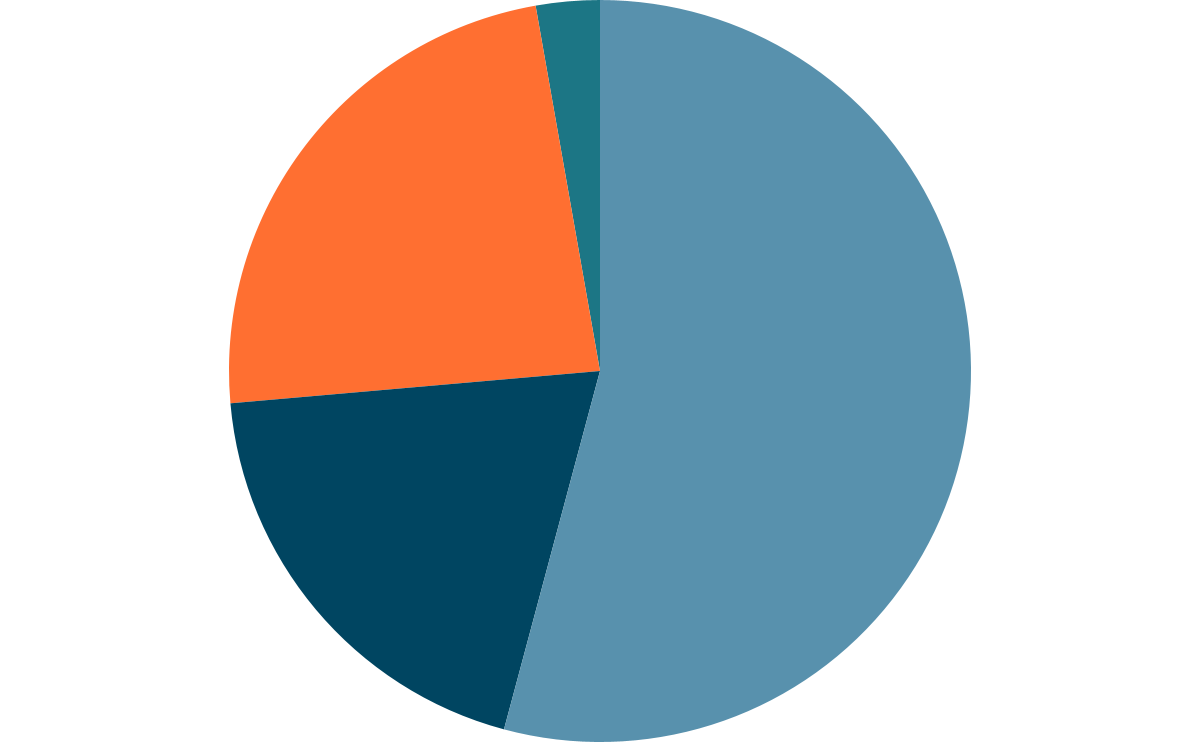 | 36 | 21 | 60 | 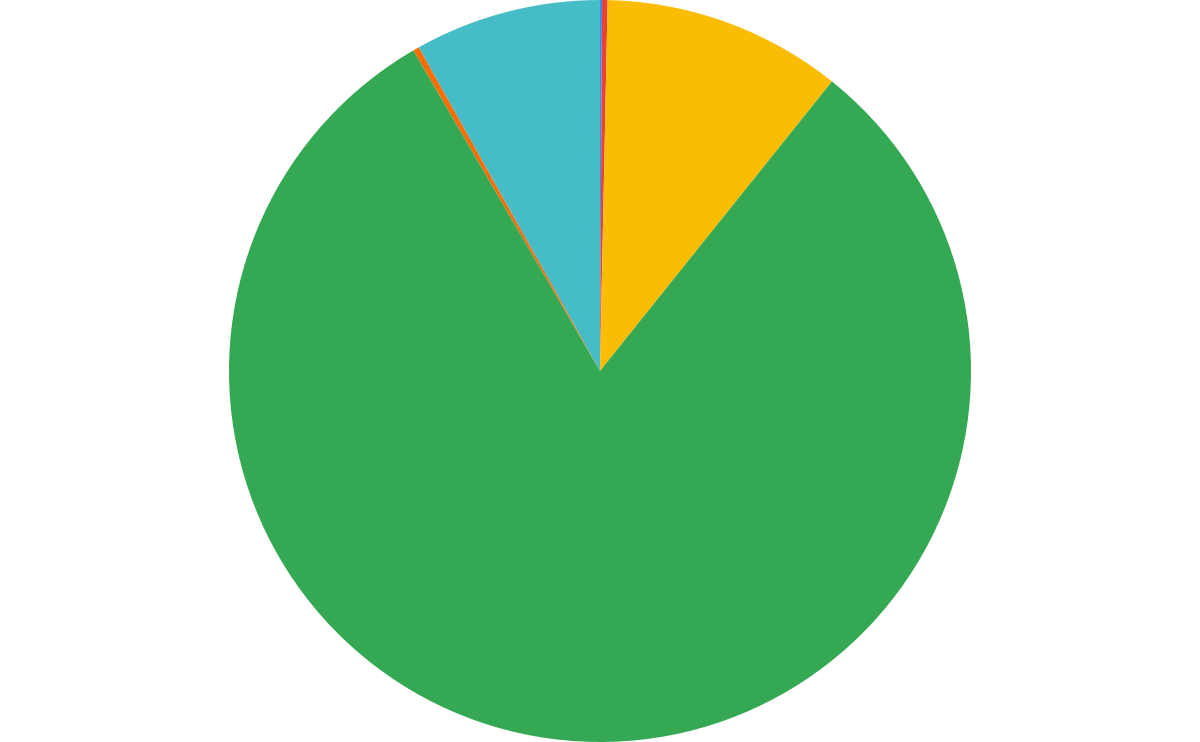 | 17,090 |
| SOIL c | Illumina | 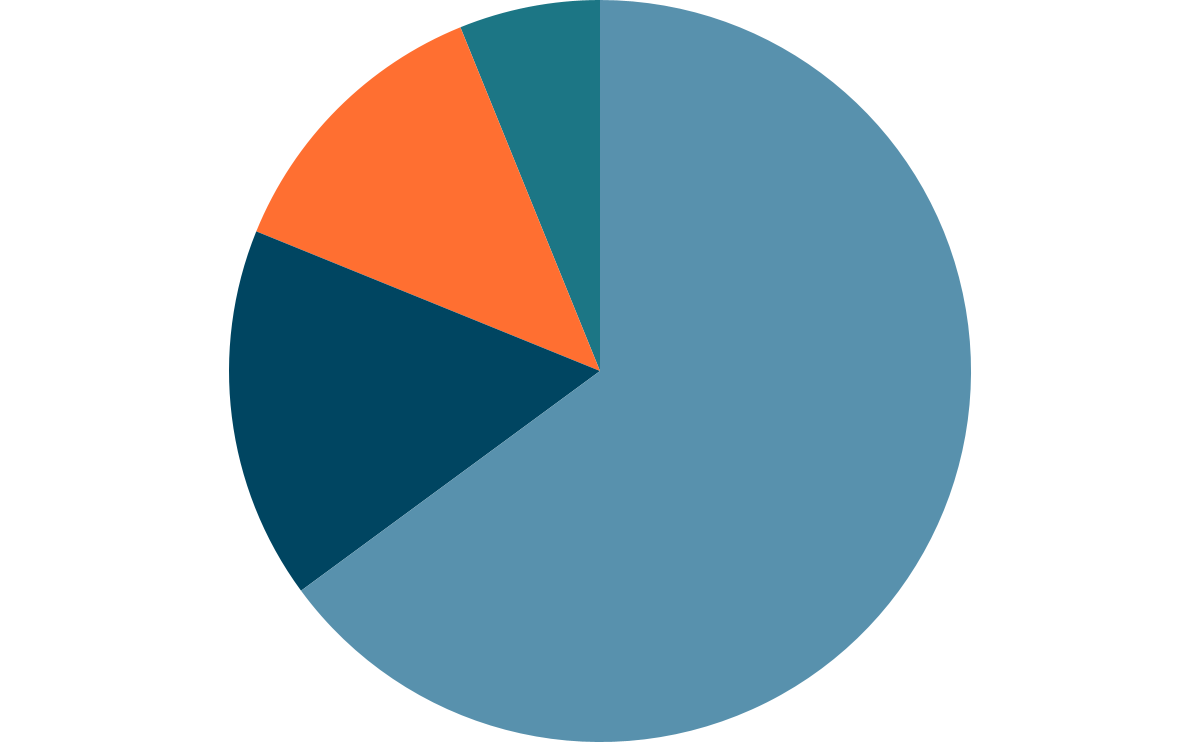 | 38 | 19 | 64 | 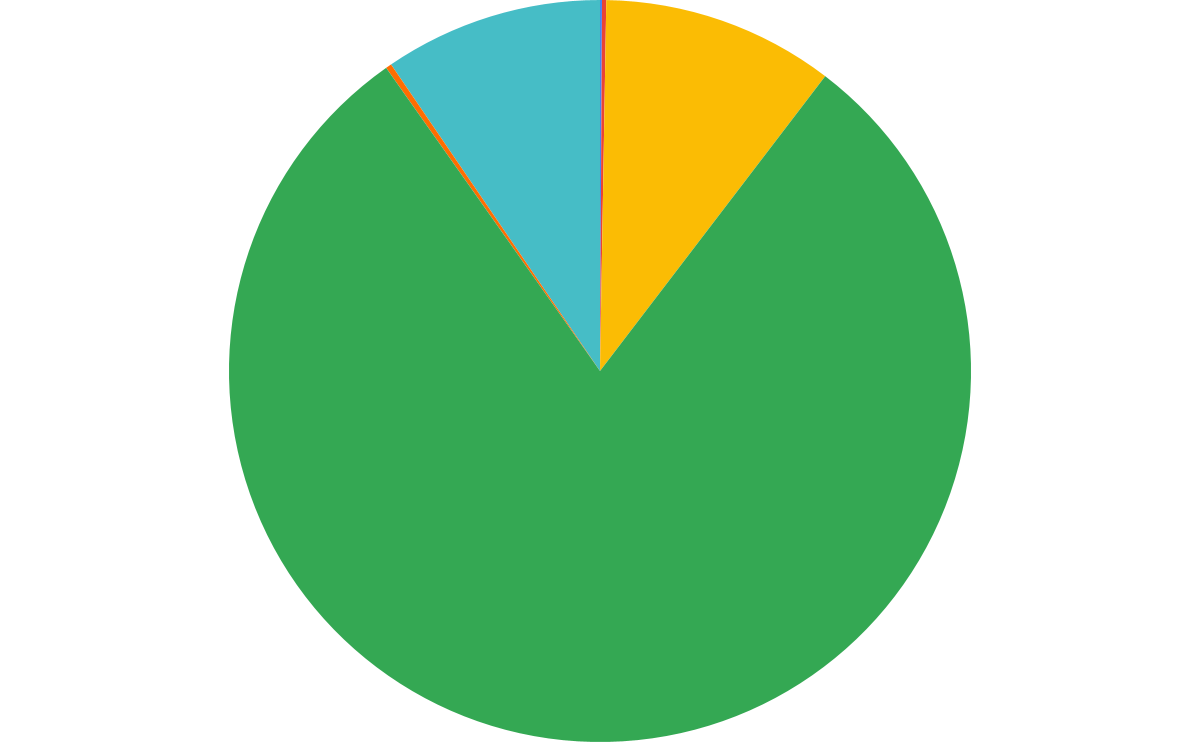 | 16,498 |
