# Supplementary material for: Target-enriched long-read sequencing (TELSeq) contextualizes antimicrobial resistance genes in metagenomes
Source: Microbiome. 2022 Nov 2;10:185. doi: 10.1186/s40168-022-01368-y (PMC9628182; doi:10.1186/s40168-022-01368-y)
Supplement: Supplementary file 8 — Additional file 7: Supplementary Table 3. TELSeq sequencing sensitivity, sequencing specificity, and excess read depth, by genome and MOCK replicate (2kb, 5kb and 8kb). [file 40168_2022_1368_MOESM7_ESM.docx]

**Supplementary Table 3.** TELSeq sequencing sensitivity, sequencing specificity, and excess read depth, by genome and MOCK replicate (2kb, 5kb and 8kb).

|  | **Genome size (Mbp)** | **% bases covered by probes** | **Proportion *(%)* of ARG groups recovered** | | | **Sensitivity (%)^a^** | | | **Specificity (%)^b^** | | | **TELSeq versus GridION Depth (%)^c^** | | | **TELSeq versus PromethION Depth (%)^d^** | | |
| --- | --- | --- | --- | --- | --- | --- | --- | --- | --- | --- | --- | --- | --- | --- | --- | --- | --- |
| **Organism** |  |  | **2 kb** | **5 kb** | **8 kb** | **2 kb** | **5 kb** | **8 kb** | **2 kb** | **5 kb** | **8 kb** | **2 kb** | **5 kb** | **8 kb** | **2 kb** | **5 kb** | **8 kb** |
| *L. monocytogenes* | 2.99 | 0.22% | 2/2 *(100%)* | 2/2 *(100%)* | 2/2 *(100%)* | 100.0 | 100.0 | 100.0 | 26.8 | 26.1 | 26.0 | 24.2 | 0.0 | 19.7 | 0.0 | 0.00 | 0.0 |
| *P. aeruginosa* | 6.79 | 1.26% | 20/30 *(67%)* | 13/30 *(43%)* | 15/30 *(50%)* | 85.5 | 65.1 | 70.6 | 60.4 | 78.3 | 72.7 | 29.7 | 18.5 | 23.4 | 59.7 | 27.1 | 32.5 |
| *B. subtilis* | 4.05 | 0.40% | 6/6 *(100%)* | 6/6 *(100%)* | 6/6 *(100%)* | 100.0 | 100.0 | 100.0 | 86.9 | 84.1 | 79.7 | 78.4 | 77.6 | 77.6 | 35.5 | 23.8 | 45.4 |
| *E. coli* | 4.88 | 4.45% | 35/38 *(92%)* | 35/38 *(92%)* | 36/38 *(95%)* | 100.0 | 100.0 | 100.0 | 75.4 | 69.6 | 59.1 | 99.1 | 98.8 | 99.4 | 79.9 | 70.2 | 77.3 |
| *S. enterica* | 4.76 | 3.35% | 28/30 *(93%)* | 27/30 *(90%)* | 27/30 *(90%)* | 100.0 | 99.7 | 100.0 | 76.4 | 71.8 | 62.0 | 96.3 | 95.2 | 96.4 | 78.1 | 76.3 | 80.7 |
| *E. faecalis* | 2.85 | 0.94% | 1/3 *(33%)* | 1/3 *(33%)* | 1/3 *(33%)* | 76.6 | 72.8 | 79.3 | 97.7 | 97.2 | 96.6 | 45.7 | 53.4 | 51.3 | 39.5 | 41.2 | 45.6 |
| *S. aureus* | 2.73 | 1.28% | 0/15 *(0%)* | 3/15 *(20%)* | 1/15 *(1%)* | 30.5 | 31.5 | 45.0 | 98.7 | 98.5 | 97.9 | 16.7 | 18.0 | 27.3 | 14.6 | 17.8 | 27.2 |
| *L. fermentum* | 1.91 | 0.00% | -- | -- | -- | -- | -- | -- | 99.3 | 99.3 | 99.2 | -- | -- | -- | -- | -- | -- |
| *S. cerevisiae* | 12.10 | 0.00% | -- | -- | -- | -- | -- | -- | 95.9 | 95.1 | 92.6 | -- | -- | -- | -- | -- | -- |
| *C. neoformans* | 18.90 | 0.00% | -- | -- | – | -- | -- | – | 99.9 | 99.9 | 99.9 | -- | -- | -- | -- | -- | -- |

^a^Sequencing sensitivity was defined as the proportion of probe-covered bases that received TELSeq read coverage of at least 1X.

^b^Sequencing specificity was defined as the proportion of non-probe-covered bases that did not receive any TELSeq read coverage.

^c^TELSeq versus GridION depth was defined as the proportion of probe-covered bases for which TELSeq read depth exceeded that of GridION read depth.

^d^TELSeq versus PromethION depth was defined as the proportion of probe-covered bases for which TELSeq read depth exceeded that of GridION read depth.
