# Supplementary material for: Target-enriched long-read sequencing (TELSeq) contextualizes antimicrobial resistance genes in metagenomes
Source: Microbiome. 2022 Nov 2;10:185. doi: 10.1186/s40168-022-01368-y (PMC9628182; doi:10.1186/s40168-022-01368-y)
Supplement: Supplementary file 9 — Additional file 8 Supplementary Table 4. Summary of species distribution and previously generated sequencing results for the ZymoBIOMICSTM mock microbial community standard (CSII) in logarithmic abundance. [file 40168_2022_1368_MOESM8_ESM.docx]

**Supplementary Table 4.** Summary of species distribution and previously generated sequencing results for the ZymoBIOMICS^TM^ mock microbial community standard (CSII) in logarithmic abundance.

|  |  |  |  |  |  |  | **GridION^c^** | | | **PromethION^d^** | | |
| --- | --- | --- | --- | --- | --- | --- | --- | --- | --- | --- | --- | --- |
| **Species** | **%** | **Gram** | **Genome Size (Mb)** | **NRRL^a^ Ref.** | **ATCC^b^ Ref.** | **ST^c^ Ref.** | **Yield**  **(Gb)** | **N50**  **(kb)** | **Coverage**  **(X)** | **Yield**  **(Gb)** | **N50**  **(kb)** | **Coverage**  **(X)** |
| *Listeria monocytogenes* | 89.1 | + | 2.99 | B-33116 | 19117 | 449 | 12.10 | 4.95 | 4043.90 | 110.09 | 4.97 | 36796.21 |
| *Pseudomonas aeruginosa* | 8.9 | - | 6.79 | B-3509 | 15442 | 252 | 1.10 | 9.38 | 161.45 | 9.99 | 9.33 | 1471.41 |
| *Bacillus subtilis* | 0.89 | + | 4.05 | B-354 | 6633 | 7 | 0.16 | 5.03 | 38.67 | 1.44 | 5.04 | 356.00 |
| *Saccharomyces cerevisiae* | 0.89 | Euk. | 12.10 | Y-567 | 9763 | * | 0.08 | 4.78 | 6.93 | 0.75 | 4.75 | 62.33 |
| *Escherichia coli* | 0.089 | - | 4.88 | B-1109 | * | 10 | 0.01 | 9.20 | 2.20 | 0.1 | 9.17 | 20.04 |
| *Salmonella enterica* | 0.089 | - | 4.76 | B-4212 | * | 139 | 0.01 | 8.65 | 2.14 | 0.09 | 9.17 | 19.24 |
| *Lactobacillus fermentum* | 0.0089 | + | 1.91 | B-1840 | 14931 | * | 4E-4 | 3.40 | 0.21 | 0.004 | 3.37 | 2.03 |
| *Enterococcus faecalis* | 0.00089 | + | 2.85 | B-537 | 7080 | 55 | 2E-4 | 7.62 | 0.055 | 1E-3 | 6.05 | 0.34 |
| *Cryptococcus neoformans* | 0.00089 | Euk. | 18.90 | Y-2534 | 32045 | * | 6E-5 | 4.41 | 0.003 | 1E-4 | 4.97 | 0.037 |
| *Staphylococcus aureus* | 0.000089 | + | 2.73 | B-41012 | * | 9 | 1E-5 | 7.12 | 0.005 | 5E-5 | 3.58 | 0.02 |

Species-level and strain-level(a,b,c) distribution information taken from protocol v1.1.3 of the ZymoBIOMICS^TM^ microbial community standard (CSII) in log distribution. Sequenced throughput, coverage, and length metadata (c,d) were abstracted from experiments of Nicholls et al. (GridION FASTQ: ERR3152366; PromethION FASTQ: ERR3152367)
